# DNA methylation-regulated QPCT promotes sunitinib resistance by increasing HRAS stability in renal cell carcinoma

**DOI:** 10.7150/thno.35572

**Published:** 2019-08-14

**Authors:** Tangliang Zhao, Yi Bao, Xinxin Gan, Jie Wang, Qiong Chen, Zhihui Dai, Bing Liu, Anbang Wang, Shuhan Sun, Fu Yang, Linhui Wang

**Affiliations:** 1 Department of Urology, Changzheng Hospital, Second Military Medical University, Shanghai 200003, China.; 2 Department of Medical Genetics, Second Military Medical University, Shanghai 200433, China.; 3 Shanghai Key Laboratory of Cell Engineering, Second Military Medical University, Shanghai 200433, China.

**Keywords:** Renal cell carcinoma, Sunitinib, Glutaminyl peptide cyclotransferase, DNA methylation, HRAS

## Abstract

**Rationale**: Although sunitinib has been shown to improve the survival rate of advanced renal cell carcinoma (RCC) patients, poor drug response is a major challenge that reduces patient benefit. It is important to elucidate the underlying mechanism so that the therapeutic response to sunitinib can be restored.

**Methods**: We used an Illumina HumanMethylation 850K microarray to find methylation-differentiated CpG sites between sunitinib-nonresponsive and -responsive RCC tissues and Sequenom MassARRAY methylation analysis to verify the methylation chip results. We verified glutaminyl peptide cyclotransferase (QPCT) expression in sunitinib-nonresponsive and -responsive RCC tissues via qRT-PCR, western blot and immunohistochemical assays. Then, cell counting kit 8 (CCK-8), plate colony formation and flow cytometric assays were used to verify the function of QPCT in RCC sunitinib resistance after QPCT intervention or overexpression. Chromatin immunoprecipitation (ChIP) was performed to clarify the upstream regulatory mechanism of QPCT. A human proteome microarray assay was used to identify downstream proteins that interact with QPCT, and co-immunoprecipitation (co-IP) and confocal laser microscopy were used to verify the protein chip results.

**Results**: We found that the degree of methylation in the QPCT promoter region was significantly different between sunitinib-nonresponsive and -responsive RCC tissues. In the sunitinib-nonresponsive tissues, the degree of methylation in the QPCT promoter region was significantly reduced, and the expression of QPCT was upregulated, which correlated with a clinically poor response to sunitinib. A knockdown of QPCT conferred sunitinib sensitivity traits to RCC cells, whereas an overexpression of QPCT restored sunitinib resistance in RCC cells. Mechanistically, reducing the methylation degree of the QPCT promoter region by 5-aza-2'-deoxycytidine (decitabine) in RCC cells could increase the expression of QPCT and NF-κB (p65) bound to the QPCT promoter region, positively regulating its expression, while the hypermethylation in the QPCT promoter region could inhibit the binding of NF-κB (p65). QPCT could bind to HRAS and attenuate the ubiquitination of HRAS, thus increasing its stability and leading to the activation of the ERK pathway in RCC cells.

**Conclusion**: QPCT may be a novel predictor of the response to sunitinib therapy in RCC patients and a potential therapeutic target.

## Background

Renal cell carcinoma (RCC) is the most prevalent adult kidney malignancy, accounting for 2-3% of adult malignancies 1, and its incidence has been increasing in recent decades 2. RCC is associated with high rates of mortality and resistance to chemotherapy and radiotherapy 3. Treatments such as interleukin 2 and interferon alpha have no significant clinical effect, with less than 20% of patients responding well, and a median survival of 13.3 months; moreover, due to serious side effects, the patient's quality of life is seriously affected 4, 5. Patients with early-stage disease can be treated with surgical resection, but approximately 20-30% of patients present with metastatic disease at the initial diagnosis 6-8. Moreover, up to 20% of RCC patients suffer from metastatic lesions even if nephrectomy is performed^7^. Sunitinib is an oral multitarget receptor tyrosine kinase (RTK) inhibitor that has potent anti-angiogenic effects and direct anti-tumour activities 9, 10 due to the inhibition of vascular endothelial growth factor receptor (VEGFR), platelet-derived growth factor receptor, stem cell growth factor receptor, and FMS-like tyrosine kinase 3 11. Sunitinib has greatly improved the treatment prospects of advanced RCC, with a progression-free survival (PFS) twice that of patients receiving cytokine therapy 5, 12. However, approximately 20% of advanced RCC patients are inherently refractory to sunitinib therapy, and most of the remaining patients end up with drug resistance and tumour progression after 6-11 months of therapy 13, 14, resulting in the failure of sunitinib to efficiently prolong the survival of RCC patients. Several studies have proposed that the activation of compensatory signalling pathways cause the acquisition of sunitinib resistance, but the resistance mechanism remains unclear. In addition, few prognostic factors have been validated as predictive biomarkers of sunitinib response. Thus, it is urgent to elucidate the underlying mechanisms of sunitinib resistance and discover reliable biomarkers that can predict sunitinib response in RCC patients.

Epigenetics involves changes in gene expression that are not involved in changes in the DNA sequence and can be stably inherited during development and cell proliferation. DNA methylation is an important means of epigenetic modification. It is catalysed by DNA methyltransferase (DNMT), where S-adenosylmethionine (SAM) acts as a methyl donor, adding a methyl group to the 5-carbon of the cytosine ring and converting it to 5-methylcytosine (mC), which often occurs in CpG islands. CpG island methylation changes are involved in the regulation of gene expression and affect the structure of chromosomes. CpG islands are generally located in the promoter region of genes and contain binding sites for many transcription factors, and the methylation of this region prevents certain transcription factors from binding to them 15-20. Studies have found that the DNA methylation changes in several genes, such as RASSF1A 21-23, VHL 24-26, and EZH2 27, are associated with RCC and that DNA methylation changes could regulate the expression of these genes, ultimately leading to the development of RCC. However, there is still a lack of research on the role of DNA methylation regarding sunitinib resistance in RCC.

## Materials and Methods

### RCC patients and clinical samples

RCC patients who underwent surgical resection before adjuvant therapy in the Changhai Hospital or Changzheng Hospital (Shanghai, China) from 2006 to 2017 were included in this study. The clinical samples were stored in the Biobank of Shanghai Changzheng Hospital. Four pairs of sunitinib-responsive and nonresponsive RCC tissues were used for the Illumina Human Methylation 850K Microarray, and the detailed clinical characteristics of these patients are provided in Supplementary Table 1. Ten pairs of sunitinib-responsive and nonresponsive RCC tissues were used for Sequenom MassARRAY Methylation, and the detailed clinical characteristics of these patients are provided in Supplementary Table 2. The expression of QPCT, IRS1, SKI, PTK2B and NF-κB (p65) mRNA was detected in 16 pairs of sunitinib-responsive and nonresponsive RCC tissues. The detailed clinical characteristics of these patients are provided in Supplementary Table 3. The expression of QPCT, IRS1 and NF-κB (p65) protein was detected in 15 pairs of sunitinib-responsive and nonresponsive RCC tissues. The detailed clinical characteristics of these patients are provided in Supplementary Table 4. To evaluate the correlation between the QPCT level and sunitinib response, tumour tissues were collected from the biopsies or surgical specimens of 156 advanced clear cell RCC (ccRCC) patients between August 2006 and January 2017. These patients had not received systemic treatment before biopsy or radical nephrectomy. Patients in the sunitinib group (n=86) received at least two cycles of sorafenib therapy, and patients in the control group (n=70) received no therapy. These tissues were constructed into a tissue microarray (Biochip Company Ltd., China), and the QPCT level was determined by immunohistochemistry. The detailed clinical characteristics of these patients are listed in Supplementary Tables 5 and 8. The response to sunitinib in the RCC patients was determined by computed tomography (CT) or magnetic resonance imaging (MRI), clinical progression or death, and the use of the Response Evaluation Criteria in Solid Tumors (RECIST). To study the content of QPCT in peripheral blood, we selected plasma samples of sunitinib-responsive and nonresponsive patients from the Changhai Hospital and Changzheng Hospital. The detailed clinical characteristics of these patients are listed in Supplementary Tables 6 and 7.

### Illumina HumanMethylation 850K microarray

Illumina HumanMethylation 850K microarray profiling and data analysis were performed by Oebiotech (Shanghai).

### Sequenom MassARRAY methylation

Sequenom MassARRAY methylation profiling and data analysis were performed by CapitalBio Technology Corporation (Beijing).

### Cell Lines and reagents

The human RCC cell lines (OS-RC-2, Caki-2, Caki-1, A498, 786-O, ACHN, 769-P, and KETR-3) and HK-2 cells were obtained from the Chinese Academy of Sciences (Shanghai, China). A498 and ACHN cells were incubated in Minimum Essential Medium (MEM) (10-010-CV, Corning, USA) supplemented with 10% foetal bovine serum (FBS, 16000044, Gibco, USA), and the other cells were incubated in Roswell Park Memorial Institute (RPMI) 1640 (10-040-CV, Corning, USA) containing 10% FBS. Cells were grown as a monolayer on plastic cell culture dishes at 37°C in a humidified atmosphere containing 5% CO_2_. Sunitinib, decitabine, triptolide and SCH772984 were purchased from Selleck Chemicals (China). MG132 and cycloheximide (CHX) were purchased from APExBIO (USA). Lonafarnib and betulinic acid (BetA) were purchased from TargetMol USA.

### Animal studies

The animal studies were approved by the Institutional Animal Care and Use Committee of the Second Military Medical University, Shanghai, China. Male athymic BALB/c nude mice (4 weeks old) were used. A total of 5×10^6^ lv-QPCT and lv-NC 786-O cells were injected subcutaneously into the left and right sides of the mice (n=6). When the xenografts grew to 100 mm^3^, the mice were treated with either saline (control) or sunitinib (40 mg/kg/day). Xenograft volumes were evaluated by calliper measurements of two perpendicular diameters and calculated individually with the following formula: Volume = a×b^2^/2 (where a represents the length and b represents the width). Xenograft samples were collected for histological evaluation (paraffin section) or were snap-frozen in liquid nitrogen.

### RNA extraction, cDNA preparation and qRT-PCR

Total RNA was extracted from cells and tissues using TRIzol reagent (Takara, Japan), according to the manufacturer's instructions. Total RNA quality was assessed using a Nanodrop 2000 and agarose gel electrophoresis. First-strand cDNA was generated from 2 µg of total RNA using M-MLV reverse transcriptase (Invitrogen, CA) with random primers. qRT-PCR was performed according to the SYBR Green protocol in a Step One Plus System (Applied Biosystems, Foster City, CA, USA), and β-actin served as the endogenous control. The primer sequences used were as follows: QPCT, 5'-AAATTGCAGAAGGCACCAGT-3' (forward) and 5'-CTGAATTCGCTGCATGATGT-3' (reverse); SKI, 5'-TCCTCCTTGTCCTCGCTCTC-3' (forward) and 5'-TTGGCTTCCTTGGTGTCCAG-3' (reverse); PTK2B, 5'-GTGGGAGATCCTGAGCTTTG-3' (forward) and 5'-TAAAGGACCGGTGGACAGAG-3' (reverse); IRS1, 5'-TTGAGAATGTGTGGCTGAGG-3' (forward) and 5'-TCCTTGACCAAATCCAGGTC-3' (reverse); NF-κB, (p65) 5'-AGGCTCCTGTGCGTGTCTCC-3' (forward) and 5'-TCGTCTGTATCTGGCAGGTACTGG-3' (reverse); and β-actin, 5'-CTGGTGCCTGGGGCG-3' (forward) and 5'-AGCCTCGCCTTTGCCGA-3' (reverse). Relative mRNA expression levels were calculated based on the corresponding relative quantitation (RQ) values and were normalized to β-actin expression.

### Western blot analysis

Total cell and tissue lysates were prepared in 1× sodium dodecyl sulphate (SDS) buffer. Identical quantities of protein were separated by SDS gel electrophoresis and transferred onto nitrocellulose filter membranes. After incubating with antibodies specific for QPCT (ab201172, Abcam, CA, USA) and GAPDH (sc-25778; Santa Cruz, CA, USA), the blots were incubated with IRDye 800-conjugated goat anti-rabbit IgG, and bands were detected using an Odyssey infrared scanner (Li-Cor). GAPDH was used as the loading control. The other antibodies used for western blot were against IRS1 (ab52167, Abcam, CA, USA), NF-κB (p65) (8242, Cell Signalling Technology), HRAS (ab32417, Abcam, CA, USA), CBL (ab32027, Abcam, CA, USA), GAB1 (ab59362, Abcam, CA, USA), NAF1 (ab157106, Abcam, CA, USA), MAPK8 (ab199380, Abcam, CA, USA), MAPK10 (ab126591, Abcam, CA, USA), FAK (PTK2) (ab40794, Abcam, CA, USA), p-FAK (ab81298, Abcam, CA, USA), ERK1/2 (4695, Cell Signaling Technology), p-ERK1/2 (4370, Cell Signaling Technology), AKT (4691, Cell Signaling Technology), p-AKT (4060, Cell Signaling Technology), Stat3 (9139, Cell Signaling Technology), p-Stat3 (9145, Cell Signaling Technology), and ubiquitin (3936, Cell Signaling Technology).

### siRNA transfection

QPCT siRNA was synthesized by GenePharma (Shanghai, China), with the following sequences: 5'-GCACCAGUAUCUCUGAAAUTT-3' (forward) and 5'-AUUUCAGAGAUACUGGUGCTT-3' (reverse); and 5'-CCUCAAUCCCACUGCUAAATT-3' (forward) and 5'-UUUAGCAGUGGGAUUGAGGTT-3' (reverse). A non-silencing siRNA oligonucleotide that does not recognize any known mammalian gene homologue (GenePharma, Shanghai, China) was used as the negative control, with the following sequence: 5'-UUCUCCGAACGUGUCACGUTT-3' (forward) and 5'-ACGUGACACGUUCGGAGAATT-3' (reverse). RCC cells were transfected with QPCT siRNA (50 nmol/L) or control siRNA (50 nmol/L) via LipofectamineTM RNAiMAX Transfection Reagent (InvitrogenTM) according to the manufacturer's instructions.

### Lentiviral packaging and transfection

Lentiviruses encoding human QPCT were constructed and produced by Obio Technology (Shanghai). 786-O and A498 cells were infected following the manufacturer's instructions. After 72 h, puromycin was added to obtain the stably transfected cell lines.

### Cell counting kit 8 (CCK-8) assay

RCC cells were cultured in different concentrations of sunitinib (0 μM, 2 μM, 4 μM, 8 μM, 10 μM, 16 μM, 20 μM, and 32 μM). Then, 100 µl of culture medium containing 10 µl of CCK-8 reagent (Dojindo Molecular Technologies, Inc., Kumamoto, Japan) was added to each well for another 2 h of incubation at 37°C. The absorbance was recorded at 450 nm using a microplate reader (Varioskan Flash; Thermo Scientific, Waltham, MA, USA). Viability (%) was calculated based on the optical density (OD) values. All experiments were independently repeated in triplicate on separate occasions.

### Plate colony formation assay

RCC cells (500 cells) were seeded into 6-well plates with sunitinib (5 μM) and cultured in a 37°C incubator for 10 days until most single colonies were composed of more than 50 cells. The plates were washed with phosphate-buffered saline (PBS), fixed with 4% paraformaldehyde, and stained with crystal violet. The number of colonies containing more than 50 cells was counted in each well.

### Flow cytometric analysis

Cell apoptosis was quantified using flow cytometric analysis (BD Biosciences, San Jose, CA). For apoptosis experiments, RCC cells cultured with sunitinib (5 μM) were collected and washed twice with ice-cold PBS and then re-suspended in 200 µl of binding buffer. Fluorescein isothiocyanate (FITC)-conjugated Annexin V was added at a final concentration of 0.5 µg/ml and incubated for 20 minutes at room temperature in the dark; then, 1 µg/ml propidium iodide (PI) was added. The samples were immediately analysed by flow cytometry.

### Chromatin immunoprecipitation (ChIP)

We performed ChIP using an EZ ChIP Chromatin Immunoprecipitation Kit for cell line samples (Millipore) according to the manufacturer's instructions. The sequences for Primer1 (containing an NF-κB binding QPCT site) were as follows: 5'-CGTTTGTGGTGGATACAGGAG-3' (forward) and 5'- TTCCAGCCAAAAGAGCTTGAC-3' (reverse). An anti-NF-κB (p65) (8242, Cell Signaling Technology, 1:100) antibody was used for ChIP.

### Plasmid construction

The full-length HRAS mRNA sequence was obtained from the National Center for Biotechnology Information (NCBI) website (NM_005343). The fragment was obtained by gene synthesis and cloned into a pcDNA3.1 vector (Hanbio Biotechnology Co., Ltd., Shanghai).

### Human proteome microarray assay

The HuProt microarray assay 28, 29 and data analysis were performed by Wayen Biotechnologies Inc., Shanghai according to the following procedure. The HuProt microarray (CDI Laboratories, Inc.) comprises 20,240 human full-length proteins with N-terminal glutathione S-transferase (GST) tags. Human proteome microarrays (HuProtTM 20 K) were blocked with blocking buffer (1% BSA and 0.1% Tween 20 in TBST) for 1 h at room temperature with gentle agitation. The QPCT protein was labelled with biotin with an Antibody Array Assay Kit (Full Moon Biosystems, Sunnyvale, CA) and was then diluted to 0.01 mg/ml in blocking buffer and incubated on the blocked proteome microarray at room temperature for 1 h. The microarrays were washed three times for 5 min each with TBST, incubated with streptavidin-Cy5 at a dilution of 1:1000 (Thermo Fisher Scientific, USA) for 1 h at room temperature and subjected to three more 5-min washes. The microarrays were spun dry at 1500 rpm for 3 min and subjected to scanning with a GenePix 4000B (Axon Instruments, Sunnyvale, CA) to visualize and record the results. GenePix Pro 6.0 was used for data analysis. The information of all proteins contained in the HuProt microarray in the Supplementary Table 9 and the information of proteins that may bind to QPCT was provided in the Supplementary Table 10.

### Co-immunoprecipitation

Co-immunoprecipitation (co-IP) was performed according to the manufacturer's instructions (Pierce Co-Immunoprecipitation Kit, Thermo Scientific). Antibodies against QPCT (sc-517122, Santa Cruz, 1:50) and HRAS (ab32417, Abcam, 1:50) were used.

### Immunocytochemistry

RCC cells were plated in special laser confocal culture dishes at 30% confluence and treated with reagents at different concentrations for 48 h. Then, the cells were fixed with a 4% paraformaldehyde solution for 15 minutes at room temperature, permeabilized with 0.4% Triton X-100 in PBS for 5 minutes, and then blocked with 1% BSA in PBS for 1 h at 37°C. The blocked cells were incubated with anti-QPCT antibody (PA5-76997, ThermoFisher, 1:50) and anti-HRAS antibody (LS-C340614/132294, LifeSpan BioSciences, Inc. 1:100) overnight at 4°C, followed by incubation with secondary antibody (appropriately respond to primary antibody in species) labelled with HRP, incubate at room temperature for for 2 h. The nuclear staining of cells was conducted using 4,6-diamidino-2-phenylindole (DAPI). Representative images were acquired using the Leica Microsystem. DAPI glows blue by UV excitation wavelength 330-380 nm and emission wavelength 420 nm; FITC glows green by excitation wavelength 465-495 nm and emission wavelength 515-555 nm; CY3 glows red by excitation wavelength 510-560 nm and emission wavelength 590 nm. Nucleus is blue by labeling with DAPI, HRAS is green by labeling with FITC and QPCT is red by labeling with CY3. After merging under the laser confocal microscope, the yellow fluorescence indicates co-localization of HRAS and QPCT.

### Immunohistochemistry

Specimens were stained with antibodies for QPCT (ab201172, 1:100) or HRAS (ab97488, 1:100). The sections were heated at 70°C for 1 h, dewaxed in xylene, and dehydrated through a gradient concentration of alcohol. After retrieving and blocking endogenous peroxidase and nonspecific staining with 3% H2O2 and normal bovine serum, the sections were incubated with primary antibody overnight at 4°C. The slides were then incubated with horseradish peroxidase (HRP)-conjugated secondary antibody for 10 minutes at 37°C. Finally, the sections were visualized by diaminobenzidine (DAB) solution and counterstained with haematoxylin. Two pathologists blinded to the patient outcome independently scored the staining intensities and percentages of positive tumour cells.

### Data analysis

All statistical analyses in this study were performed with SPSS 22.0 software (SPSS Inc, USA). Data are presented as the means±sd. The significance of the differences between the mean values of two groups was analysed by a two-tailed Student's t-test. Spearman correlation analysis was performed to determine the correlation between two variables. The Pearson chi-square test was used to analyse the clinical variables. Kaplan-Meier survival analysis was utilized to compare RCC patient survival based on dichotomized QPCT expression by a log-rank test. A p-value of 0.05 was considered significant.

## Results

### Methylation levels in the QPCT promoter region were reduced and QPCT expression was increased in the sunitinib-nonresponsive tissues of RCC

**Figure 1:**
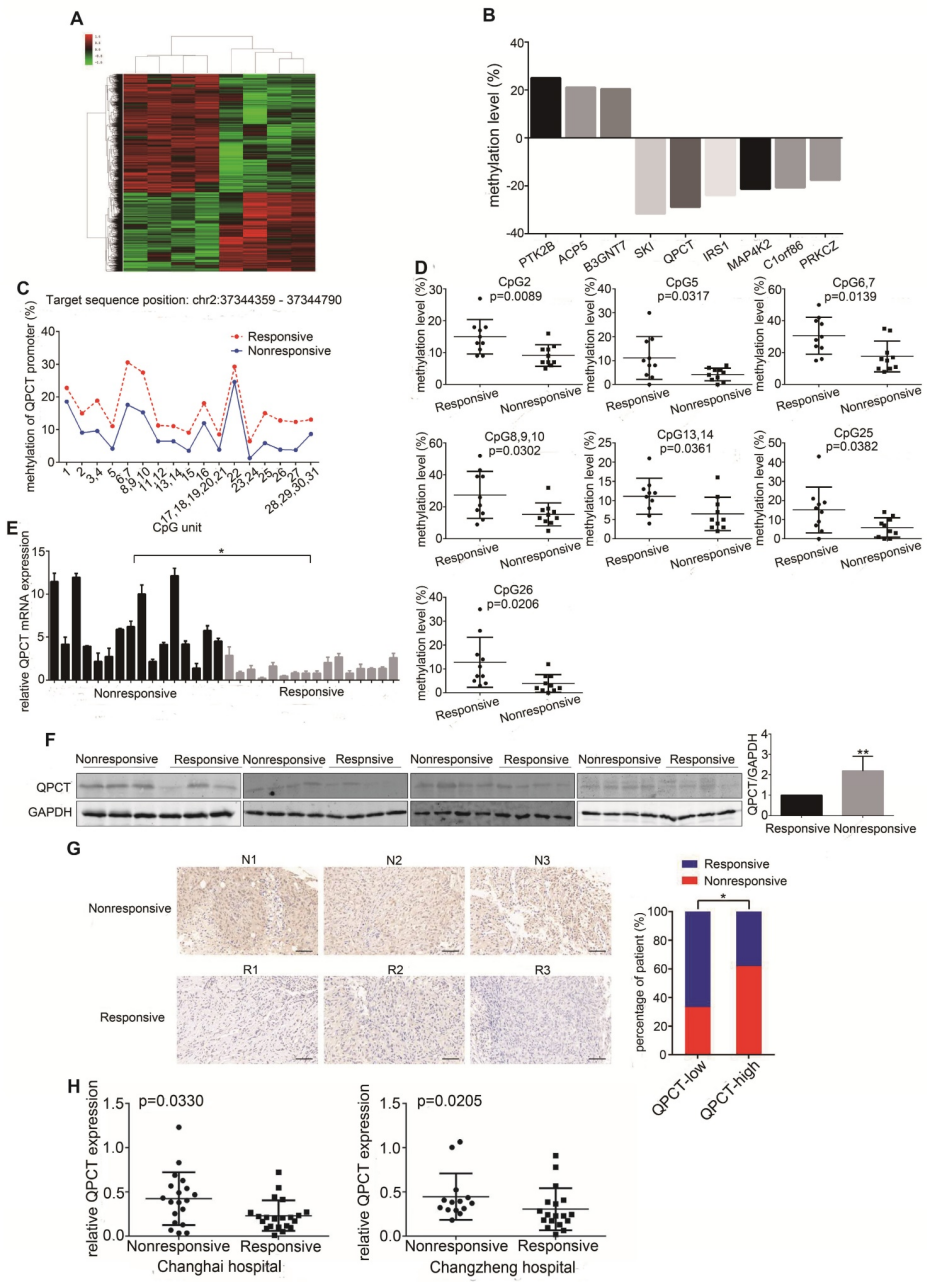
** Methylation levels in the QPCT promoter region were reduced and QPCT expression was increased in the sunitinib-nonresponsive tissues of RCC.** (A) Cluster analysis of the Illumina Human Methylation 850K Microarray in 4 pairs of sunitinib-responsive and nonresponsive RCC tissues (heat map). (B) The nine target genes screened by the Illumina Human Methylation 850K Microarray (a positive value indicates that the methylation levels of the sunitinib-nonresponsive group were higher than those of the sunitinib-responsive group of RCC; a negative value indicates that the methylation levels of the sunitinib-nonresponsive group were lower than those of the sunitinib-responsive group of RCC). (C) Methylation levels of QPCT in 10 pairs of sunitinib-responsive and nonresponsive RCC tissues by Sequenom MassARRAY Methylation. (D) CpG sites that had differences between the two groups in the QPCT promoter regions. (E) Expression of QPCT mRNA in 16 pairs of sunitinib-responsive and nonresponsive RCC tissues. (F) Western blot analysis of QPCT protein in 15 pairs of sunitinib-responsive and nonresponsive RCC tissues. (G) Representative immunohistochemical results of QPCT expression in the sunitinib-nonresponsive and sunitinib-responsive tissues of RCC, scale bar, 100 μm (left) and percentages of samples that were nonresponsive and responsive to sunitinib in different QPCT levels (right). (H) Elisa analysis of QPCT levels in the plasma of RCC patients who were nonresponsive or responsive to sunitinib from the Changhai Hospital and the Changzheng Hospital. The results are presented as the means ± SD. *p<0.05, **p < 0.01.

To detect the changes in DNA methylation responsible for sunitinib resistance, we performed an Illumina Human Methylation 850K Microarray analysis in a set of pre-treated tumour tissues from a cohort of RCC patients who presented with good or poor responses to sunitinib therapy (Figure 1A, Supplementary Table 1). According to the diffscore and delta-beta values (diffscore was less than -13 or greater than 13, and the delta-beta value was greater than 0.17 or less than -0.17), as well as the methylation difference sites located in the promoter region, 9 genes were screened out (Figure 1B). Then, another independent set of pre-treated RCC tissues with a distinct response to sunitinib was used to verify the microarray results by Sequenom MassARRAY Methylation. The methylation levels of 4 gene promoter regions were confirmed to have statistically significant differences (Figure 1C and 1D, Supplementary Figure 1A and B, Supplementary Table 2). We further validated the expression of 4 genes in the set of pre-treated RCC tissues at the mRNA level, and the expression of QPCT and IRS1, but not SKI and PTK2B, was significantly higher in the sunitinib-nonresponsive group than in the responsive group (Figure 1E, Supplementary Figure 1C, Supplementary Table 3). We further detected QPCT and IRS1 protein in the set of pre-treated RCC tissues. We found that the expression of QPCT, but not IRS1, was significantly higher in the sunitinib-nonresponsive group than in the responsive group (Figure 1F, Supplementary Figure 1D, Supplementary Table 4). Then, we used a previously established tissue microarray that included 156 RCC tissue samples to detect the expression of QPCT by immunohistochemistry, confirming that QPCT was upregulated in the sunitinib-nonresponsive tissues of RCC (Figure 1G, Supplementary Table 5). Since QPCT can be secreted by tumour cells, we detected the content of QPCT in plasma in the set of pre-treated RCC patients who presented with good or poor responses to sunitinib therapy.

We found that the QPCT level was higher in the plasma of sunitinib-nonresponsive patients than that in sunitinib-responsive patients (Figure 1H, Supplementary Tables 6 and 7). Then, we examined the expression of QPCT in RCC cells, which was higher than that in control HK-2 cells (Supplementary Figure 1E).

Currently, there is no literature on the role of QPCT or its DNA methylation changes in the resistance of RCC to sunitinib. Thus, we focused on QPCT to discover new knowledge regarding sunitinib resistance in RCC.

### Downregulation of QPCT could promote sensitivity of RCC cells to sunitinib

**Figure 2:**
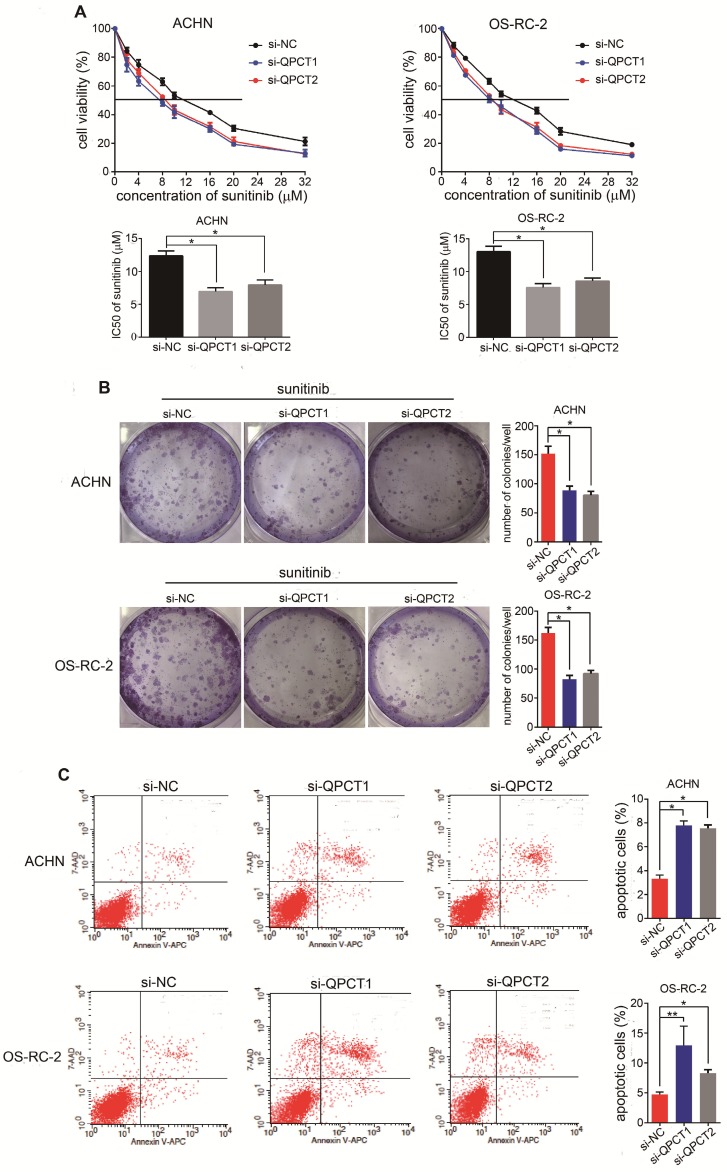
** Downregulation of QPCT could promote the sensitivity of RCC cells to sunitinib.** (A) CCK-8 assay of ACHN and OS-RC-2 cells transfected with si-QPCT1, si-QPCT2 or si-NC after sunitinib treatment at the indicated concentrations for 48 h (n=3). The IC50 values are shown in the bottom histogram. (B) Cell clone formation experiments of ACHN and OS-RC-2 cells transfected with si-QPCT1, si-QPCT2 or si-NC after sunitinib (5 μM) treatment for 10 days (n=3). Representative images (left) and average number of RCC colonies (right) are shown. (C) Flow cytometry analysis of Annexin V-stained ACHN and OS-RC-2 cells transfected with si-QPCT1, si-QPCT2 or si-NC after sunitinib treatment (5 μM) for 48 h (n=3). Representative images (left) and average number of apoptotic cells (right) are shown. Results are presented as the means ± SD. *p<0.05, **p < 0.01.

To thoroughly explore the function of QPCT in sunitinib resistance, we suppressed QPCT expression utilizing two small interfering RNAs (siRNAs) against QPCT in the ACHN and OS-RC-2 cell lines (Supplementary Figure 2A). Compared to the response of the control group, silencing QPCT led to decreased half maximal inhibitory concentration (IC50) values in RCC cells (Figure 2A) as well as a decreased capacity for cloning when exposed to sunitinib (Figure 2B). Consistently, flow cytometry showed that an exposure to sunitinib resulted in an increased proportion of apoptotic cells among QPCT-knockdown RCC cells (Figure 2C). Together, these data indicate that QPCT was required for sunitinib resistance *in vitro*.

### Overexpression of QPCT could promote sunitinib resistance in RCC *in vitro* and *in vivo*

**Figure 3:**
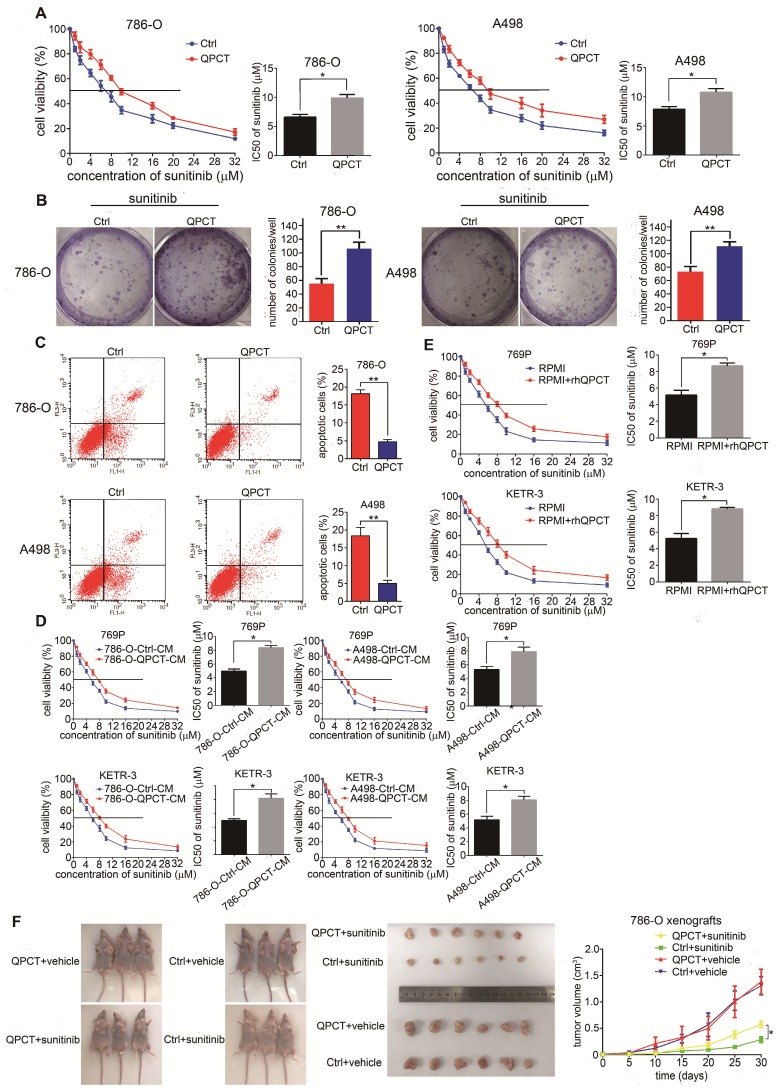
** Overexpression of QPCT could promote sunitinib resistance in RCC *in vitro* and *in vivo*.** (A) CCK-8 assay of QPCT-overexpressing and control 786-O and A498 cells after sunitinib treatment at the indicated concentrations for 48 h (n=3). The IC50 values are shown in the right histogram. (B) Cell clone formation experiments of QPCT-overexpressing and control 786-O and A498 cells after sunitinib (5 μM) treatment for 10 days (n=3). Representative images (left) and average number of RCC colonies (right) are shown. (C) Flow cytometry analysis of Annexin V-stained QPCT-overexpressing and control 786-O and A498 cells after sunitinib treatment (5 μM) for 48 h (n=3). Representative images (left) and average number of apoptotic cells (right) are shown. (D) CCK-8 assay of 769-P and KETR-3 cultured with the supernatants of QPCT-overexpressing 786-O and A498 cells and control 769-P and KETR-3 cells after sunitinib treatment at the indicated concentrations for 48 h (n=3). The IC50 values are shown in the right histogram. (E) CCK-8 assay of 769-P and KETR-3 cultured with purified QPCT cytokine (10 μM) and control 769-P and KETR-3 cells after sunitinib treatment at the indicated concentrations for 48 h (n=3). The IC50 values are shown in the right histogram. (F) Subcutaneous xenograft growth in nude mice under different treatment conditions (left), anatomical picture of subcutaneous xenografts in nude mice (middle), and growth curve of subcutaneous xenografts (right) are shown. Results are presented as the means ± SD. *p<0.05, **p<0.01.

Next, we overexpressed QPCT in the 786-O and A498 cell lines (Supplementary Figure 3A). Compared with the control group, RCC cells overexpressing QPCT displayed an increased tolerance to sunitinib treatment and led to increased IC50 values (Figure 3A). Meanwhile, the capacity for cloning was also enhanced when exposed to sunitinib (Figure 3B). Flow cytometry also showed that QPCT overexpression attenuated sunitinib-induced cell apoptosis (Figure 3C).

By adding the culture supernatant from RCC cells stably overexpressing QPCT or adding purified QPCT cytokines (rhQPCT) into the culture medium, we found that the RCC cells cultured in the conditioned medium were more resistant to sunitinib than control cells (Figure 3D and E).

Then, we injected QPCT-overexpressing and control 786-O cells subcutaneously into the left and right axils of nude mice. When the volume of the xenograft reached 100 mm^3^, the mice were orally treated with vehicle or sunitinib (40 mg/kg/day). The results showed that the xenografts formed from QPCT-overexpressing RCC cells exhibited worse responses to sunitinib (Figure 3F).

Collectively, these findings indicate that the overexpression of QPCT endowed RCC cells with refractoriness to sunitinib.

### Reducing the methylation levels of the QPCT promoter region by decitabine in RCC cells could increase the expression of QPCT and NF-κB (p65) bound to the QPCT promoter region, positively regulating its expression

**Figure 4:**
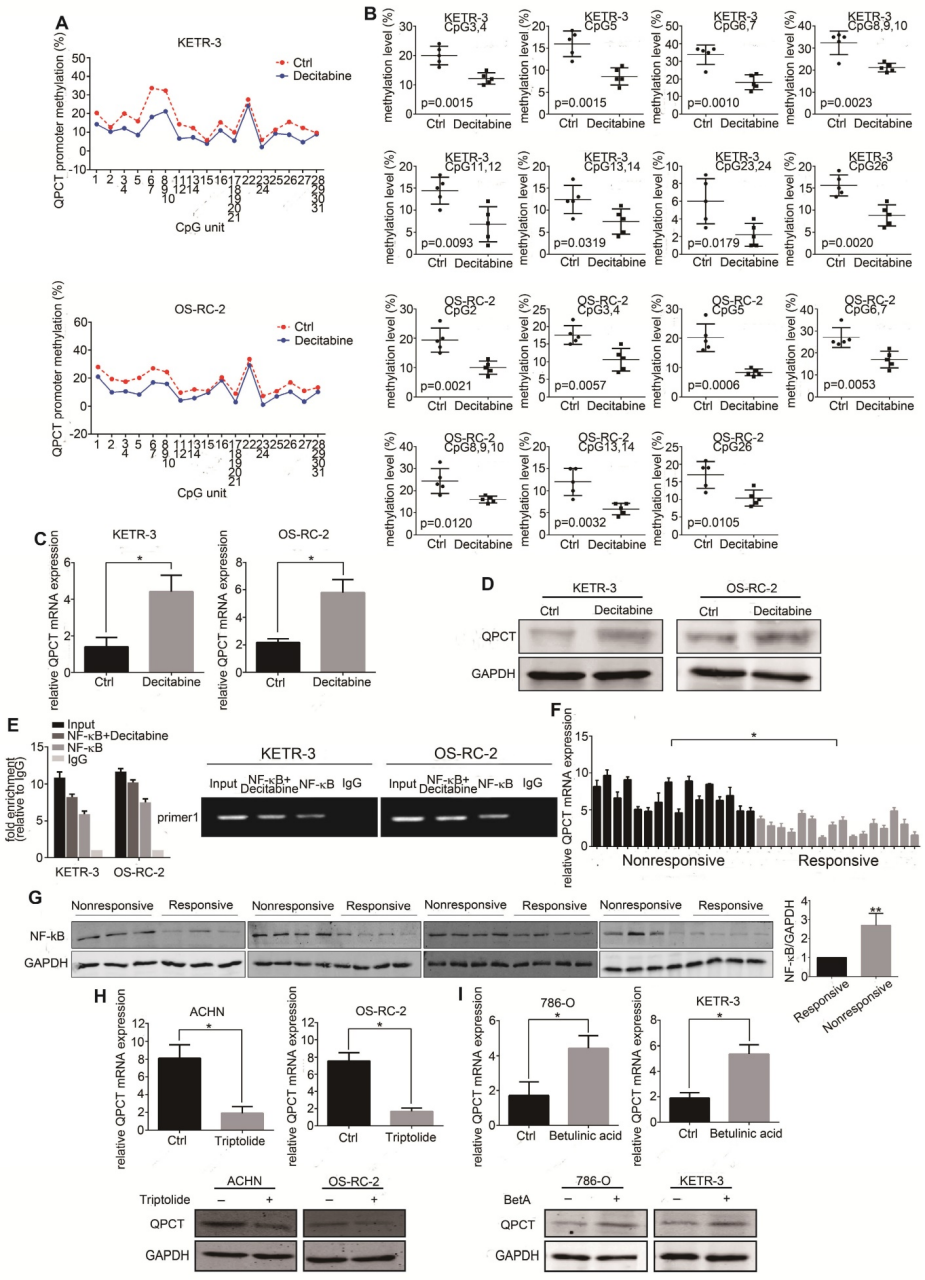
** Reducing the methylation levels of the QPCT promoter region by decitabine in RCC cells could increase the expression of QPCT and NF-κB (p65) bound to the QPCT promoter region, positively regulating its expression.** (A) Sequenom MassARRAY Methylation of the QPCT promoter region in KETR-3 and OS-RC-2 cells after decitabine (0.5 μM) treatment for 48 h and control KETR-3 and OS-RC-2 cells (n=5). (B) CpG sites that had differences between the two groups in the QPCT promoter region (n=5). (C) QPCT mRNA expression in KETR-3 and OS-RC-2 cells after decitabine (0.5 μM) treatment and control KETR-3 and OS-RC-2 cells (n=3). (D) QPCT protein in KETR-3 and OS-RC-2 cells after decitabine (0.5 μM) treatment and control KETR-3 and OS-RC-2 cells (n=3). (E) qPCR analysis of NF-κB (p65) mRNA in 16 pairs of sunitinib-responsive and nonresponsive RCC tissues. (F) Western blot analysis of NF-κB (p65) protein in 15 pairs of sunitinib-responsive and nonresponsive RCC tissues. (G) ChIP analysis demonstrated that NF-κB (p65) binds to the QPCT promoter region and increased NF-κB (p65) binding to the QPCT promoter after inhibiting the methylation levels of QPCT. (H) QPCT mRNA (above) and protein (below) expression in ACHN and OS-RC-2 cells after triptolide (10 nM) treatment for 72 h and control ACHN and OS-RC-2 cells (n=3). (I) QPCT mRNA (above) and protein (below) expression in 786-O and KETR-3 cells after BetA (5 μM) treatment for 72 h and control 786-O and KETR-3 cells (n=3). Results are presented as the means ± SD. *p<0.05, **p<0.01.

To determine whether methylation changes affected its expression, we treated the RCC cell lines with decitabine and detected a decrease in methylation in the QPCT promoter region by Sequenom MassARRAY Methylation (Figure 4A and B). The expression of QPCT was upregulated by qPCR (Figure 4C) and western blot (Figure 4D).

According to the related literature, NF-κB may be a transcription factor regulating the expression of QPCT 30. Through a ChIP assay, we demonstrated that NF-κB (p65) could directly bind to the QPCT promoter region and that the possible binding site of NF-κB (p65) was located in the -2042 bp of the ATG transcriptional start codon. We also found that after reducing the methylation levels of the QPCT promoter region by decitabine in RCC cells, the binding of NF-κB (p65) to QPCT was increased (Figure 4E), which indicated that a hypermethylation of the QPCT promoter region might inhibit the binding of NF-κB (p65). We found that NF-κB (p65) was upregulated in sunitinib-nonresponsive RCC tissues at both the mRNA (Figure 4F) and protein levels (Figure 4G). In addition, the inhibition of NF-κB (p65) by triptolide 31-33 or the activation of NF-κB (p65) by BetA 34 could prevent or promote the expression of QPCT (Figure 4H and I), respectively, which is consistent with the findings of previous reports.

The above results indicate that the methylation changes in the QPCT promoter region were synergistic with NF-κB (p65) in the transcriptional regulation of QPCT. Hypermethylation inhibited the binding of NF-κB (p65) to QPCT and suppressed the expression of QPCT, while hypomethylation facilitated the binding of NF-kB and promoted the expression of QPCT.

### QPCT could bind with HRAS and promote the stability of HRAS by reducing its ubiquitination degradation

To illuminate the mechanism underlying the role of QPCT in sunitinib resistance in RCC, we used a human proteome microarray composed of 20,240 full-length human proteins with N-terminal glutathione S-transferase (GST) tags to find QPCT-interacting proteins. A total of 366 proteins that might interact with QPCT were detected. The subcellular localization of QPCT was measured, and QPCT was not only secreted outside RCC cells but also widely distributed in the cytoplasm and nucleus of RCC cells (Supplementary Figure 5A and B).

**Figure 5:**
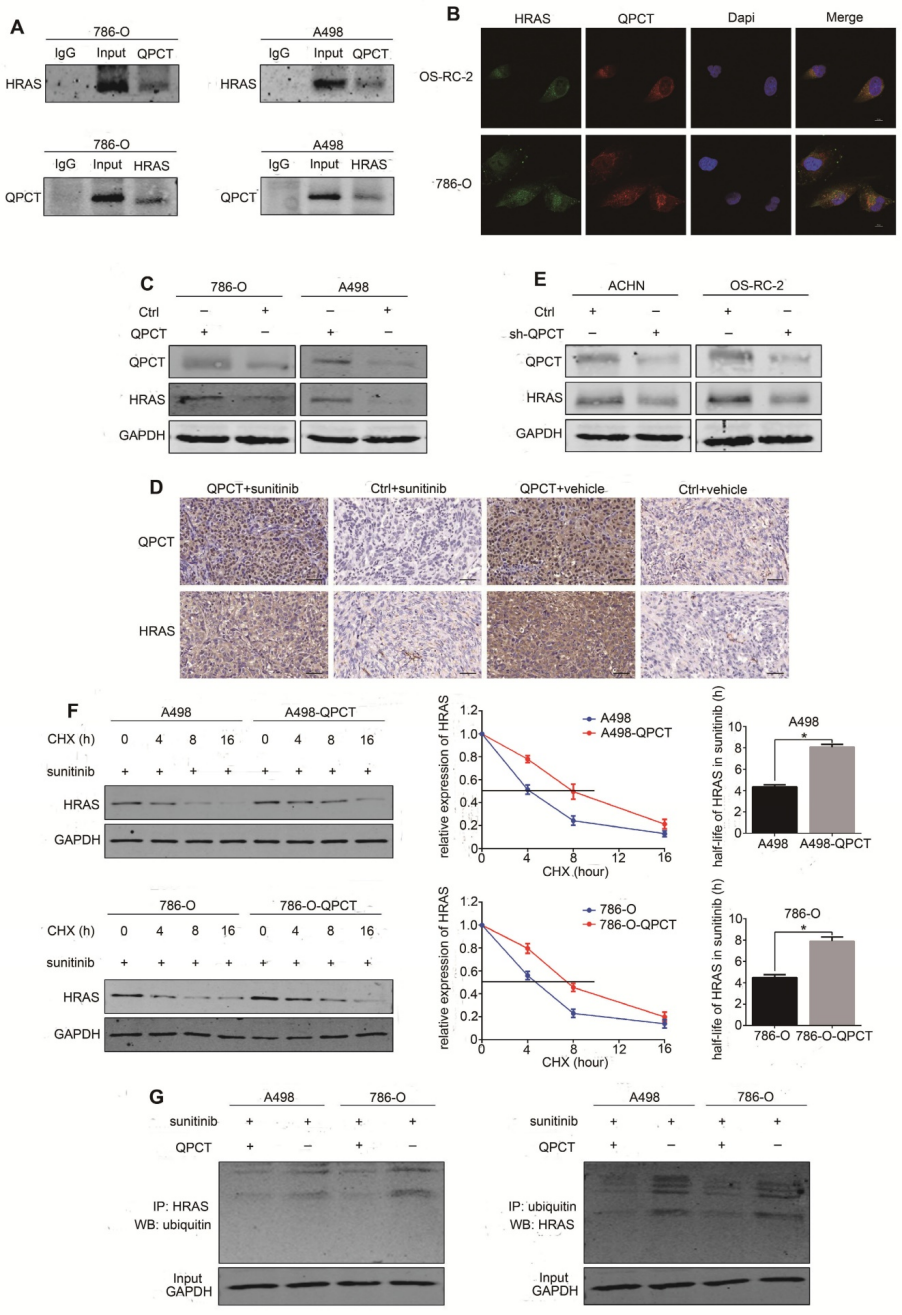
** QPCT could bind with HRAS and promote the stability of HRAS by reducing its ubiquitination degradation.** (A) Co-immunoprecipitation of QPCT and HRAS in 786-O and A498 cells. (B) Immunofluorescence analysis of QPCT (red) and HRAS (green) in OS-RC-2 and 786-O cells. Scale bar, 10 μm. (C) Representative images of western blot analysis of QPCT and HRAS in QPCT-overexpressing and control 786-O and A498 cells. (D) Immunohistochemistry of QPCT and HRAS in xenografts. Scale bar, 100 μm. (E) Representative images of western blot analysis of QPCT and HRAS in ACHN and OS-RC-2 cells transfected with sh-QPCT or sh-NC. (F) Western blot analysis of HRAS in QPCT-overexpressing and control 786-O and A498 cells after cycloheximide (CHX) and sunitinib (5 μM) treatment for various times. (G) Western blot analysis of HRAS ubiquitination in QPCT-overexpressing and control 786-O and A498 cells after sunitinib (5 μM) treatment for 48 h. GAPDH was used as a loading control. Results are presented as the means ± SD. *p<0.05, **p<0.01.

In the screening of protein chip results by Kyoto Encyclopedia of Genes and Genomes/Gene Ontology (KEGG/GO) analysis and analysis of the intracellular localization of the proteins, 7 target proteins were initially screened out: PTK2, HRAS, CBL, GAB1, NAF1, MAPK8, and MAPK10 (Supplementary Figure 5C). We verified the chip results by co-IP, and only HRAS was shown to be able to combine with QPCT (Figure 5A, Supplementary Figure 5D). Consistently, QPCT co-localized with HRAS in the cytoplasm by immunofluorescence staining and laser confocal microscopy observation (Figure 5B).

We found that the expression of HRAS was upregulated in RCC cells that stably overexpressed QPCT (Figure 5C), as well as in the xenograft tumours (Figure 5D), while the expression of HRAS was downregulated when QPCT was knocked down in RCC cells (Figure 5E, Supplementary Figure 5E). To further investigate whether QPCT could inhibit the degradation of HRAS, a cycloheximide (CHX) chase experiment was performed. The results of this experiment demonstrated that the overexpression of QPCT could increase the stability of HRAS (Figure 5F). Furthermore, the ubiquitination assay revealed that the overexpression of QPCT could reduce the sunitinib-induced ubiquitination of HRAS (Figure 5G).

### HRAS plays a role in QPCT-mediated sunitinib resistance by promoting ERK phosphorylation in RCC cells

**Figure 6:**
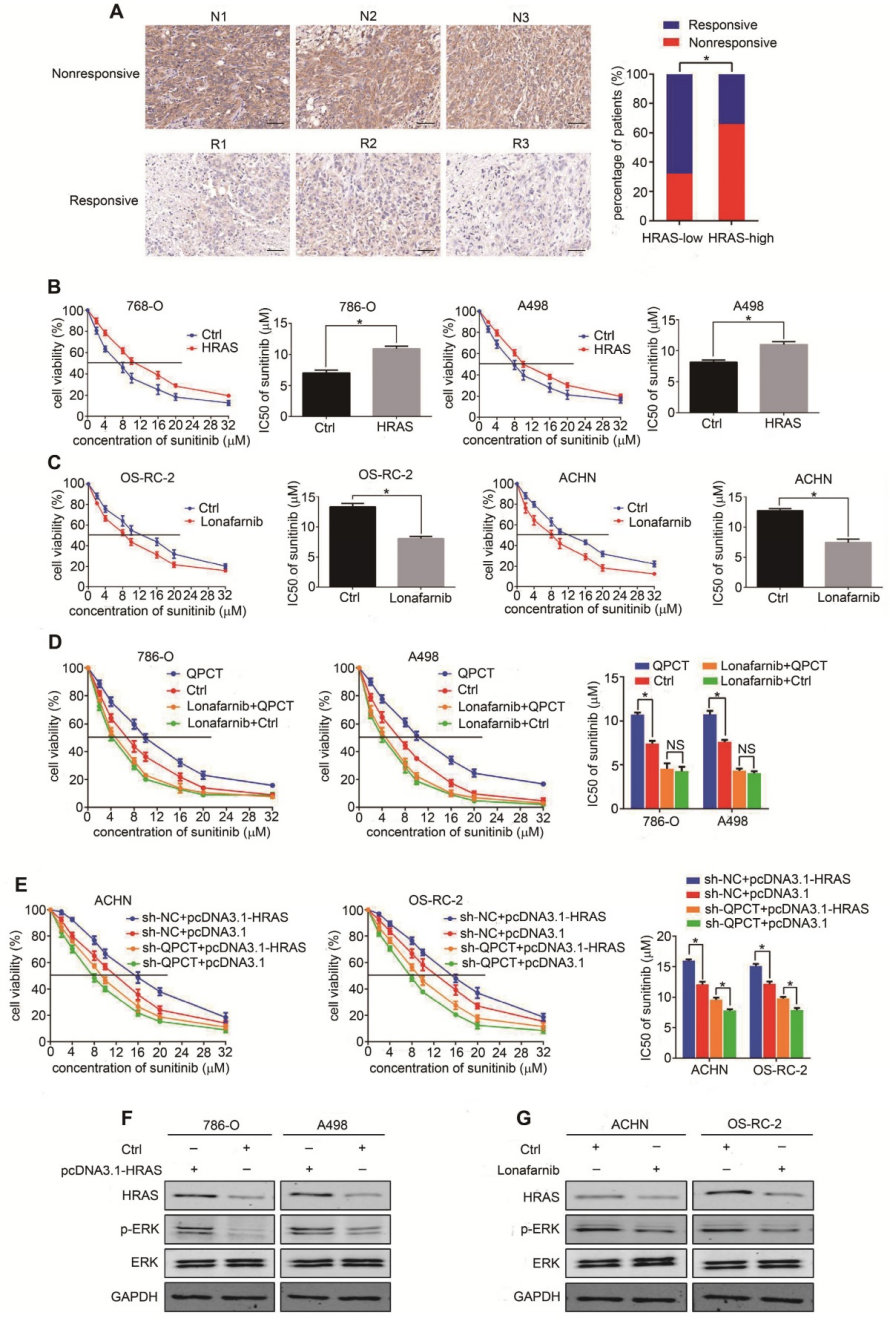
** HRAS plays a role in QPCT-mediated sunitinib resistance by promoting ERK phosphorylation in RCC cells.** (A) Representative immunohistochemical results of HRAS expression in the sunitinib-nonresponsive and sunitinib-responsive tissues of RCC. Scale bar, 100 μm (left) and percentages of samples that were nonresponsive and responsive to sunitinib in different HRAS levels (right). (B) CCK-8 assay of 786-O and A498 cells transfected with plasmid pcDNA3.1-HRAS or control plasmid after sunitinib treatment at the indicated concentrations for 48 h (n=3). The IC50 values are shown in the right histogram. (C) CCK-8 assay of OS-RC-2 and ACHN cells with lonafarnib (1.9 nM) treatment or control OS-RC-2 and ACHN cells after sunitinib treatment at the indicated concentrations for 48 h (n=3). The IC50 values are shown in the right histogram. (D) CCK-8 assay of QPCT-overexpressing and control 786-O and A498 cells treated with lonafarnib (1.9 nM) after sunitinib treatment at the indicated concentrations for 48 hours (n=3). The IC50 values are shown in the rightmost histogram. (E) CCK-8 assay of sh-QPCT or sh-NC ACHN and OS-RC-2 cells transfected with plasmid pcDNA3.1-HRAS or control plasmid after sunitinib treatment at the indicated concentrations for 48 hours (n=3). The IC50 values are shown in the rightmost histogram. (F) Western blot analysis of p-ERK in HRAS-overexpressing and control 786-O and A498 cells. (G) Western blot analysis of p-ERK in ACHN and OS-RC-2 cells treated with lonafarnib (1.9 nM) and control ACHN and OS-RC-2 cells. Results are presented as the means ± SD. *p<0.05.

We used a tissue microarray to detect the expression of HRAS by immunohistochemistry and found that HRAS was upregulated in the sunitinib-nonresponsive tissues of RCC (Figure 6A). The overexpression of HRAS could promote sunitinib resistance in RCC cells (Figure 6B, Supplementary Figure 6A), and the inhibition of HRAS by lonafarnib 35 could restore sunitinib sensitivity in RCC cells (Figure 6C, Supplementary Figure 6B). By the "rescue method", inhibiting HRAS eliminated the discrepancy in sunitinib sensitivity between the QPCT-overexpressing cells and the control RCC cells (Figure 6D). Furthermore, the knockdown of QPCT diminished the distinct difference in sunitinib response between the HRAS-overexpressing cells and the control RCC cells (Figure 6E).

Related literature reports found that HRAS was involved in the activation of multiple signalling pathways in tumours. Thus, we detected whether any signalling pathways related to sunitinib resistance were activated in QPCT-overexpressing RCC cells. We found that p-ERK was upregulated in QPCT-overexpressing RCC cells, demonstrating that the ERK signalling pathway was activated (Supplementary Figure 6C). There were many reports confirming that HRAS could activate ERK signalling pathways 36-39, and we verified that p-ERK was upregulated when HRAS was overexpressed in RCC cells (Figure 6F), while p-ERK was downregulated when HRAS was inhibited in RCC cells (Figure 6G). These results proved that HRAS could promote sunitinib resistance in RCC cells by promoting ERK phosphorylation. When ERK was inhibited by SCH772984 40-42, the sensitivity of RCC cells to sunitinib increased (Supplementary Figure 6D), indicating that the activation of the ERK pathway played a role in the resistance to sunitinib in RCC.

### High QPCT levels predict poor responses to sunitinib in RCC patients

**Figure 7:**
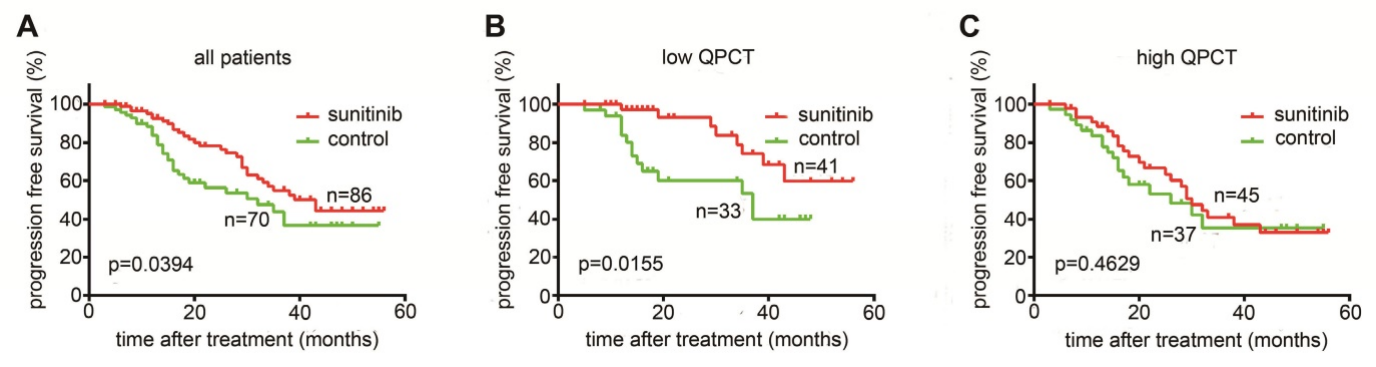
** High QPCT levels predict poor responses to sunitinib in RCC patients.** (A) Kaplan-Meier analysis of progression-free survival (PFS) in all patients (p= 0.0394). (B) Kaplan-Meier analysis of PFS in patients with a low expression of QPCT (p= 0.0155). (C) Kaplan-Meier analysis of PFS in patients with a high expression of QPCT (p=0.4629).

As QPCT was functionally involved in the response to sunitinib in RCC cells, we further evaluated whether the expression of QPCT in tumour tissues was associated with the response to sunitinib therapy. We measured QPCT levels in 156 RCC samples from 86 patients receiving sunitinib therapy and 70 patients receiving no drug therapy after surgery as a control group (Supplementary Table 8). We found that sunitinib therapy could prolong the overall PFS in RCC patients (Figure 7A) and that patients with low QPCT expression levels in tumour tissues had a more significant improvement in PFS after receiving sunitinib than those in the control group (Figure 7B). However, patients with high QPCT expression levels showed a poor response to sunitinib therapy (Figure 7C). Thus, the expression of QPCT could serve as an independent predictor of the response to sunitinib in RCC patients.

## Discussion

The mechanisms of sunitinib resistance can be roughly divided into the following: activation of the angiogenic signalling pathway, changes in the tumour microenvironment, increase in tumour invasion and metastasis, and the role of microRNAs and lncRNAs in the activation of other signal bypasses 43. Pro-angiogenic factors, such as Ang2, FGF, and PDGF, are upregulated in most cases of sunitinib resistance 44, 45. In fact, anti-angiogenic-induced hypoxia activates the mTOR pathway and induces HIF production, which activates the transcription HRE-containing genes such as VEGF, PDGF, TGF-α, EPO, MMP-1, EGFR, HGFR/cMET, cyclin D1, and SDF1 and its receptor CXCR4 46.

DNA methylation is involved in the regulation of gene expression and silencing and is closely related to many diseases and physiological processes, including tumours 47-50; it can be used as a diagnostic and therapeutic marker for many diseases 51-53. In our study, we found that the DNA methylation level was lower in sunitinib-nonresponsive RCC tissues than in sunitinib-responsive RCC tissues by the Illumina Human Methylation 850K Microarray and Sequenom MassARRAY Methylation. QPCT mRNA and protein were significantly higher in the sunitinib-nonresponsive group than in the sensitive group. In addition, DNA methylation could synergize with the transcription factor NF-κB to regulate the expression of QPCT and played an important role in the resistance of sunitinib in RCC. Perhaps the degree of DNA methylation in QPCT could be an indicator for predicting the sunitinib response in RCC patients. We also detected QPCT levels in the peripheral blood (plasma) of RCC patients who were resistant or sensitive to sunitinib therapy and found that the QPCT level in the sunitinib-nonresponsive group was significantly higher, indicating that the plasma content of QPCT might also be used as a potential biomarker for predicting the sunitinib response in RCC patients.

The QPCT gene encodes glutamine peptide cyclotransferase, an enzyme that performs posttranslational modification on proteins by converting an N-terminal glutamate to pyroglutamate. This modification renders the protein more resistant to protease degradation, making it more hydrophobic and more prone to aggregation and neurotoxicity 30. Limited data are available about the expression of QPCT in cancer. The analysis of microarray datasets identified QPCT as highly expressed in melanoma 54 and thyroid carcinomas 55-57.

HRAS is a member of the RAS family and has both activated and non-activated forms. Usually, HRAS is in the non-activated state, which is characterized by its combination with GDP. When the non-activated HRAS is stimulated by certain factors, the GDP turns into GTP; thus, HRAS changes into its activated form, promoting the activation of downstream signalling pathways 58, 59. Normal or mutated forms of HRAS are overexpressed in multiple tumours 60-65. In our study, we found that QPCT bound to HRAS and increased the stability of HRAS by reducing its ubiquitination degradation, thus activating the ERK signalling pathway and leading to sunitinib resistance in RCC. Therefore, QPCT and HRAS might become new targets for the treatment or reversal of sunitinib resistance in RCC.

In conclusion, we showed that QPCT, which is regulated by DNA methylation and NF-κB (p65), promoted sunitinib resistance by reducing the ubiquitination of HRAS, thus activating the ERK pathway in RCC.

## Supplementary Material

Supplementary figures and tables.

## Abbreviations

RCC: renal cell carcinoma
QPCT: glutaminyl peptide cyclotransferase
VEGFR: vascular endothelial growth factor receptor
DNMT: DNA methyltransferases
SAM: S-adenosylmethionine
NF-κB: nuclear factor-κ-gene binding
Decitabine: 5-Aza-2'-deoxycytidine
PFS: progress free survival
OS: overall survival
PCR: polymerasechain reaction
IHC: Immunohistochemistry
CCK-8: cell counting kit 8
ChIP: chromatin immunoprecipitation
Co-IP: co-immunoprecipitation
