# Supplementary material for: DNA methylation-regulated QPCT promotes sunitinib resistance by increasing HRAS stability in renal cell carcinoma
Source: Theranostics. 2019 Aug 14;9(21):6175–90. doi: 10.7150/thno.35572 (PMC6735520; doi:10.7150/thno.35572)
Supplement: Supplementary file 1 — Supplementary figures and tables. [file thnov09p6175s1.pdf]

Figure legends

Supplementary Figure 1. (A) Methylation levels of IRS1, SKI, PTK2B, C1orf86, B3GNT7, AP4K2, PRKCZ, and ACP5 in 10 pairs of sunitinib-responsive and nonresponsive RCC tissues by Sequenom MassARRAY Methylation. (B) CpG sites that had differences between the two groups in the IRS1, SKI, and PTK2B promoter regions. (C) Expression of IRS1, SKI, and PTK2B mRNA in 16 pairs of sunitinib-responsive and nonresponsive RCC tissues. (D) Representative results of western blot analysis of IRS1 protein levels. (E) mRNA expression of QPCT in RCC cell lines and HK-2 cells.
The results are presented as the means ± SD. $*p<0.05$.

Supplementary Figure 2. (A) qPCR and western blot analysis of QPCT siRNA interference efficiency in ACHN and OS-RC-2 cells.
Results are presented as the means ± SD. $**p < 0.01$.

Supplementary Figure 3. (A) qPCR and western blot analysis of lentivirus-QPCT overexpression efficiency in 786-O and A498 cells.
Results are presented as the means ± SD. $**p<0.01$.

Supplementary Figure 5. (A) ELISA analysis of QPCT distribution inside and outside (cell culture supernatant) the OS-RC-2 and ACHN cells. (B) Immunofluorescence analysis of QPCT (red) in OS-RC-2 and 786-O cells. Scale bar, 100 μm. (C) Signals of CBL, GAB1, HRAS, MAPK8, MAPK10, NAF1 and PTK2 in human proteome microarrays. (D) Co-immunoprecipitation of QPCT with CBL, GAB1, MAPK8, MAPK10, NAF1 and PTK2 in 786-O cells. (E) qPCR and western blot analysis of sh-QPCT efficiency in ACHN and OS-RC-2 cells (n=3).
Results are presented as the means ± SD. $*p<0.05$, $**p<0.01$.

Supplementary Figure 6. (A) Western blot analysis of plasmid pcDNA3.1-HRAS efficiency in A498 and 786-O cells. (B) Western blot analysis of lonafarnib (1.9 nM) efficiency in OS-RC-2 and ACHN cells. (C) Western blot analysis of signalling pathways related to sunitinib resistance in QPCT-overexpressing and control 786-O and A498 cells. (D) CCK-8 assay of QPCT-overexpressing 786-O and A498 cells treated with SCH772984 (4 nM) or QPCT-overexpressing 786-O and A498 cells after sunitinib treatment at the indicated concentrations for 48 h (n=3). The IC50 values are shown in the right histogram.

Results are presented as the means ± SD. *$p<0.05$.

Supplementary Table 1. Basic information of 4 pairs of sunitinib-responsive and nonresponsive RCC tissues

Supplementary Table 2. Basic information of 10 pairs of sunitinib-responsive and nonresponsive RCC tissues

Supplementary Table 3. Basic information of 16 pairs of sunitinib-responsive and nonresponsive RCC tissues

Supplementary Table 4. Basic information of 15 pairs of sunitinib-responsive and nonresponsive RCC tissues

Supplementary Table 5. Basic information of RCC patients used in immunohistochemical analysis in tissue microarrays

Supplementary Table 6. Basic information of 19 sunitinib-nonresponsive patients and 21 sunitinib-responsive patients of RCC from the Changhai Hospital

Supplementary Table 7. Basic information of 14 sunitinib-nonresponsive patients and 18 sunitinib-responsive patients of RCC from the Changzheng Hospital

Supplementary Table 8. Basic information of RCC patients with a low QPCT expression or a high QPCT expression

Supplementary Table 9. All proteins contained in the HuProt microarray

Supplementary Table 10. Proteins that may bind to QPCT in the HuProt microarray

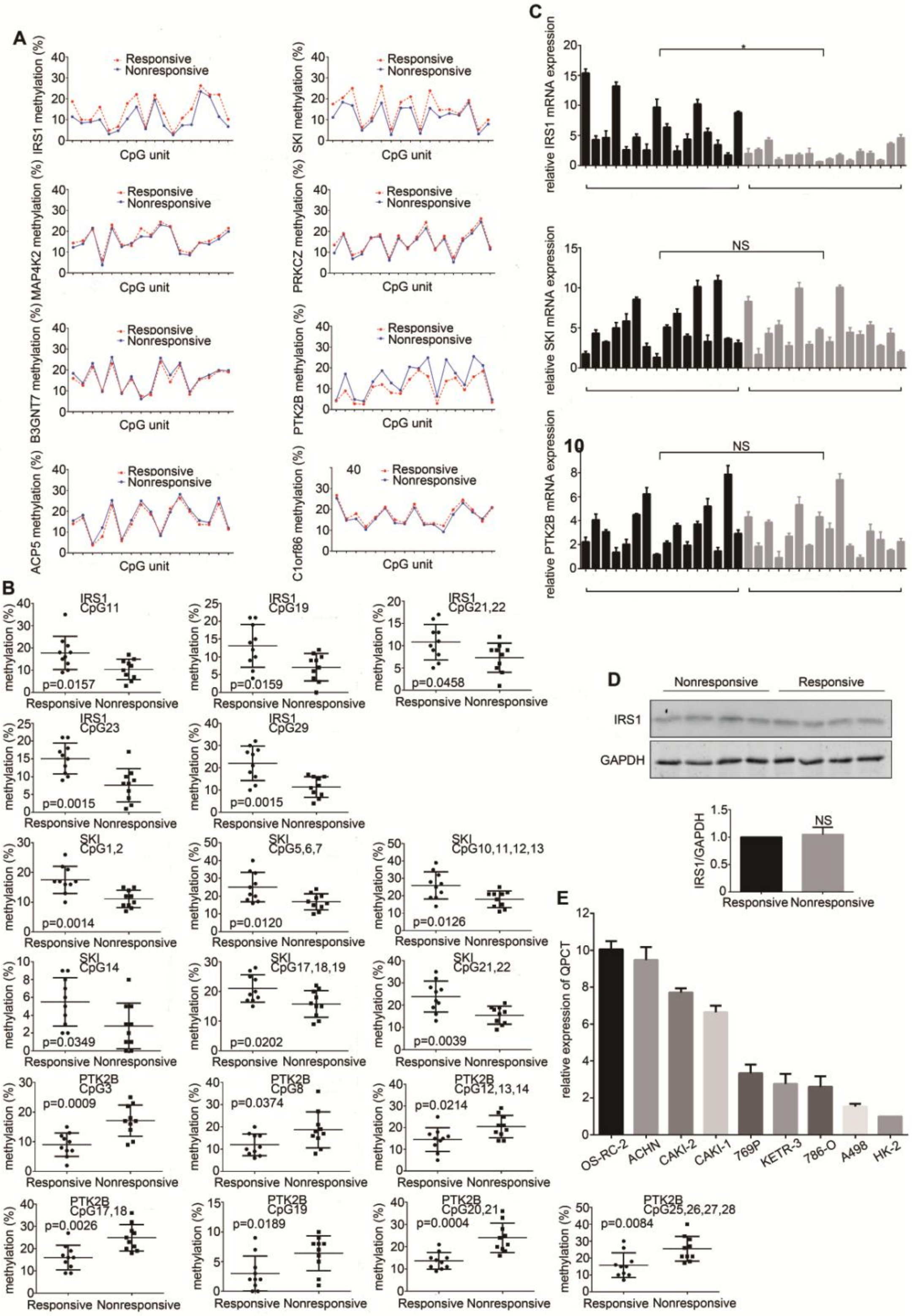
A
IRS1 methylation (%)
SKI methylation (%)
MAP4K2 methylation (%)
PRKCZ methylation (%)
B3GNT7 methylation (%)
PTK2B methylation (%)
ACP5 methylation (%)
C1orf86 methylation (%)
Responsive
Nonresponsive
CpG unit
B
IRS1 CpG11 p=0.0157
IRS1 CpG19 p=0.0159
IRS1 CpG21,22 p=0.0458
IRS1 CpG23 p=0.0015
IRS1 CpG29 p=0.0015
SKI CpG1,2 p=0.0014
SKI CpG5,6,7 p=0.0120
SKI CpG10,11,12,13 p=0.0126
SKI CpG14 p=0.0349
SKI CpG17,18,19 p=0.0202
SKI CpG21,22 p=0.0039
PTK2B CpG3 p=0.0009
PTK2B CpG8 p=0.0374
PTK2B CpG12,13,14 p=0.0214
PTK2B CpG17,18 p=0.0026
PTK2B CpG19 p=0.0189
PTK2B CpG20,21 p=0.0004
PTK2B CpG25,26,27,28 p=0.0084
methylation (%)
Responsive Nonresponsive
C
relative IRS1 mRNA expression
*
relative SKI mRNA expression
NS
relative PTK2B mRNA expression
NS
D
Nonresponsive
Responsive
IRS1
GAPDH
IRS1/GAPDH
NS
Responsive Nonresponsive
E
relative expression of QPCT
OS-RC-2
ACHN
CAKI-2
CAKI-1
769P
KETR-3
786-O
A498
HK-2

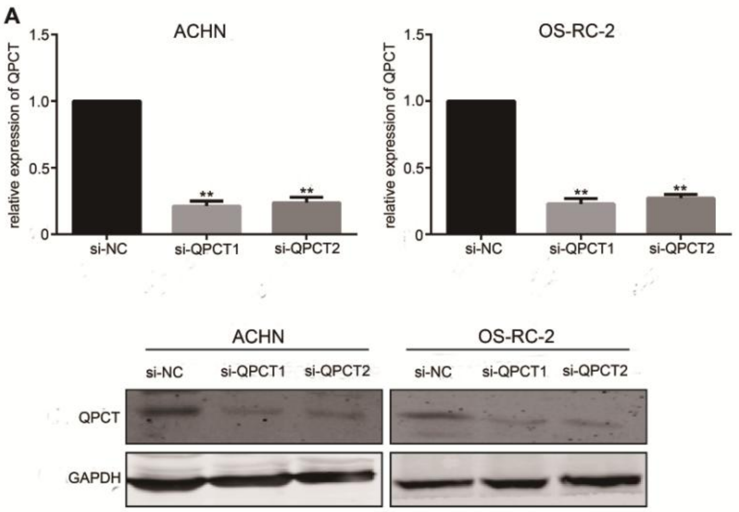
A
ACHN
OS-RC-2
relative expression of QPCT
1.5
1.0
0.5
0
**
si-NC
si-QPCT1
si-QPCT2
QPCT
GAPDH

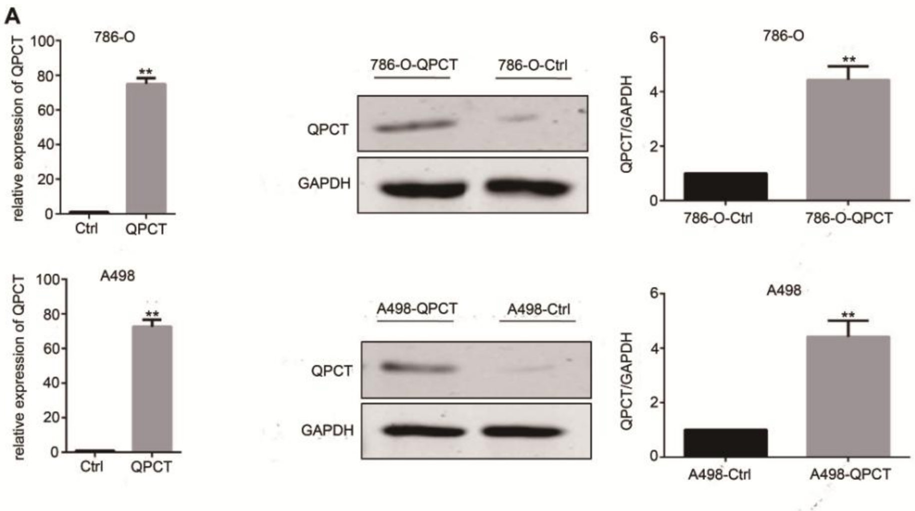
A
786-O
relative expression of QPCT
100
80
60
40
20
0
Ctrl
QPCT
**
786-O-QPCT
786-O-Ctrl
QPCT
GAPDH
786-O
QPCT/GAPDH
6
4
2
0
786-O-Ctrl
786-O-QPCT
**
A498
A498-QPCT
A498-Ctrl
A498-Ctrl
A498-QPCT

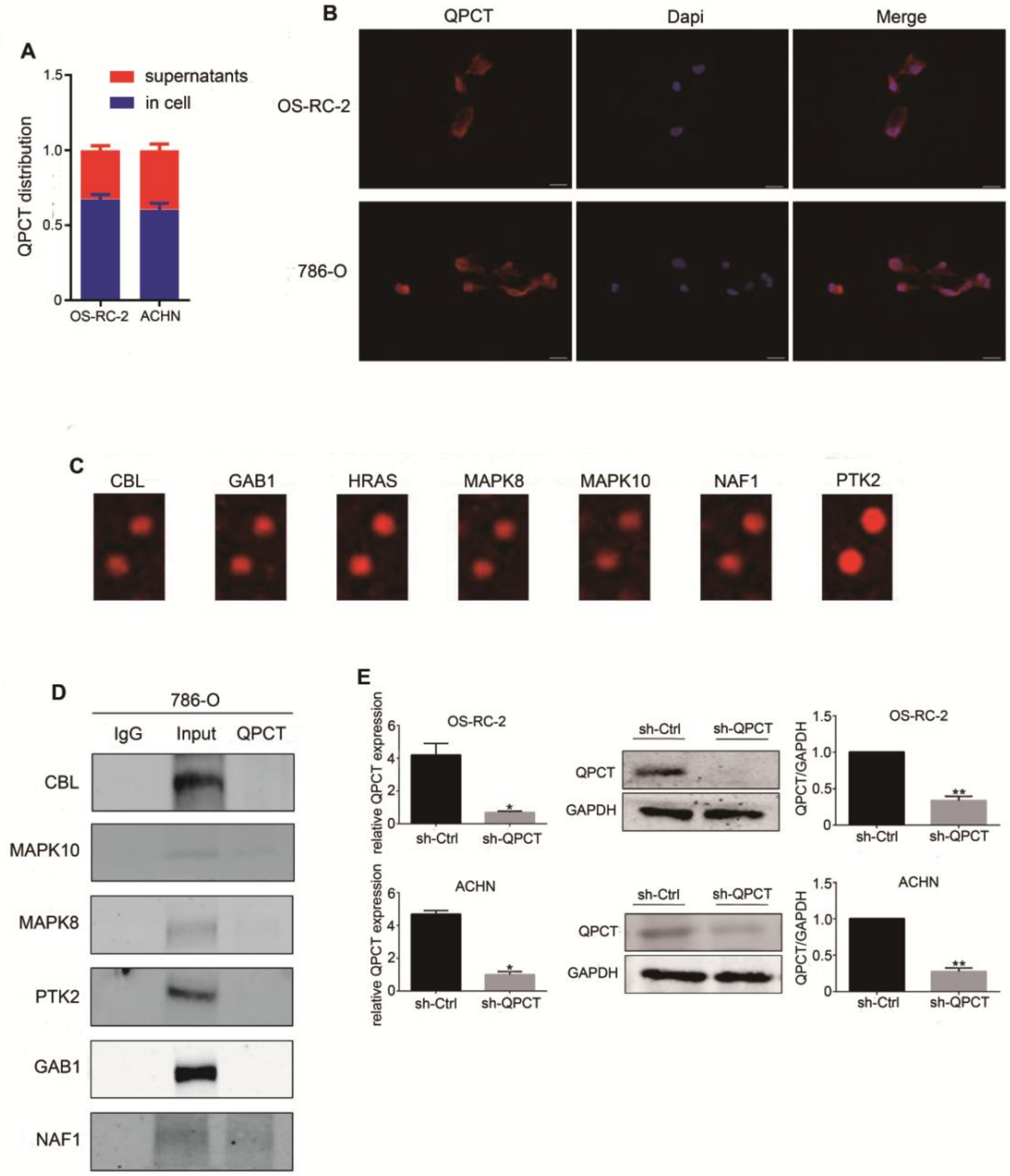

A
B
C
D
E
supernatants
in cell
QPCT distribution
OS-RC-2
ACHN
QPCT
Dapi
Merge
786-O
CBL
GAB1
HRAS
MAPK8
MAPK10
NAF1
PTK2
IgG
Input
relative QPCT expression
sh-Ctrl
sh-QPCT
GAPDH
QPCT/GAPDH

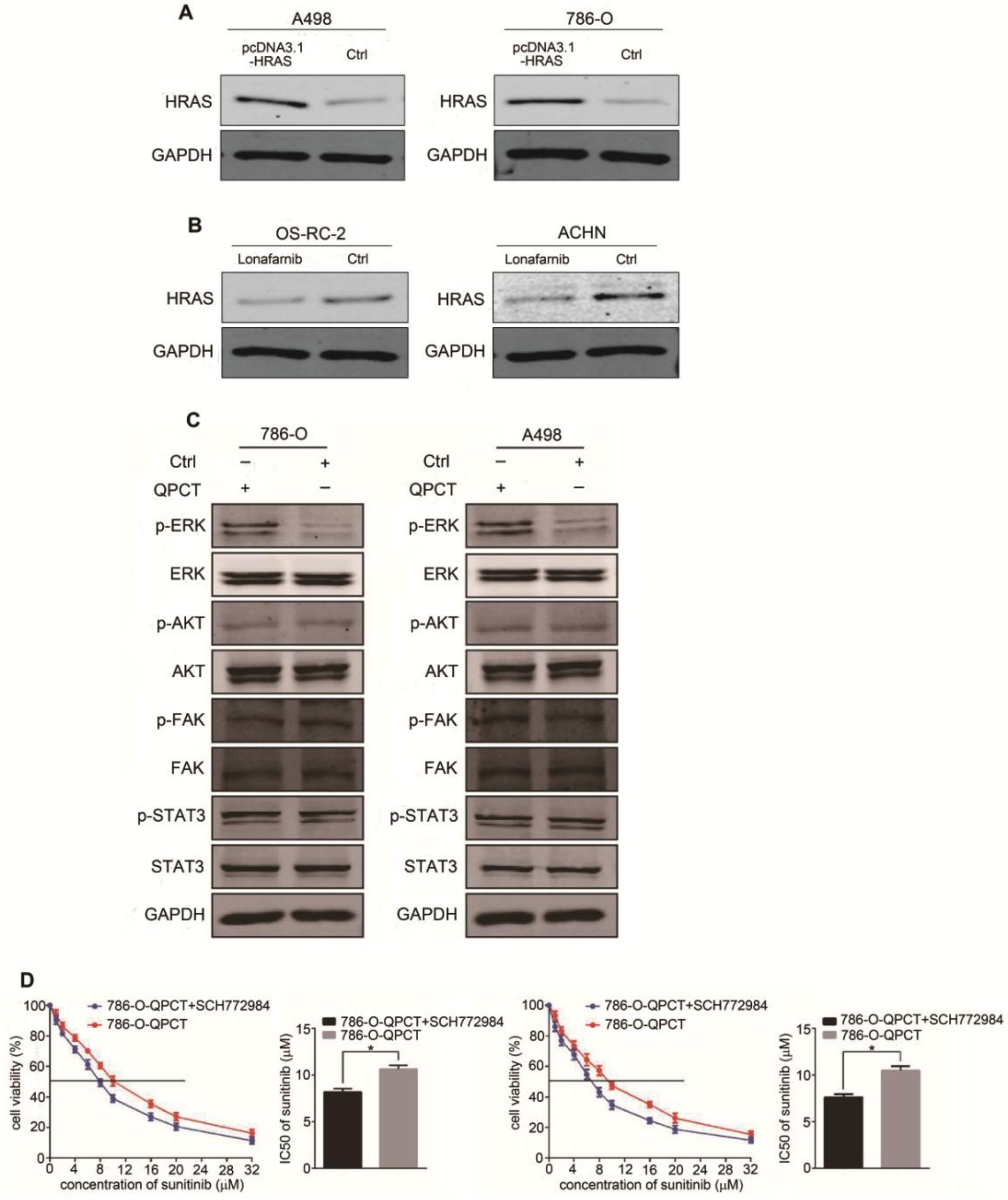

A
A498
pcDNA3.1 -HRAS
Ctrl
HRAS
GAPDH
786-O
pcDNA3.1 -HRAS
Ctrl
HRAS
GAPDH
B
OS-RC-2
Lonafarnib
Ctrl
HRAS
GAPDH
ACHN
Lonafarnib
Ctrl
HRAS
GAPDH
C
786-O
Ctrl − +
QPCT + −
p-ERK
ERK
p-AKT
AKT
p-FAK
FAK
p-STAT3
STAT3
GAPDH
A498
Ctrl − +
QPCT + −
p-ERK
ERK
p-AKT
AKT
p-FAK
FAK
p-STAT3
STAT3
GAPDH
D
786-O-QPCT+SCH772984
786-O-QPCT
cell viability (%)
concentration of sunitinib (μM)
IC50 of sunitinib (μM)
*
786-O-QPCT+SCH772984
786-O-QPCT
cell viability (%)
concentration of sunitinib (μM)
IC50 of sunitinib (μM)
*

Supplementary Table 1. Basic information of 4 pairs of sunitinib-responsive and nonresponsive RCC tissues

Patient ID	Age	Gender	Pathological diagnosis	Fuhrman grade	TNM stage	Treatment	Months of treatment	Best response
N1	67	Male	ccRCC	III	T3N0M1	sunitinib	5	PD
N2	63	Female	ccRCC	II-III	T2N0M1	sunitinib	7	PD
N3	72	Male	ccRCC	III	T2N0M1	sunitinib	8	PD
N4	54	Male	ccRCC	II	T1N0M1	sunitinib	4	PD
R1	48	Male	ccRCC	II	T2N0M1	sunitinib	27	PR
R2	69	Male	ccRCC	II	T1N0M1	sunitinib	25	PR
R3	57	Female	ccRCC	III	T3N0M1	sunitinib	23	PR
R4	70	Male	ccRCC	III	T2N0M1	sunitinib	28	PR

(N: sunitinib-nonresponsive, R: sunitinib-responsive, PD: progressive disease, PR: partial response)

Supplementary Table 2. Basic information of 10 pairs of sunitinib-responsive and nonresponsive RCC tissues

Patient ID	Age	Gender	Pathological diagnosis	Fuhrman grade	TNM stage	Treatment	Months of treatment	Best response
N1	67	Male	ccRCC	III	T3N0M1	sunitinib	5	PD
N2	63	Female	ccRCC	II-III	T2N0M1	sunitinib	7	PD
N3	72	Male	ccRCC	III	T2N0M1	sunitinib	8	PD
N4	54	Male	ccRCC	II	T1N0M1	sunitinib	4	PD
N5	66	Male	ccRCC	III	T1N0M1	sunitinib	6	PD
N6	48	Male	ccRCC	IV	T3N1M1	sunitinib	6	PD
N7	58	Male	ccRCC	III	T1N0M1	sunitinib	5	PD
N8	74	Female	ccRCC	II	T1N0M1	sunitinib	7	PD
N9	71	Male	ccRCC	II-III	T1N0M1	sunitinib	8	PD
N10	62	Female	ccRCC	II	T2N0M1	sunitinib	8	PD
R1	48	Male	ccRCC	II	T2N0M1	sunitinib	27	PR
R2	69	Male	ccRCC	II	T1N0M1	sunitinib	25	PR
R3	57	Female	ccRCC	III	T3N0M1	sunitinib	23	PR
R4	70	Male	ccRCC	III	T2N0M1	sunitinib	28	PR
R5	57	Male	ccRCC	II	T1N0M1	sunitinib	21	PR
R6	53	Male	ccRCC	II	T1N0M1	sunitinib	24	PR
R7	72	Male	ccRCC	II	T1N0M1	sunitinib	18	PR
R8	43	Male	ccRCC	II-III	T1N0M1	sunitinib	27	PR
R9	64	Female	ccRCC	I	T2N1M1	sunitinib	26	PR
R10	49	Male	ccRCC	III	T2N0M1	sunitinib	23	PR

(N: sunitinib-nonresponsive, R: sunitinib-responsive, PD: progressive disease, PR: partial response)

Supplementary Table 3. Basic information of 16 pairs of sunitinib-responsive and nonresponsive RCC tissues

Patient ID	Age	Gender	Pathological diagnosis	Fuhrman grade	TNM stage	Treatment	Months of treatment	Best response
N1	67	Male	ccRCC	III	T3N0M1	sunitinib	5	PD
N2	63	Female	ccRCC	II-III	T2N0M1	sunitinib	7	PD
N3	72	Male	ccRCC	III	T2N0M1	sunitinib	8	PD
N4	54	Male	ccRCC	II	T1N0M1	sunitinib	4	PD
N5	66	Male	ccRCC	III	T1N0M1	sunitinib	6	PD
N6	48	Male	ccRCC	IV	T3N1M1	sunitinib	6	PD
N7	58	Male	ccRCC	III	T1N0M1	sunitinib	5	PD
N8	74	Female	ccRCC	II	T1N0M1	sunitinib	7	PD
N9	71	Male	ccRCC	II-III	T1N0M1	sunitinib	8	PD
N10	62	Female	ccRCC	II	T2N0M1	sunitinib	8	PD
N11	53	Male	ccRCC	II-III	T3N1M1	sunitinib	4	PD
N12	44	Male	ccRCC	II	T2N0M1	sunitinib	6	PD
N13	43	Male	ccRCC	II	T1N0M1	sunitinib	7	PD
N14	67	Female	ccRCC	II	T1N0M1	sunitinib	7	PD
N15	72	Male	ccRCC	III	T3N0M1	sunitinib	5	PD
N16	75	Male	ccRCC	II-III	T1N0M1	sunitinib	6	PD
R1	48	Male	ccRCC	II	T2N0M1	sunitinib	27	PR
R2	69	Male	ccRCC	II	T1N0M1	sunitinib	25	PR
R3	57	Female	ccRCC	III	T3N0M1	sunitinib	23	PR
R4	70	Male	ccRCC	III	T2N0M1	sunitinib	28	PR
R5	57	Male	ccRCC	II	T1N0M1	sunitinib	21	PR
R6	53	Male	ccRCC	II	T1N0M1	sunitinib	24	PR
R7	72	Male	ccRCC	II	T1N0M1	sunitinib	18	PR
R8	43	Male	ccRCC	II-III	T1N0M1	sunitinib	27	PR
R9	64	Female	ccRCC	I	T2N1M1	sunitinib	26	PR
R10	49	Male	ccRCC	III	T2N0M1	sunitinib	23	PR
R11	54	Male	ccRCC	I	T2N0M0	sunitinib	22	PR
R12	58	Male	ccRCC	II	T2N0M1	sunitinib	26	PR

R13	42	Male	ccRCC	II	T2N0M1	sunitinib	24	PR
R14	68	Female	ccRCC	II-III	T1N0M1	sunitinib	26	PR
R15	65	Female	ccRCC	II	T3N0M0	sunitinib	19	PR
R16	70	Male	ccRCC	I-II	T2N0M1	sunitinib	23	PR

(N: sunitinib-nonresponsive, R: sunitinib-responsive, PD: progressive disease, PR: partial response)

Supplementary Table 4. Basic information of 15 pairs of sunitinib-responsive and nonresponsive RCC tissues

Patient ID	Age	Gender	Pathological diagnosis	Fuhrman grade	TNM stage	Treatment	Months of treatment	Best response
N1	67	Male	ccRCC	III	T3N0M1	sunitinib	5	PD
N2	63	Female	ccRCC	II-III	T2N0M1	sunitinib	7	PD
N3	72	Male	ccRCC	III	T2N0M1	sunitinib	8	PD
N4	54	Male	ccRCC	II	T1N0M1	sunitinib	4	PD
N5	66	Male	ccRCC	III	T1N0M1	sunitinib	6	PD
N6	48	Male	ccRCC	IV	T3N1M1	sunitinib	6	PD
N7	58	Male	ccRCC	III	T1N0M1	sunitinib	5	PD
N8	74	Female	ccRCC	II	T1N0M1	sunitinib	7	PD
N9	71	Male	ccRCC	II-III	T1N0M1	sunitinib	8	PD
N10	62	Female	ccRCC	II	T2N0M1	sunitinib	8	PD
N11	53	Male	ccRCC	II-III	T3N1M1	sunitinib	4	PD
N12	44	Male	ccRCC	II	T2N0M1	sunitinib	6	PD
N13	43	Male	ccRCC	II	T1N0M1	sunitinib	7	PD
N14	67	Female	ccRCC	II	T1N0M1	sunitinib	7	PD
N15	72	Male	ccRCC	III	T3N0M1	sunitinib	5	PD
R1	48	Male	ccRCC	II	T2N0M1	sunitinib	27	PR
R2	69	Male	ccRCC	II	T1N0M1	sunitinib	25	PR
R3	57	Female	ccRCC	III	T3N0M1	sunitinib	23	PR
R4	70	Male	ccRCC	III	T2N0M1	sunitinib	28	PR
R5	57	Male	ccRCC	II	T1N0M1	sunitinib	21	PR
R6	53	Male	ccRCC	II	T1N0M1	sunitinib	24	PR
R7	72	Male	ccRCC	II	T1N0M1	sunitinib	18	PR
R8	43	Male	ccRCC	II-III	T1N0M1	sunitinib	27	PR
R9	64	Female	ccRCC	I	T2N1M1	sunitinib	26	PR
R10	49	Male	ccRCC	III	T2N0M1	sunitinib	23	PR
R11	54	Male	ccRCC	I	T2N0M0	sunitinib	22	PR
R12	58	Male	ccRCC	II	T2N0M1	sunitinib	26	PR
R13	42	Male	ccRCC	II	T2N0M1	sunitinib	24	PR

R14	68	Female	ccRCC	II-III	T1N0M1	sunitinib	26	PR
R15	65	Female	ccRCC	II	T3N0M0	sunitinib	19	PR

(N: sunitinib-nonresponsive, R: sunitinib-responsive, PD: progressive disease, PR: partial response)

Supplementary Table 5. Basic information of RCC patients used in immunohistochemical analysis in tissue microarrays

Variables		QPCT Low expression (n = 74)	QPCT High expression (n = 82)	p-value
**Gender**				0.797
	Male	59	64	
	Female	15	18	
**Age**				0.794
	< 60y	50	57	
	≥ 60y	24	25	
**Tumor size**				0.777
	≤ 4cm	14	17	
	> 4cm	60	65	
**Fuhrman Grade**				0.934
	I-II	42	46	
	III-IV	32	36	
**TNM stage**				N/A
	I/II	0	0	
	III/IV	74	82	
**Tumor thrombus**				0.870
	No	55	60	
	Yes	19	22	
**Distant Metastasis**				0.754
	No	15	15	
	Yes	59	67	

Supplementary Table 6. Basic information of 19 sunitinib-nonresponsive patients and 21 sunitinib-responsive patients of RCC in Changhai Hospital

Patient ID	Age	Gender	Pathological diagnosis	Fuhrman grade	TNM stage	Treatment	Months of treatment	Best response
N1	72	Male	ccRCC	III	T2N0M1	sunitinib	8	PD
N2	66	Male	ccRCC	III	T1N0M1	sunitinib	6	PD
N3	48	Male	ccRCC	IV	T3N1M1	sunitinib	6	PD
N4	74	Female	ccRCC	II	T1N0M1	sunitinib	7	PD
N5	62	Female	ccRCC	II	T2N0M1	sunitinib	8	PD
N6	53	Male	ccRCC	II-III	T3N1M1	sunitinib	4	PD
N7	43	Male	ccRCC	II	T1N0M1	sunitinib	7	PD
N8	72	Male	ccRCC	III	T3N0M1	sunitinib	5	PD
N9	45	Male	ccRCC	II	T2N0M1	sunitinib	6	PD
N10	49	Female	ccRCC	II	T2N0M1	sunitinib	6	PD
N11	67	Male	ccRCC	III	T3N0M1	sunitinib	7	PD
N12	54	Male	ccRCC	II	T2N0M0	sunitinib	5	PD
N13	62	Male	ccRCC	II	T3N1M1	sunitinib	5	PD
N14	66	Female	ccRCC	II-III	T2N0M1	sunitinib	8	PD
N15	74	Male	ccRCC	III	T1N0M1	sunitinib	6	PD
N16	57	Male	ccRCC	II	T2N0M1	sunitinib	5	PD
N17	70	Female	ccRCC	II	T3N1M1	sunitinib	4	PD
N18	61	Male	ccRCC	III	T2N0M0	sunitinib	6	PD
N19	63	Male	ccRCC	II-III	T3N0M1	sunitinib	7	PD
R1	48	Male	ccRCC	II	T2N0M1	sunitinib	27	PR
R2	69	Male	ccRCC	II	T1N0M1	sunitinib	25	PR
R3	70	Male	ccRCC	III	T2N0M1	sunitinib	28	PR
R4	57	Male	ccRCC	II	T1N0M1	sunitinib	21	PR
R5	43	Male	ccRCC	II-III	T1N0M1	sunitinib	27	PR
R6	64	Female	ccRCC	I	T2N1M1	sunitinib	26	PR
R7	54	Male	ccRCC	I	T2N0M0	sunitinib	22	PR
R8	42	Male	ccRCC	II	T2N0M1	sunitinib	24	PR

R9	68	Female	ccRCC	II-III	T1N0M1	sunitinib	26	PR
R10	65	Female	ccRCC	II	T3N0M0	sunitinib	19	PR
R11	54	Male	ccRCC	II-III	T1N0M0	sunitinib	22	PR
R12	42	Male	ccRCC	III	T2N0M1	sunitinib	24	PR
R13	70	Male	ccRCC	II	T2N0M1	sunitinib	21	PR
R14	72	Male	ccRCC	II	T3N0M1	sunitinib	25	PR
R15	69	Male	ccRCC	III	T1N0M1	sunitinib	23	PR
R16	67	Male	ccRCC	IV	T2N1M0	sunitinib	17	PR
R17	47	Female	ccRCC	II-III	T1N0M1	sunitinib	26	PR
R18	58	Male	ccRCC	II	T3N1M0	sunitinib	20	PR
R19	43	Male	ccRCC	II	TNM	sunitinib	22	PR
R20	71	Female	ccRCC	II	TNM	sunitinib	26	PR
R21	56	Male	ccRCC	II-III	TNM	sunitinib	19	PR

(N: sunitinib-nonresponsive, R: sunitinib-responsive, PD: progressive disease, PR: partial response)

Supplementary Table 7. Basic information of 14 sunitinib-nonresponsive patients and 18 sunitinib-responsive patients of RCC in Changzheng Hospital

Patient ID	Age	Gender	Pathological diagnosis	Fuhrman grade	TNM stage	Treatment	Months of treatment	Best response
N1	56	Male	ccRCC	II	T2N0M1	sunitinib	5	PD
N2	59	Male	ccRCC	II	T3N0M1	sunitinib	4	PD
N3	62	Male	ccRCC	II-III	T2N0M1	sunitinib	6	PD
N4	71	Female	ccRCC	III	T1N0M1	sunitinib	5	PD
N5	66	Male	ccRCC	II	T2N0M1	sunitinib	3	PD
N6	47	Male	ccRCC	III	T3N1M1	sunitinib	7	PD
N7	60	Female	ccRCC	II	T1N0M1	sunitinib	8	PD
N8	55	Female	ccRCC	II-III	T1N0M0	sunitinib	6	PD
N9	43	Male	ccRCC	II	T2N0M1	sunitinib	5	PD
N10	49	Female	ccRCC	II	T3N0M1	sunitinib	5	PD
N11	68	Male	ccRCC	III	T1N0M1	sunitinib	6	PD
N12	66	Male	ccRCC	II	T2N0M1	sunitinib	7	PD
N13	58	Male	ccRCC	II-III	T1N0M0	sunitinib	4	PD
N14	73	Male	ccRCC	II	T1N0M1	sunitinib	6	PD
R1	58	Male	ccRCC	II	T2N0M1	sunitinib	18	PR
R2	70	Male	ccRCC	II	T1N0M1	sunitinib	23	PR
R3	46	Male	ccRCC	IV	T2N1M1	sunitinib	24	PR
R4	48	Male	ccRCC	II	T3N0M1	sunitinib	27	PR
R5	65	Female	ccRCC	II	T2N0M1	sunitinib	18	PR
R6	53	Male	ccRCC	III	T2N0M1	sunitinib	19	PR
R7	55	Male	ccRCC	II-III	T1N0M1	sunitinib	21	PR
R8	63	Male	ccRCC	II	T1N0M0	sunitinib	20	PR
R9	67	Female	ccRCC	II	T2N0M1	sunitinib	27	PR
R10	69	Male	ccRCC	III	T3N1M1	sunitinib	27	PR
R11	64	Male	ccRCC	II	T1N0M1	sunitinib	30	PR
R12	71	Female	ccRCC	II-III	T1N0M1	sunitinib	26	PR
R13	46	Male	ccRCC	II	T2N0M1	sunitinib	25	PR

R14	54	Male	ccRCC	II	T1N0M0	sunitinib	22	PR
R15	60	Male	ccRCC	II	T2N0M1	sunitinib	19	PR
R16	53	Female	ccRCC	III	T1N0M1	sunitinib	21	PR
R17	70	Male	ccRCC	II-III	T1N0M1	sunitinib	26	PR
R18	50	Male	ccRCC	II	T2N0M0	sunitinib	25	PR

(N: sunitinib-nonresponsive, R: sunitinib-responsive, PD: progressive disease, PR: partial response)

Supplementary Table 8. Basic information of RCC patients with low QPCT expression or high QPCT expression

Variables	Low QPCT expression (n=74)		p-value	High QPCT expression (n=82)		p-value	All patients (n=156)		p-value
	Control (n =33)	sunitinib (n =41)		Control (n =37)	sunitinib (n =45)		Control (n =70)	sunitinib (n =86)	
**Gender**			0.688			0.948			0.750
Male	27	32		29	35		56	67	
Female	6	9		8	10		14	19	
**Age**			0.882			0.892			0.996
< 60y	22	28		26	31		48	59	
≥ 60y	11	13		11	14		22	27	
**Tumor size**			0.458			0.713			0.441
≤ 4cm	5	9		7	10		12	19	
> 4cm	28	32		30	35		58	67	
**Fuhrman Grade**			0.898			0.735			0.874
I-II	19	23		20	26		39	49	
III-IV	14	18		17	19		31	37	
**TNM stage**			N/A			N/A			N/A
I/II	0	0		0	0		0	0	
III/IV	33	41		37	45		70	86	
**Tumor**			0.			0.			0.

**thrombus**			800			642			609
No	25	30		28	32		53	62	
Yes	8	11		9	13		17	24	
**Metastasis**			0.688			0.310			0.315
No	6	9		5	10		11	19	
Yes	27	32		32	35		59	67	

Supplementary Table 9. All proteins contained in the HuProt microarray

Name	Name	Name	Name	Name	Name	Name	Name
RAB8A	IFT22	ATP6V1C1	STMN3	POLR2C	RAG2	DIDO1	CMBL
ZBTB8A	AP1M2	ND	SLC35B2	CYP4B1	HSFX1	HEY2	LMBR1L
TUBB2B	TUBD1	NOLC1	GPRC5A	UGT1A10	NDUFB5	TTR	DEFA3
DCAF10	C1QA	ELOVL3	EMP2	IgG647	NAGK	TYROBP	LEPROTL1
KJ903335	MED30	SPN	SYBU	DHX40	RGMA	ITM2B	PLIN3
FBXO31	DRG1	TMEM230	DEF6	GNB1L	SCG5	PSMA6	MED6
ANXA8	TMEM80	RPL13A	RHPN1-AS1	EDIL3	AKNAD1	KJ903727	LDOC1
NAPA	MLPH	CNN1	ERBB3	GAP43	HSP90B1	PLS3	FADS2
PIH1D1	RNMTL1	ST6GALNAC1	DTNBP1	BAALC	ASH2L	RPS11	CD47
IFT46	BET1	LSMEM2	PEX2	CETN2	TBCE	MPPE1	BMP7
SMAD3	RNH1	DUSP3	PIP5K1B	DCLRE1B	LAIR1	PLIN2	CYR61
NCKAP1	IgG488/594	CDC42	STK3	CLIC2	EFNA1	ACTL6B	NAPSA
OLA1	VTI1A	HLA-DOA	MTAP	HSPA5	SORD	CCDC90B	POC1B
SHKBP1	MAN1B1	ARF3	BSCL2	BABAM1	RUVBL2	CNDP2	PDLIM4
CPVL	TBXAS1	ADGRL1	NUDCD2	ORM2	ELF5	C10orf53	BCL2A1
PLEKHA8P1	NME2	GMCL1	TOLLIP	COX6B1	ATP5C1	ANTXR1	CLDN14
ZNF302	RWDD4	LUC7L2	CEBPG	SKP1	BRK1	AIF1	SERPINA4
CWC27	GGPS1	USP5	CYB5B	BYSL	PAX8	PSMB2	SAP18
PPP3R2	RING1	WWOX	AHDC1	FNIP1	RAB40B	ENSA	ARF5
ABT1	C11orf1	PCIF1	BNIP3L	PINX1	AIG1	FCN3	ACSBG2
LDHC	ORC4L	COX4I1	SAA4	WBP11	TFCP2	MRPL4	AGTR1
SNX7	WAC	DLEU1	TCF21	C19orf25	WBSCR27	UBE2Z	GPX7
TRIM63	PLEKHJ1	MECR	TMEM182	AP3M1	FKBP8	CBS	APH1A
ZDHHC3	UBE2A	SCRN2	ABAT	AP1S3	ETV3	DUSP6	PPIL3
UFD1L	RANBP3	CPLX1	MLF2	CLIC5	ADCK5	CD2	ELP4
VSTM2L	ATF6	THRSP	SREK1	PRKRIR	SGK3	PPP2R4	KJ905805
FAIM	TNFAIP8L2	SNX27_	ZNF669	MRPL48	PEX11A	HDAC11	RSL24D1
RWDD3	ARL6IP6	CA1	CBWD1	POLR2E	NAT9	TAP2	TCN2
S100A8	TRAPPC4	EIF3H	RAC3	UBE2Q2	IMUP	MOB3A	KIZ
LRRTM4	MLNR	CAMK4	STK17B	PRMT8	PMEPA1	GIN1	LIN7C
SYF2	C1D	TBCB	HSD17B12	C16orf78	F13A1	NPPA	CPA2
C6orf106	SF3B5	SMIM14	ATAD1	CRELD1	WAS	FGF21	MVP
GALM	PUF60	GID8	CTSE	STAC3	SAE1	SH2D1B	TMEM68
PLOD3	CTSB	TAGLN3	RPS14	APOBEC3C	AIF1L	AEBP2	BBS10
GCSAML	USP14	EIF3L	DBNDD1	TPPP3	CRIP2	ARFGAP2	ASPA
NXPE1	DPY30	TMEM216	MYCL	FATE1	AHDC1	MTHFD2L	hMDM2
RAB14	RGR	KCNS3	MRPS11	IRF1	CYTH4	PTGS2	DDX41
CHMP7	CPSF3	ZNF32	TMEM167A	ESR2	ZNF486	PYROXD2	CCT3
RAD51	GINS2	UBE2T	BC000579	STX7	LTV1	FAM47A	MED17
NECAB1	TRIM74	SMRP1	ARL14	PDILT	MATK	RIPK2	ULK4
DERL1	SLC30A5	TMEM98	TIMMDC1	OMG	FAM76A	PYGB	USP25

PSAP	NOSIP	PWP2	MAGOHB	ERVMER34-1	PNLIP	ZNF566	PSAT1
MAGEA3	SNRPF	MLLT11	KLHL3	FAM124A	SULT4A1	LAPTM4A	VAMP3
POLR2F	RanBP2deltaFG	PMS2P5	FGFBP1	HLA-E	ADH4	C7orf33	STX18
TCTN2	SQLE	OSER1	DXO	CTSG	NDUFA6	KLK5	PNKD
NSG1	MIS12	CEP290	PRDX2	FAM46C	AMDHD1	SRSF1	DHRS13
SNX24	ZNF747	CROCCP2	SLC22A18AS	MROH5	TPD52L3	IRAK4	AGPAT4
SCAND2P	RTN2	AZIN1	DNAH6	MRPS18A	SNX3	CMSS1	NALCN
FIGF	KJ900890	NPM1	AIMP2	SNX6	GABARAPL2	IFT81	TSN
EFHC1	TYR	PRRC2B	MLC1	IMP3	NICN1	GPR137C	GSK3B
CLK3	AK3	HIGD2A	TGIF1	LDHB	SLC16A1	AFF4	RAB39B
PLEKHA7	TSPAN1	WDR77	EU794580.1	CMTM2	SLC23A1	METTL2A	TUBB4B
PIN1	ADIRF	TRIP13	ATRX	IST1	CD83	HSPA13	SLC1A1
ANAPC15	PPP1CA	FAM64A	KRTAP3-2	RHOA	MPP1	GABRA5	FGF7
SLC25A20	EMCN	STAG3L4	NDUFB3	RNF11	IGF2BP2	TM9SF4	FAM104A
KJ901002	RFX4	NUDT22	PHYKPL	ARPC1B	SLC10A3	AK074549.1	NXT1
NARG2	RAB25	MTMR9	NR1I2	CLEC7A	KJ901885	PEX7	ZCCHC10
KRTAP6-3	HKDC1	PPP2R5C	AMDHD2	ZMAT4	TMEM27	WDFY3	CFHR1
RNF2	CANT1	BLVRA	KIF2C	PPIL2	SPPL3	BEST3	SSX3
PDCD2L	INTS10	PRDX3	DIMT1	THAP4	AJUBA	IGKC	AKT1
KCNIP2	TM4SF19	MRPL30	PHB2	SF3B6	Biotin-BSA	SFN	CBR1
UROS	SPC25	NTAN1	ZMYM3	TSPYL4	TTC33	PARL	CDC16
CNN3	RNF25	NLN	ANKZF1	TIMP1	DAP3	BC033582.1	RPL14
CAPN3	CD74	KJ905802	TCP10L	MSMB	ZNF44	TSHB	RAB3D
KCNV1	RPS8	FBXL3	VPS50	TWSG1	PRPF40A	BRINP1	PGS1
PHF19	NABP2	CAPRIN2	PMEL	LGMN	ANXA3	SERPINB6	DCTN2
EBI3	JAGN1	RP11-354P17.9	MIB1	LDHAL6A	PSMA3	FAM218A	RPL11
BSA	AAK1	MBIP	MAPK3	PAK4	ATP6V1E2	AKTIP	N6AMT1
PPP2R5A	NDUFA4L2	FRS3	TMED3	CSRP2BP	FXYD5	NECAP2	AIM2
RAB39A	LRRIQ1	CPA1	ELP3	RPRM	PSMD3	PDHA1	PHTF1
KDSR	SPAG6	USP24	ZNF586	LOC79160	THEM6	UBA5	BSPH1
IFIT3	NFKBIA	ATP6V0A2	RELT	NEIL2	SCAMP3	LYRM1	NXT2
ERH	NCLN	SLC35A1	PARD6A	MRPS12	SUMO3	PSMD13	SPPL2B
BCL7B	UQCR11	ERP27	LYG1	BLOC1S6	PNMAL1	DTWD1	UTP4
PLA2G4D	C3orf64	ZMYM6NB	ANKFY1	GRK6	PPP1R14A	FIBIN	GINM1
CTC1	IGHG1	SCG3	USP48	CD58	EXO1	NIFK	CDCA7
HNRNPC	CD70	RUVBL1	ZSCAN32	TOR3A	LDB2	SEC11A	DDX39A
CDC42SE1	C8orf22	BC013069.1	KJ902259	KJ906227	CREBZF	PRMT2	SYK
RPLP1	GLOD4	RMND5A	CPLX3	DAZAP1	VPS45	SUB1	SLC38A1
DEDD	RNF111	XM_008974131.2	CRYAB	GPAA1	ALS2	NUDT2	HNRNPK
KXD1	ENY2	CCDC54	TMX1	TCEB2	LAMA4	USP10	AGT
MED12L	RAB8B	GPR183	NCK2	CYBB	HAVCR2	RALBP1	SSBP2
MAGEA4	FNTB	SNAP25	SFT2D1	GMCL1P1	NDFIP2	HLA-DPB1	NFYB
SMPD2	CALCOCO1	DNAJB1	ADI1	FAM50A	PTP4A1	STMN1	PQLC3
SCGB3A2	WAPAL	KJ901593	TNNI2	GCG	Buffer	ARL8B	EDNRB

RCVRN	RPS6	PRUNE2	LTBR	CHCHD3	SEPT10	ROPN1B	GDPD5
ZNF394	STX10	HMGN2	LXN	FDFT1	DPH2	PSMB7	CDC42EP3
MYL6	ASAH1	U2AF1L4	C10orf32	ARV1	UBA6	CAPS	ANKRD7
H1	COL4A3BP	CKS2	CRK	YEATS4	CHI3L1	RPS15A	NKD2
KLHL1	EFEMP1	C11orf54	RBBP9	BAMBI	GLYAT	DERL2	CD33
FKBP14	TVP23B	NUCB1	KJ904383	KLF6	COX15	SMIM7	MTA1
PDGFD	DIRAS1	MANBAL	ATPIF1	ANXA4	URGCP	FES	DUSP26
PRELID2	DENND1B	CRABP2	FAM198B	ANXA2	FUZ	DDIT4	PUM3
GRAP2	HADHB	RNFT1	NAA20	COA3	B4GALT3	CRYZL1	EEF1A2
BIRC5	RAB23	CYSTM1	CRP	C12orf65	FAM131A	SIGMAR1	H2(A+B)
TRIM5	PLA2G16	LRRC61	DNAJB14	ACSS3	RAB3C	TRIM43	ETS2
STT3B	ANKS6	TAF12	SDS	CEP70	CDC37	ZDHHC9	GPR89A
SMOX	TTC4	RCL1	DCX	TMEM252	NIPAL3	SNCG	GHRL
SESN2	PLA2G4C	CALML4	KJ901248	NEK11	TAOK3	PPP6C	MEMO1
CEACAM21	PSPC1	C1GALT1C1	CCDC148	PAK1IP1	APIP	PPIA	BDH1
MTAP	KJ903277	RQCD1	GSS	SHANK2-AS3	DMAP1	PAFAH2	FARSA
C1orf64	GADD45G	TLE6	SLC23A2	S100A3	RAB5A	TMEM125	THAP1
H3	HPCAL1	LGALS3	PDE6D	OLR1	YAE1D1	CD14	LYPLA2
ETFA	WDR33	AGTRAP	H2AFZ	OSTC	NFE2	CNOT10	PSMD8
OTUB2	CBX5	PSMC5	ACKR3	EXOC3-AS1	CYB5R3	SP100	SRCRB4D
MMGT1	ABCC9	CAPN2	TCF19	ERO1A	RABL3	ZNF556	ADD1
RNF113A	KLK6	ANKRD1	TFPI	CSNK1E	RAB6A	SPCS1	MED9
NPPB	GOSR2	VPS29	KIAA1147	CEP63	C12orf57	C15orf43	OPALIN
TSPAN9	H4	DUSP10	PPP1CC	OMA1	KLHL2	FAU	CAB39
PMPCB	EDN1	CAP2	GTF2A2	MEIS3	AFP	RPSA	RNASE1
ZSCAN16	KLF10	POMZP3	DCAKD	PIK3R3	ATP5G2	DPM2	SLC26A6
KJ903767	ERa	RRAGD	RAPGEF4	KCNJ8	SCO2	ZNHIT6	IFT43
PPIG	AGK	LOC100132167	G6PC3	C4BPA	CAPZA3	LUM	COL2A1
CELA2A	RNF220	CCM2	FHL3	FLI1	MRFAP1L1	NDNF	DDX60
FRZB	CXCL6	TIMP4	GJB4	ZCCHC13	YWHAB	YWHAH	MVK
SRP54	KJ901005	TMEM106A	SLC39A8	M1AP	TIA1	GLO1	LRIF1
DHRS12	RFC4	JKAMP	RELA	IFNGR1	RPA2	MAGEA10	TRIM51
MSANTD3	EIF5A	TMEM14B	FAM216A	SH3YL1	PPID	IGFBP4	PVRIG
HSPB8	GGCT	DCTD	EIF3E	GTF3C6	KCTD4	MED28	IL32
CST1	F3	KLK1	IFRD2	CENPO	MAGEA11	GOT2	PIGO
PHF7	ERCC6L2	CCL19	TMEM258	NUSAP1	LSM6	AMY2B	GABRB1
SERPINA6	ZNF226	PRNP	LMBR1	BC013178	CUTC	ZC3H15	DNAJB3
PIGM	PAAF1	ZNF207	ATG4B	UQCRC2	ANAPC5	LOC284912	SLC26A1
CCDC28B	CPXCR1	CD207	TTC26	HLA-DRA	HAAO	C1orf158	FGGY
AK6	PDK3	MCTS1	RAB34	SLC7A11	KJ903398	DDI1	EXOSC8
EIF4A2	UTP15	SPATC1L	PDIA3	HBG1	NFE2L2	TMEM138	CDC123
UBIAD1	CD44	PCID2	RPS10	CHRDL1	PLEKHS1	TMEM55A	L3HYPDH
HMGN1	KJ903140	RAB33A	ARL17A	MRPS25	TMEM33	KJ901485	PCDHGC3
BC018749.1	SLCO4A1-AS1	SPATA17	SPAM1	CAPNS2	TRMT2B	CELA3A	FAM53C

PAGR1	APOD	OAT	CFL2	CCL22	LSM7	MYOG	NUMB
SDHAF2	GPN3	PLCD4	RTN4	CCL20	PHC3	RBMS1	CXADR
EBAG9	RPL35A	MRPS6	AVL9	TPGS2	IER3IP1	MGLL	CDH6
OS9	RAD51C	HSD17B10	SPTSSA	FPR2	PDZD3	GLYATL2	KRBOX4
FLYWCH2	DPCD	ARHGDIB	MAP1LC3B	SGK2	CAMK1	PTPN6	CALN1
MITD1	NXPH3	ANGEL2	FCHSD2	PTS	STAM2	GZMM	MRPS18B
HJURP	C9orf16	GTF2F1	THYN1	ODF2	GPR162	BTC	IL15
POLR2J	EFCAB7	SERPINA1	ZFAND3	AK4	RIOK3	GATSL3	TMEM186
C12orf4	CKAP2	DNAAF5	ATP5F1	EIF4E	LHFPL5	CD247	CCNL1
TMEM55B	ISY1	INHBE	IFNAR2	CDKN1A	IFITM2	NOL4	ING3
SIVA1	ARL6IP4	MRPL10	POLR2K	RNF186	MYOT	TOE1	SKAP2
TMEM185A	CCNE2	PDRG1	FAM107A	AAMDC	PLEKHO1	ACAD11	ELK3
NDUFV1	RPS21	FMO9P	SERPINA3	FOXM1	TMEM38B	HSPA4	CD63
EMC4	PDZD7	CYTL1	LOC401152	C1orf123	MAF1	HINT3	ORM1
C2orf15	DIRAS3	DVL2	CHRNA3	CSRP2	CCND1	USP15	C14orf93
EIF3M	KIF6	ENOX1	FAM71B	C11orf70	TOX2	HNRNPF	ILF3
XM_002816682.3	SLC25A19	TUBG1	FBXO8	LRRC8D	C1QTNF7	MED29	MRPS5
MRO	PCNX4	UBE2G1	RACK1	GUK1	RAB13	RSU1	SEPT1
NAP1L2	ZNF671	CTH	WIPI2	CCDC28A	SOCS2	FABP4	RPS3A
FAM27B	CPTP	SETD3	FAM103A1	CNOT7	MCAM	ZNF257	PLXNA4
MUTYH	CLPP	ZNF426	NR4A2	RPL27	TNFSF13B	CDH19	SIX2
VSIG4	NECAB2	ZFP36L1	RPL26L1	C6orf48	CXXC1	SUMO2	PARP11
CHD2	TUFT1	PBDC1	AUNIP	RBM3	SF3A3	KCNE1	TMEM242
ERLEC1	SERPINE2	OSBPL11	ARC	HN1L	PSMC3IP	C3orf14	CMPK1
ULK2	CLEC4E	FAM126A	ZMYND19	NXNL1	ELMOD3	RABGEF1	ZNRD1
ZG16B	ERCC1	C19orf47	TPMT	HAUS3	CMTM6	TGOLN2	ZCCHC7
PAFAH1B2	GTF3C5	GDAP1	MITF	XM_012504148.1	NMU	NME5	RPS6KL1
SYTL2	NDUFA8	TCEAL1	KPNA1	PCTP	VPS26A	CYP2W1	SCARB2
POLR1A	ZNF385D	TMEM129	ISG15	AREG	CCDC67	ASS1	COMMD5
NECAP1	MYCBP	PDCD10	TMEM167B	PLEK2	AGFG1	SERINC1	AP2M1
S100B	SSBP3	RAB3B	CSRP1	FBXO6	GYPE	CERS4	PAX6
APPBP2	C19orf48	C16orf59	LDHAL6B	CLGN	STK40	IFNGR2	ASB6
KRT17	METTL3	PCOLCE	UBAP1	ZNF10	GJA4	YS049	SPIC
FCER1A	FAM174A	RPH3A	CAMKV	PTPN22	WSB1	SDR42E1	C8orf4
PELI2	C18orf25	RAB32	MEF2A	B4GALT7	UHRF2	CETP	SMAP1
RPL7	PLP1	BTN3A2	LMO4	RPLP2	ADAMTS4	SMUG1	AHNAK
ST3GAL1	HBP1	ZNF622	DNAAF3	PRSS2	TRIML1	NTMT1	DDOST
C9	ALG9	NDRG2	FTH1	DMKN	CUX1	HNRNPLL	PRIM2
COTL1	TEX264	PPARA	PAX9	PRR14	EIF2S2	RBMY1F	CGREF1
HPS1	RPF1	GAGE2D	NUP160	AP3S2	IG2R	UBE2L6	RAB18
CD97	P2RX7	RPL15	STAMBP	ZFAND1	C3orf49	SLC35F5	TUBA1B
RBM4	INTS9	NDUFA3	PROK1	MTERF2	GMPR2	RRN3	FAM60A
PDIA4	RNF24	GMDS	TNFAIP8L1	TRMT12	C9orf97	FAM188A	MS4A6A
IGL@	RPL7A	PTHLH	CKB	SLA	AK9	C14orf1	PLCB2

F8	NAV1	EIF1	FDPS	EIF4H	PARVA	ATF6B	DSCC1
UBE2D4	EIF3D	EXOSC3	COX7C	PRSS50	ITM2C	ASNSD1	PEX26
NR3C1	CMC4	MCEE	IGHD	SERPINH1	BAG1	NABP1	HAUS1
CDC25C	BANP	TOR1A	BPNT1	OSBPL2	CCNG1	RPL10A	CORO1C
COL25A1	LRRC2	RBM22	NOP9	CLEC4D	PPDPF	NHLH1	RBKS
JHU00131	PLPP3	SMAP2	LRRC28	ANKRD22	C14orf159	TMEM174	CSN3
COX7A2L	SH3GLB1	EIF2B3	MYL7	LILRA3	DAZAP2	BDH2	ZSCAN5A
CBR3	NSDHL	ZNF23	C1orf162	SERPINF1	DBT	STAT3	EIF4A3
MRPS33	IL37	GALK2	AMD1	C1QC	TRPM8	PROM1	PDYN
OSM	TAZ	SH3BP5	PSMC4	S100A4	AKAP17A	CLEC2B	PGM3
WIPF1	TAGLN	CLDN5	HYOU1	CDK17	TRIB3	HNRNPUL1	OLFM1
AMOTL2	RASGEF1A	OR7E91P	FKBP1B	MRPS7	DOK1	SNRNP27	QTRT1
RAB3IL1	RBFOX2	OARD1	LRRC20	DYDC1	UBE2V2	CAV2	RRP8
NONO	DCXR	CCKBR	CLUAP1	MRPL19	SGCB	OSTCP1	AXIN2
CFAP61	RASD2	CX3CR1	CACYBP	KPNA4	BC017762	COL9A1	TFDP2
GATAD2A	PP2D1	MARCH8	S100A10	RXRA	TBC1D7	COLEC11	TWF2
C11orf98	CORO2A	FAM127B	ZNF502	MRPL36	IGHG4	FRMPD2	TWIST2
FAM71E2	NDFIP1	STAC	CCL5	LGALS1	SLC44A4	PECAM1	KCNE4
IKZF1	KIAA1456	MDK	FBXL18	IL10RA	RSRC1	SULT1A1	KEAP1
TUBA1C	PSMD9	NAA10	BRMS1L	PDHA2	POGLUT1	FBXW9	ENC1
CDH11	BUB1B	ACP6	NUBPL	CORO2B	HIST1H2AG	PBRM1	DEPDC1B
BAZ2B	SPAG16	DPH6	UBE2I	WDR20	PDLIM3	ADIPOR1	ORMDL1
MYL9	R3HDM1	PPP2R5D	COMMD1	ACBD6	TMEM136	POU5F1	UBE2Q1
FAM175A	CCDC42	NRBF2	EMC9	TECR	ABI1	NHEJ1	PDZD11
RPL13	PTTG1IP	PHF21A	MUL1	RARG	POLR2I	FGL2	CSTF1
CALCOCO2	PNRC2	HIST1H2BK	METTL1	CD5L	GPR182	TAF1D	NAA16
ADCK4	ABCD4	FAM19A4	ANK1	TPTE	STYXL1	NAIF1	SLC25A32
SNAPC1	AMACR	ZCCHC4	CLDN10	ANAPC16	ICA1	KLHDC4	C14orf79
SARNP	PEX16	C11orf49	IFITM3	DHPS	HECTD3	LGALS8	C11orf68
PANK3	RAP1GDS1	ATP6V1D	GH2	ACMSD	RBM23	TADA1	AGA
DHRS1	FAM189B	EPT1	RP11-385F5.2	NUP62CL	KJ902696	METTL16	DLK2
MIF	LINC00521	FAM212B	LIPA	SLC22A18	BARX1	CBX6	LINC00341
PYM1	PLSCR1	LGALS14	WSCD1	TRIM9	C1QTNF6	TSC22D3	MOCS3
MPI	SNX12	SOD2	IGSF6	GCAT	ZCCHC17	RNASEH1	CD320
FAM156A	TMEM50B	CXCR6	STOML1	MGMT	ZNF688	CYP2R1	HNRNPD
RNASE11	IGHV5-78	KJ904180	DYRK2	PTPRE	PPP1R8	SAAL1	CEP76
S100A13	SMR3B	ZNF215	LDB3	C11orf74	RLN1	ASB3	CRTC3
SLAMF7	CD59	SLC35B3	KJ905695	HIST1H1C	CD164	IMPACT	TMED10
ELMOD2	LTA	SFR1	NCAPG2	IQCD	IL34	ARL4D	RRAS
GPER1	ARL2BP	CALB1	LARP4	EBP	KPNA6	METTL17	IQWD1
SMCP	LZTFL1	PNLIPRP1	LASP1	KLK3	SPG21	VPS72	MEST
B4GAT1	TSEN34	AKT1S1	IL2RB	BC030232.1	GAD1	AQP5	PPP1R14C
MAPKAPK3	PCK2	GNB3	VPS8	TMEM261	CHFR	ROPN1L	CHRAC1
ZNF22	STYX	ATG3	FHL2	GHITM	NXF1	APEX2	KJ900875

HMBS	LDHA	RBX1	GAPDHS	ANKRD29	MRPS24	SPX	C7orf25
CDKN2D	STRADB	PIP4K2A	CDC20	ARIH2	HOMER2	SLC22A15	RNFT2
RAN	SPAG9	MCM7	REPS1	PLA2G3	TRMT6	C19orf57	BC073758
SNRNP70	SLC39A7	HSBP1	SYNDIG1	BEND6	TMEM106B	SEC13	CXCL14
TMSB10	RAB22A	GPR87	GABRA2	SERTAD3	METTL6	MRRF	MRPL32
BC018766	POLE	EMC2	CNNM4	SNRPA	E2F6	SLC25A3	SLC7A7
ACTG1	TUBB3	GALK1	MXI1	NRP2	CT45A3	DUOXA1	ATP5L
GLIPR1L2	TIMM17B	GIT2	GCK	BNIP2	H1F0	RPL12	TRAK1
RAP2B	FNDC8	RFX5	UCHL5	EIF4EBP3	HIST1H2BN	OGT	KJ903630
PSMA4	HACL1	SUSD4	APOL2	CAPZB	PGF	WBSCR28	TMEM74
MALSU1	RDH11	ABHD6	RAC1	GNAI2	RPL29	CA2	KLRC4
PCBP4	ARL6IP1	FAM131C	AHCY	ENKUR	EFCAB2	KJ903261	LIPT1
AKR1B1	KDELR2	FKBP2	ING2	AC236656.3	KDM8	SNX8	ILVBL
CA7	CGB	SERPINA10	CCDC59	DGCR8	TMEM30A	NKAP	CD40
HAT1	ACTA1	P4HTM	MEOX2	GTPBP8	EPHX1	ENKD1	RPL36AL
YTHDF2	KRT18	CCNB1IP1	PTPN2	CEND1	EDN2	TIMM10B	ISCU
ARL1	UBE2E2	BRI3	GKAP1	MAPK12	CHP1	ACCS	CLDN1
ZNF223	ASB13	SUGP2	IL1A	ALX1	COX11	PAIP1	NINJ1
ELMOD1	CCDC109B	ECSIT	MYL1	PTBP2	NARF	DLD	PVRL3
ETV4	TRIM31	UBE2U	CDKN2B	TYK2	HIATL1	IGLL1	HOMER3
CCDC134	C6orf203	MRPL40	MEF2BNB	ACSS2	SULT1E1	POLR3C	HSDL2
ARHGEF16	KPNB1	ZNF273	DDX46	PSMD4	GNL1	COASY	SPIRE1
UCN2	SEMA4G	MSC	NR1H3	ARAF	SERBP1	AF116637	VPREB3
PLPPR2	TBC1D23	FIP1L1	CCDC127	RPRD1A	IGIP	MTRF1	SLC5A6
ACOX3	SNAPC5	ECHDC1	TMEM51	PECR	DGCR6L	CNBP	TMEM126B
TRAC	MS4A12	SDHAF3	MRPL33	TLCD1	ATF3	CHMP6	KIAA1598
RRAGA	RPH3AL	NMRAL1	NCS1	CA8	SKA2	ANKRD40	PEX11B
EURL	RRN3P1	UVSSA	NSMCE1	MORN1	MAOA	AGO2	STC2
SRSF6	DIRAS2	ZNF436	SPTB	THAP10	SUV420H1	IDI2	TEX2
DDIT3	PHKG2	IKBKB	HLA-DRB1	PLGRKT	HLA-DQB1	AP4B1	MYOZ1
SPSB1	EMILIN1	ATP13A1	ARF1	ATP6AP2	NDUFS4	ID3	DPYSL5
ZC4H2	C16orf74	HIST2H2BE	DACT3	LAMP3	C1orf111	CHID1	ZNF24
PGAM2	KCNIP3	MICU1	SOD1	APH1B	CCDC26	ZFPL1	LOH12CR1
PDCD6	FKBP9P1	PSMD12	HCK	GFRA1	HIST1H4A	DLX5	SEC61A1
COX6C	PTRH2	SGTA	CRNN	MRPS36	NEURL3	MOCOS	AKIP1
NRSN2	CLDN15	FGR	PPP3CC	CDCP1	ZNF501	ADPRHL2	SC5D
FBXW11	MB	ZMAT5	PRPF19	ATAD2	DUSP18	LRRC17	ANAPC10
ALDH6A1	COX7A1	VDAC1	XM_014342361.1	ARFIP2	MAD2L2	PHLDB1	SUN1
HLA-DQB2	KHK	ITGB1BP1	CD52	C1QTNF1	ETHE1	CASC4	RBFA
CD99L2	MINA	BC009297.1	CENPJ	COIL	BCAP31	STAU2	CCDC130
TMEM39B	PPA2	RPLP0	HPCAL4	CFAP53	STARD5	BAG6	WDR5
TRIM13	NKIRAS2	AKR1E2	NRAS	RPL6	MPLKIP	C5orf28	AFAP1L2
FBXO30	NFATC2IP	APOBEC3G	TMEM116	PNMA6A	ELOVL1	SEC61B	RAD1
HDDC2	ALDOC	FAM3D	KJ903749	KJ901790.1	MRPS26	BDNF	RSPH9

AK2	ENPP4	PATZ1	CYP2U1	ELAVL3	NUDT6	CLCF1	ALG5
SMIM3	PHF6	BTF3	KRR1	FCAR	KIR3DL1	PDGFRB	ST6GALNAC6
ZNF557	STX5	SSX2	BEST3	ODAM	UBE2V1	UBE2D3	IL2RG
EDEM3	MGC18216	GSTM4	TAGLN2	HPGDS	PORCN	DAG1	TSPAN3
TAF1B	CABYR	SLC35B1	MZB1	ZNF331	HSPBAP1	LCP1	PRELID1
NDUFB2	PRPF3	BT006963	CISH	WDR45B	CD48	GNG4	KCNK13
KDELR1	NDP	ASPRV1	RAB35	VIPR2	KCNMB2	MYL4	DHODH
ITLN1	ECE2	RGS13	CYB5R1	FARS2	SAFB2	ARG1	FAM92A1
THAP2	RAD18	SPIN2B	DDC	C17orf62	INPP5K	TESC	LIN7B
WISP2	C5orf46	LAT	N4BP2L1	SQSTM1	NME1	MBTPS1	ELP6
TIPIN	PHF21B	WBP2NL	BCL2L14	RTF1	IRF2	SF3B2	LOC105372481
MCC	CYP39A1	CELA3B	TNNC2	TSTA3	TRMO	SYNGR2	MAGEA8
EIF4E2	GABRA1	CALML3	TRIM48	TIMM17A	ZNF706	CA12	TMEM14C
CNOT2	PSG1	KLHL14	TADA3	CCAR2	SULF2	EGFL7	EIF2A
TMEM39A	TBCC	COQ6	RPP40	ACSM5	UNC45A	SARS	FUT2
ADSL	DPAGT1	MOB3B	MGC35361	TMED5	ALMS1P	MOBP	RNF183
SPEG	KJ904785	CXCL10	RPS6KA2	HACD1	MOXD1	FABP7	CDC37L1
PRKRIP1	SCAMP1	TSPAN5	SLC25A6	CCDC93	KJ901232	ICOS	DHRS4
TUBB6	RNF181	TUBB2A	SDC4	MKRN1	DOCK2	KJ903819	LMNB1
GRPEL1	CDK7	PI4KAP2	MPV17	MFSD2A	TSSK1B	UNKL	TMEM231
SLC25A46	SH2B1	TBRG4	TRAPPC2L	HBG2	CA5B	COMMD8	METTL9
CLCC1	FTL	MARS	PCNA	TMEM241	SNN	ATXN7L1	PIGC
FANCI	EFTUD2	SULF1	CCNH	KRT14	VPREB1	FAM122A	EDF1
PANX1	HEXIM2	CCER1	MRPL24	TXNDC17	NUPL2	SDF2	POLR2H
SNRNP25	DHCR7	CYB5A	PQLC1	CDO1	NG_008285.1	GIMAP2	HVCN1
C8orf44	OPA3	RRAS2	SSTR2	CDC34	SELENBP1	SIRPG	FAM83F
ARL8A	TNIP1	ANKRD13C	WDR70	MARC2	CYB5R4	RAD51AP1	HLA-DOB
RBPMS	EIF3G	HADH	SLC19A1	YWHAG	LRR1	EXOSC1	SAA2
KJ904366	PROCR	TEX37	MS4A5	SYT17	DGKE	XPO5	APEX1
ZFYVE21	CLDN2	FAM81A	RBM33	ZNF35	ACAD10	QPRT	GLUL
CXXC5	CPA3	HPX	ART3	SRD5A3	B9D1	EEF1B2	ACTB
MP68	ATG13	MANSC1	EMC10	H2AFV	DDX56	MAP3K3	UHMK1
MRS2	PPP1R3C	C18orf21	SYNPR	S100A6	ACOT9	RWDD2B	MAD2L1
ELL2	C11orf16	IDH3A	DDI2	RPP30	SPARC	GLA	ZC2HC1C
RUNX1T1	NGDN	LYZ	C10orf91	DNAJB4	NUP133	KIAA0907	SEC22B
CYP2C8	FOXRED1	SFXN2	UBE2F	RAB7B	HMG20A	MMAB	NREP
ADM	ORC4	HSD17B6	STAP1	RBMX	AAR2	PDAP1	IFITM1
SF3B3	CD79B	TMEM61	NUDCD3	SIGLEC7	C11orf45	PTRF	ATP6V1G1
PPP1R17	BMX	ECI2	PPME1	RAB37	BAX	ELL3	CASP3
ZSCAN9	PBLD	HEBP1	TNFRSF9	NOTCH2NL	PIGF	FKBP11	VAMP4
CA3	EIF3J	EFCAB11	RNF7	LYRM4	POLD2	COPS7A	TCEB1
KJ901923	PLEKHA6	WRB	TMC6	IP6K1	EIF6	NAMPT	TAGAP
LAS1L	RNF114	HAUS8	RAB24	MGST2	GZMA	PQLC2	UBAC1
SLC6A6	METTL7A	LSM4	NG_034163.1	CCDC108	CMAS	KJ902521	SURF2

TDGF1	ENTPD1	MS4A15	BCKDK	SAT1	TMEM40	STXBP6	TGIF2
CIDEC	KJ903904	FLOT2	GNA12	LINC00312	TTC23	STARD3NL	POLL
COL6A2	TOMM20	RRP9	GP9	RPL23	NFIB	LGALS12	FGFBP2
KPTN	HEATR9	HDDC3	C10orf111	PRKD2	IMPA1	ACP5	MRPS16
GCHFR	FAM172A	HAVCR1	HAUS2	TMBIM1	PSMA1	HEXIM1	PMFBP1
SNED1	MRPS21	NUF2	TGM4	TRIAP1	APMAP	ASMT	YV007
VPS37A	HRAS	DCI	CMTM5	APOC4	KJ901001	TMEM263	CLDN3
LAD1	RPS13	ZBTB7A	NXPE3	ALKBH8	CCL3L1	HSH2D	BC011600.2
CTNNA3	TEKT4	ZMYM5	TCEAL3	IL18	DDX50	TGFBI	EIF3C
CAPZA1	VMP1	SGCD	HHEX	FOXO3	LETM2	CTHRC1	EPB41L4A
YWHAZ	AKAP1	CCL2	RSPH14	SKA3	PPCDC	CYP46A1	KRCC1
N4BP2L2	C1orf21	ITGB3BP	IRF4	CD300LB	GMNN	WDR6	TMEM177
HIRIP3	SNX1	YPEL5	MYO1D	SALL2	HES4	PSMG3	ARMC10
CAPN6	PKM	ACVR1C	RPL19	TIGAR	LGI1	MFN2	PIGT
ACTRT3	INTS4	SETMAR	TUSC3	RPE	EIF1AY	B9D2	SNCB
CLINT1	ACAT2	FUS	RPS20	STAM	MCPH1	CFAP20	P2RY6
GJB2	RPS7	TALDO1	GBP2	SDPR	SLC25A38	MKRN2	TMED2
BTF3L4	GIMAP7	SHD	GLYATL1	ZNF76	C21orf59	SLC25A23	HOXA5
ARL3	CXCL16	RSAD2	TM4SF18	RBMS3	LARP1	BC030991.1	AK1
PRPS1	SLC1A7	HMGB1	TIPRL	DLX3	HS2ST1	MUT	TMEM183A
TAF11	PI3	TBC1D20	RABGGTA	PEF1	TTC39A	DNAJB6	ARG2
TMA7	RAB10	SPANXB1	MLC1	KLHL22	C19orf12	MMTAG2	SAR1B
ARL2	TACR1	RPS15	RPL8	SP110	EVA1A	BECN1	HIST2H3A
ING4	PSMB10	MAGEB2	ZDHHC6	SULT1C2	GPBP1	FAF1	IGF2
YKT6	VANGL1	JADE2	AQP9	EEF1DP3	ABCB9	CLDN12	KHDC1
TRIM39	SFTPA1	NIT2	MRPS22	TNFRSF21	RNF208	GGCX	KPNA3
USP53	RPA1	RPL10L	TGM2	GSTM3	TPM3	HNRNPR	RPL24
ZNF174	IFIT5	MPPED2	LYPLAL1	CHST11	COQ4	SNX10	SAMD3
KJ900956	ARHGAP29	PTPN11	THOC5	C9orf9	NXNL2	MRPL44	DCPS
DTD2	C21orf33	CGRRF1	FHL5	IDO1	B3GAT3	HSD3B7	IFT20
UBE2R2	RSG1	APBB3	HDAC1	CAP1	UBE2O	CHAC2	CHI3L2
SERINC2	XPOT	PIK3C3	DUSP11	ABLIM3	GJB3	CD200	NFIC
RECQL5	LYRM2	CREB1	SNAP29	APOA1	TCAP	ACO2	PPP3R1
SEMG1	PSMA7	CITED1	TMOD1	DHFR	ZNF3	PRELID3B	ILF2
IK	SLC22A23	TXNDC9	KJ902780	RBM8A	TEX101	PDXK	ERRFI1
PHF23	PYHIN1	PSG6	UAP1	RNF32	AHCYL1	RNF167	ZNF266
LAMP1	UBE2W	HOMER1	TRA@	WDYHV1	TMEM9	CDCA3	FAM118B
SHMT1	GZMH	NMNAT3	KJ901928	PDZK1IP1	WDFY2	DPT	C12orf10
C3orf22	RASL11B	GNAI3	RPL18	HAX1	MAPK1	AP1AR	AARS
IFI16	L2HGDH	GAPVD1	SAYSD1	IPPK	NFKBID	OXNAD1	HSD17B8
ASL	HINFP	NMUR2	PHLDA1	APOH	LINC01558	PGRMC1	NSMCE2
PITPNB	SGK1	UCK1	CNPY2	DYNLT3	GAPDH	ZFP69B	PQBP1
SLC25A31	HYLS1	TAPBPL	PSMB4	HLA-DRB5	INTS12	RSL1D1	LAMP2
DHRS7B	YPEL3	ACY3	HMGB2	TOMM7	SCML1	BMPR1A	ZBTB37

NDUFV2	DFFA	FN3KRP	TMEM147	S100A11	RGS18	ZNF37A	C16orf58
PHF10	PFDN1	LMAN2	SLAMF1	ALOX5AP	ATP5O	BIN3	MRPL51
TSSC1	CD2BP2	KV205	RNF175	PARK7	NRG4	SEC24C	HOXB6
BTG4	CHMP4B	CDK2AP2	DGUOK	DCLK1	DUSP3	PTCD2	UBD
PLPPR1	PROSC	HNF1B	IL24	MARCH5	SRP19	SLC1A6	HBZ
HBA1	CDC23	WDR25	PPARD	HMGN3	RABEPK	LIN28A	RP11-998D10.4
NKG7	TMEM159	ACTR10	ATF2	PFKM	CDK16	CSNK1G2	GBE1
RABGAP1L	RWDD1	HNRNPA0	CD7	TMEM14A	TMEM161A	RPL21	SMU1
APP	RSRC2	IGBP1	RPL34	LMO2	ADH1B	KJ903456	NAGA
HIST2H2AA3	ARF6	RAB9A	KCTD15	DDX17	CD96	TSPAN17	TXNL4B
PEX3	ZKSCAN3	IFI30	STRA6	FAM122C	ACTRT1	BC025996.2	NOL6
DHDDS	TMEM59	KRT8	GFOD2	NDUFC2	SCAPER	FRMD5	NDUFS1
CASS4	COX20	DNAJC30	KCNRG	GTSF1	CDK9	CAMK1G	KJ902474
TMTC4	NFS1	DCUN1D1	AASDH	DPP4	YIPF1	PTN	PDZK1
C1orf87	RP11-58H20.3	FUBP3	SNUPN	RBBP4	ZNF346	HG497585.1	SSR2
SSX5	KJ902823	PAICS	RAB2B	OSGEPL1	IFI35	NDUFB7	BHMT2
LYPD1	RITA1	RTN3	ASCC1	HPGD	CD36	CTSK	PANK1
TSPAN32	UTP3	GOLM1	ECT2	DCTN3	PLPP5	C2	VIM
ZFC3H1	TMSB4Y	YIPF3	RHOH	LGALS2	MMP28	KRAS	SRI
MYL3	FIBP	MPZL2	METTL21B	RTP4	SURF4	HPD	SPTLC3
ANP32A	TMEM176A	DDX18	NASP	MRPL15	ZNF330	F12	CHMP1B
TMEM254	CHMP4C	SERPINB5	BOLL	MYL12B	NME3	TK1	PDGFRA
ENOPH1	PSME1	MRPL53	WIF1	SGCA	ZFP36	CASP7	APOBEC3D
BNIP3	SH3GLB2	NDUFB6	ASPH	ITFG1	XKR8	MED27	SCGB1A1
RPL35	UQCRH	AKIRIN2	ID2	DKKL1	SERTM1	AGPAT3	CREM
FGF12	SCAMP2	ZNF593	RNASE6	SPDEF	MED8	TMEM187	PPP1R7
TSR2	RPL31	RPS26P19	CADPS	DCLRE1C	CXCL3	C9orf78	C16orf70
U2AF1	GSTO1	GATA3	AHSA1	CNTD1	CD177	RIOK2	CFP
TMEM140	LBX2-AS1	SENP8	CLPS	MIA	PIP4K2C	PAK6	RBM38
POP5	RPUSD4	MID2	ORMDL2	PSG4	ARHGDIA	BBS4	TEAD3
FANCD2OS	SP2	ANKDD1A	RIBC2	ARRB1	PLEKHA8	LMOD1	ZDHHC16
HSPBP1	AKR1C1	GYG2	SRGN	LINC00526	TPRKB	GTF2I	CKMT1A
MAP3K6	SDR39U1	BATF2	BBS10	HOGA1	GFAP	GALT	RUNDC3A
GADD45A	ATG101	SULT1B1	KLRD1	MRE11A	RCAN1	MPST	BCS1L
EI24	UBQLN4	FXR1	CNOT6	RP11-287D1.4	KDELC1	EDNRA	CLCN2
CLDN4	TFF3	GOLT1B	LSM14A	CD84	FAAP24	TMLHE	KJ900937
GLUD2	PCED1B	KIF1B	HERPUD1	SLC29A1	MVD	NFKBIB	DHX30
GUCY1A3	EQTN	CHCHD6	CA6	TNFRSF14	RABL2B	LPAR2	PRPSAP1
TM2D2	ENO2	BIN3-IT1	TOMM6	CDC6	MORN4	GTF2H3	P3H2
GSDMD	SNX17	FKBP1A	RRP1	RPL4	HEMK1	DUSP22	PDCD4
HAMP	MIF4GD	ATAT1	STMN2	MKNK1	PICK1	MRI	ALG8
FOSL2	SPEF1	POLR3K	GFM2	RCC1	RPS16	KJ903487	HRSP12
CD300LF	BCAS2	MED7	THUMPD3	VKORC1	PCYT2	NUTF2	CCK
SS18L1	TMEM86B	PRR15L	NCAPG	RPS2	BRSK2	PTPRR	CYB5D2

ZNF641	FXYD2	EXO5	BBOX1	UEVLD	BCL2L13	VSTM2A	EZR
NDUFS7	CTRB1	RPS27	KJ901471	VCP	LOC100132686	WDR18	DCTN6
LHFP	S100A16	C1orf109	MTUS1	PDIK1L	CCDC86	PSMC2	FGA
ERAL1	ABCF3	LCAT	METTL7B	UBR7	DNAJC7	FUBP1	TFG
CMAHP	TMEM101	SLC35A3	ELMO1	TMUB2	DCTPP1	LMNA	RAD51B
DAO	SMN1	NUP50	C20orf141	C8orf33	CRH	RBP5	CCL21
OXSR1	PLPP2	GPSM3	FAS	GLI4	ASF1A	AK024408.1	LMBRD1
SMAD5	ASNS	NTS	TMEM217	LINC00846	BCAP29	MPHOSPH6	MRPL39
SFTPC	DYNLL2	SUCLG1	PABPC1	TBCEL	CA9	CTTNBP2NL	MZT2A
LRAT	DBN1	ATP5E	HN1	DPEP2	GPRIN2	CFL1	C10orf54
FKBPL	KJ903532	ETS1	FASLG	PLEK	CSPP1	NUDT16L1	FAIM2
NNMT	CENPM	PEX19	KJ904393.1	RBM7	TRPV5	THNSL1	HTN3
SLC22A5	MND1	KIF26A	SNX5	ESYT2	C1orf43	TMED6	ENO3
ELAC1	ZKSCAN7	FOXA3	ARHGEF5	PLBD1	ACKR1	POLK	HM13
EMD	NAPG	NDUFB4	PSMC1	LRRC18	C17orf78	IRF6	PPBP
LARS2	AGFG2	METTL21A	TMEM100	HK2	DUSP4	ACP1	LRRC29
PHPT1	ARL5B	TAMM41	GSTP1	HSD11B1	SMAP	NIF3L1	GPD1L
DTD1	MGST3	XAB2	ARL4A	DDX49	AC255553.1	BC007528.1	NPR3
DGCR14	CRTAC1	DRAM1	TFB1M	XR_678317.1	C19orf53	MT1F	AOPEP
FEM1C	MAT2B	ERCC8	DDX1	SH3BGRL	SEMA4A	INTS3	SURF1
ASB17	SLC25A45	TMEM25	WFDC2	KIAA1109	CHRM5	SSBP1	FRMD8
FURIN	PARS2	ZNF467	SLC10A7	GNAQ	TUBA3E	DPYSL2	MLLT3
DDX4	GBA3	FAM27L	ZNF471	ELF3	ZNF485	RARRES2	PRCC
FNDC3B	BRF1	EPS8L3	ARMCX1	THOC1	BMI1	UROD	ZNF610
DCDC2	EBF1	SPNS3	MTMR6	NAALADL2	MAGEE1	OSBPL5	TRABD
EML1	VCAN	CCDC83	CETN1	FOXP2	DYNLT1	CBX4	EIF4A2
AUH	SMAD1	TP53TG1	CCDC124	CD300A	MEN1	OR1E1	GMPR
BHMT	MAK	MEF2D	ANKRD13D	C6orf89	GAS2L3	OR51I1	MAPK9
SLC13A3	DNAJC12	C7orf62	FBXO38	BAG5	PTPN4	DDX55	TRBC2
RHEB	CHMP5	APOC1	SNAPIN	TFPT	SCO1	ABHD17A	PRR15
PSTPIP2	CDK15	PRKAR1A	LY86	MRAP2	CCSER2	RASL10B	ING5
C11orf84	TMEM209	ZFAND4	IRF9	EPOR	LRP12	C6orf118	RPAP2
KJ904264	RPP25L	GNGT1	GINS4	FAM21D	SPATA6L	KRT20	APTX
PIGV	GNG5	PRR13	CBLC	KBTBD7	RFFL	H2AFY	SLC37A3
RSPH3	TNS1	ELL	PPA2	OR5V1	SLC22A13	THBS4	CIZ1
ACRBP	PPIP5K1	CLVS1	PELI1	BC047307	PAPSS1	USP44	ASB9
PYCR2	ATP6V0B	MARK3	SFXN1	CATSPER1	EFEMP2	BCAT1	CRACR2B
TGFBR2	ELAVL4	SPACA4	DNAH14	PEMT	ACOT13	ARHGAP25	DIXDC1
PGBD3	ZNF765	WDR53	RARB	CES3	GRB2	ACOX2	YARS2
TRAF2	MRPL16	ZNF738	TMEM203	EPCAM	OSMR	SIRPB1	CU689359
C19orf18	RTCB	OR6B2	GPR63	UBE3A	TNXB	LOC554223	SLC39A6
NPL	ITGB3BP	CEBPE	PI16	FO680690.4	HP	CXCR1	PLPP1
TAC3	RFC3	CHGB	SCCPDH	RAD54B	EWSR1	ZFYVE19	SMOC1
TGDS	CNPPD1	CPN1	CPA5	CCDC7	TMEM150A	FBXO25	OR2M3

FAM159A	IL17RB	CMC1	NCR3	OR2B3	HGSNAT	SEL1L3	FOXP4
RNF213	ALDH3B2	INVS	CLP1	SORBS3	TUBB1	RSPO3	GNAT2
LINS1	WDR61	MBOAT2	TFAP2A	NT5DC2	WDR19	PEPD	HNRNPH3
OR4N5	PKIB	MYC	GTF2IRD2	MRPL43	OR1J2	CLMP	HDAC4
PRKCA	C9orf131	C6orf136	SLC30A2	NELFB	SERPINF2	TMEM106C	TBCA
H3F3A	SLC25A44	RFC5	ISG20	FAM177A1	OR14I1	OR2B6	DNAJB2
CCDC36	TTC30A	ZNF134	C6orf141	ORAOV1	VAT1L	YP002	SOCS3
FXN	PNMT	HIPK1	SSH1	INTS7	KIAA1683	SPP1	SULT1A3
SNAP23	SS18L2	FAM136A	CDC5L	CENPI	PGC	TSPAN12	PCYT1B
FCGR1A	GTSF1L	COMMD2	TROVE2	MRPL41	FPR3	SLC2A14	ZNF790
ZNF695	ENTPD1	CTSA	SNAP91	CRY2	LRFN5	CALM1	KJ901225
BC017869.1	RNF148	MARVELD2	KJ902497	COPZ1	ANXA11	ATG12	KLRC1
HFE2	PTBP1	OR2J2	LCMT1	CSNK2A1	ZBTB25	WASF2	CYB561A3
GAS2	TMCC1	OR5J2	MRGPRX2	CHRNB1	ECM1	MSN	EID3
C1orf101	PCYT1A	TEKT2	C10orf2	CDK5RAP3	NDST1	VGLL1	MED21
SLC39A1	NDUFB11	WWC1	RYBP	NCAPH2	OR5D14	SESN3	ZNF16
GLE1	GPAT4	SEPHS1	APOA1BP	SLC25A5	L3MBTL1	MAFK	HACD2
APOBEC2	SIMC1	RNPEP	PSG2	PLTP	BNIPL	ACY1	FBLN5
THAP11	B4GALT4	SEC61A2	TEX13A	ENPP6	SLC41A3	PPP1R3B	APOL6
DOLPP1	GPR21	PTMA	RAP2C	DSCR9	SIRT7	HACD3	MRPS31
AGTR2	RPAP3	CSRP3	CDCA5	FAM114A2	DESI1	SPINK6	DMRTC1B
FAM53B	PER3	PPIE	UBE2C	PLRG1	RBM14	CSH1	ISOC2
GHSR	BTN2A1	ACAA2	IFT52	TRIM69	SPDYA	ANXA5	ALB
SLC44A1	OPHN1	XCR1	OSBPL6	REC8	CXorf40B	PIGK	SLITRK4
DMRTC2	GRIA4	CPSF7	RHPN2	LIG3	SLC25A18	ODF2L	CARHSP1
TULP2	PCP2	TEF	ADAM22	ADAP1	STX17	TMEM17	KJ900936
FADD	SLC2A2	PIK3R5	TBCD	PRLHR	SEPT4	IL18RAP	SHOC2
FBXO42	ALS2CR11	ICAM1	WNT2	KRT15	FHL1	FGD6	SERPINB2
RPN2	KIR2DL3	GLT8D2	SCLY	KLF15	TOR1AIP1	TIGD4	REM1
RP2	MAP4	OR5P2	MED31	CARD8	TRIM27	CLN3	CCDC94
QPCTL	COCH	PHLDA2	NAT2	EMC3	IFI27	MLST8	ALG13
DOK5	PIP5KL1	JAZF1	TRIM23	OR8B4	NG_012856.2	SLC14A1	KLHL36
VPS52	MAZ	ATP11B	EHMT1	GRIN2C	GIMAP6	TMEM54	PRSS58
GIPC2	GTF3C3	MAP3K7	SIRT5	TMEM205	VPS28	ENTPD3	CDC42EP4
CYB5R2	PMP2	MAGEA2	TWF1	PRELP	HINT2	RALA	GABRA4
FAM228A	ZNF577	SEPP1	KPNA2	KJ900957	HRH2	ZNF131	ABCC4
CCDC185	CACFD1	NG_013357.1	NATD1	NOV	MTG2	PLEKHB2	FUCA1
PDE9A	SMIM2	FKBP7	ZNF277	FAM13C	OR3A4P	ADK	TDO2
CYP4A11	LOXHD1	XRCC3	OAS2	OR5B3	AXL	PTPRO	DPF2
ZNF564	CLCA2	TPRA1	ERICH6	ARL6	ASGR1	PNPO	CRISPLD2
MRPL20	MRPL49	CD302	CDC20B	SLC2A5	PRC1	PRKCG	TRDMT1
SYVN1	RHOBTB2	RBBP5	GOT1	ZFYVE28	STRADA	MAP4K2	UBE2M
RNF115	ATRAID	FAM102A	SLC25A42	DIP2A	ATG4C	SFXN3	ATG7
STEAP1	NARFL	TNNT2	RNF133	ACAD8	MSLN	CLYBL	PTER

MGAT1	SEMA3D	CEP57L1	ATP6V1B2	FMR1	CALHM3	OXER1	KIAA1143
RGS19	TPM2	RMI1	L3MBTL3	MAP3K11	MAGEA12	RNF151	PKIA
CHD9	NIPA2	SPHK1	RFC2	DNAJA3	LOC57228	HIGD1B	SPAG7
SMAD2	PARP16	OR2T35	A4GALT	DDB2	FAM134C	NRM	AGPAT5
KLHL7	OR51D1	AAGAB	VRK3	MAP3K8	EPB41L5	CSNK1A1L	BTRC
NID2	HS1BP3	MAPKAPK5	AURKA	SENP5	SUV39H2	ITPK1	C11orf73
RBCK1	ZNF493	CD9	RRP15	SERPINB9	EXOSC4	CALML5	CD86
TSSK2	UGCG	BC041668.1	CYB561D2	EDRF1	TSSC4	ARMCX5	RPL32
DLX1	TNIK	PSMD2	BRIX1	DLAT	KIAA1586	CMTR2	PRIM1
CATSPER2	THTPA	RPL36	SCAMP4	P2RY2	CST9L	KCTD13	PEX12
AADAC	NOP16	CCDC43	ADAD1	SSSCA1	LCOR	SLC17A7	KJ904273
MRPS17	P2RX5	ZNF280A	EME1	AK7	TAX1BP1	MIER1	U633C
RBBP6	AK096729.1	METAP2	TMSB15A	CSTA	NUDT21	ACYP2	TCEANC
OR2T10	TNK2	LRRC6	ZNF26	RCHY1	TXNDC5	RDX	OR12D3
NCOA5	MAPK6	GNL2	PRKAA1	ALKBH1	C16orf62	SYT5	MEIS2
FAM234A	SAMSN1	TSNAX	TSG101	SLC35A5	PRAM1	ZNF444	CDR2
RBM34	TUBE1	PFKFB3	VASH2	P2RY8	FAM72B	OR4K2	GBA
AVPI1	C16orf45	SRSF3	NEK2	MYO1A	HOOK3	AIMP1	XPNPEP3
NOL10	ICAM4	ENDOD1	KJ902931	KCNG1	ANP32E	PAIP2	RABGGTB
MRPL35	SCRN3	CRADD	CDK18	CFAP52	GPR151	EAF1	CYTH1
SLC4A4	DZIP1L	PHOX2A	HSD17B2	ARPIN	BCDIN3D	UPP2	DIAPH3
CLCN6	TTC39B	WDR5B	DDIAS	FABP1	SIPA1L2	CYP20A1	TMEM45B
ZNF707	SQRDL	ERLIN1	TRIM44	ZZZ3	ANKMY2	STX2	TMEM120A
NPHP4	OR51G1	DAD1	CBX3	ARPC5	SIRT6	PSRC1	MAPK8
DDX11	TEX2	IKZF5	EZH1	OSGIN2	DCAF7	CCDC87	CGA
TSC22D1	AKR1C4	NHP2	KJ904199	CD19	OR52E6	POLR2D	CIDEB
TLDC1	SSBP4	FOLR2	SEC63	ATP9B	IL12A	KJ901467	CACUL1
SPZ1	OVOL2	HCCS	PHLDA3	FAM162A	TP53I3	ARL15	GUCA1A
GPKOW	ABCF2	OR4K5	TSSK3	LEF1	TMC5	PLEKHA4	NEK6
BC054893.1	GTDC1	DCUN1D2	SLC39A11	SGIP1	HMG20B	CHRNA7	AP5S1
HDHD2	WDR54	PTK2	KJ903543	HAO2	ST7	USP45	DNAJB5
TTLL1	KIF22	TOM1L1	RAPSN	THADA	SYT4	MPND	NAGLT1
KATNA1	PTGDR	RPAIN	OR6Q1	RPL37	LACTB2	TNFRSF17	ALPK1
COG2	APOPT1	KLHL13	NDRG1	TEAD1	EPB41L2	CFAP43	YIF1A
PPP1R2	EFHC2	ABCB7	SEPT6	C8orf48	ZFP82	KJ901782	MDP1
DHFRL1	C11orf53	CCDC78	UFSP2	C7orf31	SMIM11A	RBMY1A1	DRD1
TMEM208	SDHA	MISP	TNNC1	CSAG2	OCIAD2	ATF5	KJ903230
CSF2RA	CCDC77	TMSB15B	SPRYD4	CDC25B	DES	UBE3C	OR10H2
SERPINA12	ZBTB44	SPATS2	NPRL2	ZSCAN12	SSUH2	OR56B4	UPF3A
ZC3H18	ZER1	SRPRA	TBL3	PTPN18	FCHSD1	TTC9C	ARSJ
TMEM256	TRMT61A	DLK1	MNAT1	CEP72	TOX	E2F2	CHMP2A
CCDC146	ALDH4A1	OR5M1	SNX15	ERP44	SMOC2	APLF	SPATA9
AURKB	CCSER1	CA4	CCDC107	NEK3	GPC3	RFX6	LIAS
TBC1D10C	ACVRL1	B3GALT2	PLD3	SETD4	RHEBL1	DPH3	PANK2

WBSCR22	PSMG1	MESDC2	C12orf54	CDK5R1	TMX4	VASP	CNIH2
PDZD9	CCDC60	OR4D11	GYG1	FANCL	APCDD1	FAM161B	BET1L
HCAR3	KCNK5	ZAP70	PHF20L1	PAPOLA	NUDT16	SHFM1	CKS1B
ZDHHC19	MCM9	MNS1	LOC105372824	PYROXD1	BC019348	CDIP1	LRRC43
RARS	OR10H1	DNASE2	KRT36	SPNS1	PTBP3	ARHGEF10	CROT
OR10W1	DHRS9	SMARCAL1	PAPSS2	EIF4ENIF1	BC048416.1	KIAA0408	HNRNPA1
TRPT1	SLCO4A1	CDK4	RFX3	RUSC1	ZNF680	LPAR1	MIC13
PSMA2	ATP5J	ACTR3	SCG2	CHRM3	SPAG11B	RUNDC3B	HCRTR2
TGFBR2	OR4D10	PRKAA2	DBF4	ZMYND11	TAF6L	TPI1	COX16
MED10	CIAPIN1	GNB1	MINOS1-NBL1	ISCA2	SERHL2	OR9Q1	NPY2R
HES1	TTC30B	IMMP1L	ZNF25	OXCT2	DERL3	ALCAM	CENPV
PRSS35	C2orf27A	SLC27A2	LRRC49	PNCK	ETV6	MGC24103	HELLS
COMT	THUMPD1	GNAS	GRAMD3	TMEM171	MTUS2	AADAT	COPS2
RASSF9	CACNB3	IL33	ZRANB2	PPP1R21	OR6Y1	RXFP3	ELAVL1
MT1X	ZMAT2	OR6M1	CT55	RNF111	ZNF7	ANKRD27	PBK
CBARP	PSMB8	BZW1	TSC22D4	PHC2	TGFB1I1	ZNF165	NXPH1
PEG10	SMPD1	OR12D2	OR14J1	ZNF691	ZNF644	OR2C1	CDCA8
ACAN	SKIL	ABI2	TMEM175	EVL	NANP	LRRFIP1	PIM2
BAT2L	VPS25	EIF5A2	IGHA1	OR2T4	OR1Q1	TANGO2	NR1D2
PRKCH	RNASEH2C	MSRA	EPPIN	AP3M2	TBC1D16	PITRM1	GSTA1
PRRT2	TCEANC2	GCLM	F7	POR	C3orf20	CDK1	MPG
GNG2	TAX1BP3	SYPL1	C14orf119	PLA2G2D	ACYP1	WT1	RPS19BP1
LVRN	SOCS5	SCFD1	PTGR2	CPEB1	F2	PPM1H	ARHGAP15
ZNF169	FYCO1	MFSD6L	SLC44A2	ZG16	DNAJC10	SLC25A16	MTFMT
ZNF222	ADORA3	RAMP2	PDHX	GNPTAB	CDIPT	GPX8	GPR161
SCIN	SOX7	ZNF410	TOM1	STAU1	HSD17B11	KANSL2	SLC34A1
OR4P4	PLAGL2	GLYCTK	ZDHHC7	CDH3	EPHX4	F2R	MYOC
GAR1	ALDH2	SUSD3	KJ901275	TCTEX1D2	MORF4L1	MRPL27	TPT1
TMEM26	GALNT14	OR56B1	BC001284.1	FAM71A	RNF103	RPS6KB1	GEMIN8
TMEM81	KJ904395	LYPD5	RHBDD1	DUSP19	ADH6	CEP97	SEC22C
TUBGCP3	ZNF397	MFAP3	KLHDC2	IFI44	RNASEH2B	IREB2	ACTL9
ANKMY1	PPP2R5B	UBXN10	MICB	DEFB1	OR5H1	WBP5	POMP
NR2C2AP	PODN	KJ904298	ZNF281	SENP1	NID1	MARCH9	COX6A1
ROBO3	MXD4	NDUFV3	POU5F2	MKS1	TCEA2	ADAT1	LARP5
PDLIM5	ARMC7	SLC22A11	GPR119	HSF2BP	ZNF670	GART	ZNF20
SLC35A2	USF1	MTMR14	MGC4836	KRT4	FTSJ3	CDKL5	USP46
GDA	SHCBP1	TRIM52	C11orf65	TRIM41	MT3	MTPN	SNRPN
EVI5L	PPAT	COX6A2	CLEC18C	HSD3B1	DAAM2	TMEM9B	CTNNA1
C1orf210	TMEM144	CCDC65	GUCY1B3	LUC7L3	KCNAB2	GC	PDE4D
KIAA0391	TCF12	SLIRP	RIPK3	MTM1	MAB21L1	GORASP2	KCNJ15
ARRDC3	BUD13	DDO	FKBP5	SLC7A5	RIMKLA	OR8K1	LOC100128510
PDIA6	KRTCAP2	CERS2	S1PR3	PPARG	FAM84A	TRIP10	FDXR
CXCL5	CHRNA1	TANK	DBNDD2	ARL11	IGK	PIAS2	OR4B1
PIK3CB	FBXO4	GRID1	ARNT2	CLIC5	TRMT11	MSH5	OVOL1

ESCO1	DPP10	PTP4A2	TTPA	KIF26B	FAM168A	SIAH1	RAP1B
SRP9	PFDN4	TRDC	EXOSC5	TRUB1	TMEFF1	ATP5H	SYT16
RCBTB2	IL18BP	KIAA0930	ZNF83	SCNN1G	DNM1P34	DOC2A	WDR48
PMCH	RHOBTB3	CCDC57	TMEM44	TFF1	SNRPE	GDF10	OLFM2
EYA2	BT007332	TSPAN2	TEX26	IL22RA1	OR11G2	HSD17B7	TRNT1
WIPI1	SEC31A	MARCH3	CTSV	FIGNL1	OR4M2	FASTK	PIGZ
SPATA16	NBPF3	FAM186B	LAS2	NPY5R	TLX2	LYSMD2	GDPD1
WBP2	SNRPD1	GPS2	CYP2C9	ZNF581	YV020	C2orf42	OR4X1
ABR	ANGPTL5	LTA4H	RUFY3	PFKP	LRTM1	BCL10	APBB1IP
LRRC8B	PYCR1	TMEM86A	OR4D6	EXOSC10	SLFN5	BFAR	UBE2Q2
WDR4	ZSWIM2	MC1R	MRPL38	CCDC112	PRR30	GABPA	NAP1L3
TMEM110	ST3GAL2	CCL14	OR2T8	CLUHP3	AP4S1	DKC1	P2RY1
FOS	ACAP2	FBXL4	AMY2A	TET3	PNOC	FXYD1	CCL11
CRACR2A	FITM2	DDX11L2	TIMM13	TBC1D22A	GBP1	OR10J1	DYNC2LI1
DLC1	FAN1	SLA2	NANS	CCDC138	ZNF280C	OR2K2	CBR4
EPHB3	CAPN1	ASB8	CFAP47	TYW1	CCDC71	CHEK2	SNX25
ANKRA2	EPAS1	HHIPL2	SMPDL3B	CPSF4	PFN2	EPYC	GNA14
RASSF8	HRASLS	ERI2	C17orf75	CNKSR3	XM_008977733.2	FAM26D	ADORA2A
ADCK1	NFATC4	KJ903211	SORBS1	USP1	IQCB1	MYEOV	RGS1
BNIP1	PAGE4	FOLR1	CFHR2	CTDNEP1	MRPL42	GNAZ	WNK1
BAP18	IFIH1	FAM114A1	MRPS9	MED1	XR_001337930.1	ZEB2	ADRB2
SLC32A1	SLC35F6	PARN	SUPV3L1	NIPBL	GON7	SLC6A13	FAM105A
SH3BP5L	GPR17	ZNF563	BT007180	S100P	CUEDC2	ZNF296	ELMO3
OR6K2	ATF4	HYAL3	TAF7L	DRC3	STOML2	SEPSECS	SLC15A2
OR1N2	MIS18A	WEE1	ORC3	MTO1	CD4	DSE	LRRC27
TMPRSS6	GTF2E2	ADAMTS6	ACTL8	NCF2	STOM	DDHD2	COPS3
STIM1	MOAP1	ZNF526	PRPH	ZCCHC11	OR8D4	HPRT1	CENPN
VPS18	KNG1	ADAM12	VWA5A	FANCG	CEP85	CAMKK1	KIAA1257
SPACA1	CD82	HINT1	ARPC5L	TRUB2	GEMIN6	TMEM248	LILRA2
KRT5	OR10G6	KLHDC3	SLC5A12	CD6	B2M	GJB7	PHYHD1
TPM1	GJB6	FAM35A	RAB1B	OR2Y1	C17orf82	CRYZ	CBLB
INIP	BIN1	GNPDA2	DRG2	ADCK2	NADK	TYMS	GDF3
PCYOX1L	INTU	SERPIND1	LAMP5	ETV7	BC106753.2	OR5H14	CYCS
ANG	MAST1	GPATCH2L	IFNG	MTMR2	CFAP70	DSTN	SAP30BP
ENTPD6	NXF3	CD151	NDUFA5	MAP3K7CL	MAGEB1	MEA1	NDUFAF2
C10orf62	WRAP53	OR8H2	ERF	RFPL3	STXBP3	ARNT2	TEAD2
NAP1L1	ARID3B	UBL4B	ELOVL2	PLA2G6	PF4	GSTO2	TMEM37
TMEM139	VGLL4	ANKS3	CEP57	NAPRT1	ATG5	SSFA2	GNB5
NMB	NUDC	ZC3H7A	RAB30	SIRPD	FMO2	XRCC6	OR2G2
WDR27	ZNF398	TCEB3B	GUSB	RECK	SYNE4	KJ903238	EGFL8
SOX6	OR2M4	OR51F2	EP400	JHU03939	BC014212	CCDC82	CAPZA2
SLC17A3	RNF6	HMGCL	ROM1	TRMT1	ADAMTSL4	C12orf66	ZNF92
KCNA2	GALNT1	OR5H6	IFRD1	PHTF2	ABHD1	MYO1B	DPP3
NR6A1	DAB2	C9orf61	TNP1	CDKL3	HSPA1L	SGOL1	LYVE1

TMA16	FGD3	PARP6	MLIP	IFT57	P2RY14	PPM1K	ATF7
SPATA3	ANKRD46	CSTF3	DAZ4	SAP30L	MTPAP	RPS29	PTPN5
PAK2	ZBTB33	RPL23	SUGT1P3	CRIP1	GK5	RALGPS1	CASQ2
RFESD	PACSIN2	UBE2E3	PSEN2	ARMCX2	TAAR1	SNRNP40	RPL3L
BC048328	UBP1	CNOT8	MAGEC2	OR10AG1	OR2F1	KCNN3	NPHP4
UBE2O	TRAM2	MSANTD4	DUSP23	SERF1A	RBAKDN	TNFRSF11B	LRRC59
SRSF10	SPATA4	CCT7	OR6N2	SERPINI2	COG3	HMGCS2	ARHGAP24
OGFOD3	EPSTI1	KIF16B	OR7D2	M0R1X1	STK16	PRDM14	HSPH1
SEPT2	STARD3	CSNK1G1	LBR	CDH13	KATNBL1	TMX2	GCA
XAGE1A	SP140L	MAPK1IP1L	C22orf46	GAGE7	ABHD10	MAGEB4	KJ900872
OOSP2	SDC2	SLC1A3	OR2T29	UTP14A	TTC23L	WASL	FAM47B
ZNF880	SMARCB1	CYP2E1	IGSF8	ABCC10	BIRC3	SNCA	KJ906206
LOC643406	EEF1D	WDSUB1	CYTH2	GAL3ST4	NG_042316.1	NF2	PCSK2
LPAR6	GJA5	ATAT1	NEK10	YES1	LRP1	MTG1	PRPS2
JUP	MFAP3L	KJ903518	ERI1	P2RX1	JHU04032	DAPK2	RWDD2A
POLR3F	HDGFRP3	FREM1	CXCL1	UCHL3	XRCC5	PSG9	IMPA2
GK	PARVG	FRMD3	S100A9	PTCH1	C6orf62	C18orf32	THOC6
GDA	SZT2	DYNLRB2	CTBP1	SLC22A12	CCL13	SCEL	KLHL29
ACTL7B	TXN	POLR1D	FAM175B	WWP2	GOSR1	C9orf62	C10orf107
NMT1	OR7G2	TSGA13	MPP7	SRPK1	JDP2	C1QTNF2	PRKAB2
GFRA3	RABGAP1	RAB27A	BCKDHB	LRRC23	CLASP2	FZD4	FEZ1
AEN	ZNF461	ACADL	ZNF358	GZMK	OR2H1	QPCT	OR10Q1
OR8D1	KAT5	MAP4K5	DCUN1D4	SERPINB1	INTS5	MTMR12	PLEKHG5
TMEM99	TMEM204	SMIM19	ATP4B	TMEM168	RNF38	TMEM176B	ACTR3B
TP53	SYT11	PPP1CB	FCRL1	TMPRSS4	FYTTD1	XKR3	OR10K1
OR8D2	SRSF7	KJ900931	OR2A4	EIF2D	PTK2B	NCSTN	MRPL22
BRE	RTN4R	KCNH6	RHBDD3	C2orf49	PRKAB1	RPS12	SPSB2
NFYC	MRPL17	MAX	NKAIN2	ATP6V1H	OR14C36	OR10D3	TPSAB1
FAM200A	ABCG2	CREB3	MRAS	MC4R	RELL1	KJ905766	TOMM5
STX4	CCND3	MYL6B	HILPDA	CHCHD1	EGLN3	HHATL	SH3BGRL3
CALHM1	TRAPPC6B	VBP1	ALDH5A1	TDP1	OR8A1	OR4A15	BCL6B
ZNF613	KJ903863	CD244	MMP12	ANKRD13A	SLC9A6	TMEM31	KJ900990
BAALC-AS2	PTGER3	CELF1	PIP5K1A	FAM3A	IL6	RAX2	BAIAP2
LTF	SDSL	RNF13	SMAD3	TMEM200A	JRK	CBX8	CDH7
OR4S2	CAMKMT	BRD9	RALGAPB	FBF1	PCCB	SKAP1	PLB1
CYP3A5	FGFR1	PDP2	BEND5	RPL30	ATG4D	C4orf27	GNPNAT1
GREM2	ACD	OR8I2	SLC22A17	IQCK	GFPT1	CSH2	TUBA3C
NSUN3	ANKRD30BL	CDA	NUDT12	CCL8	HOXC8	PLEKHA5	MOB1B
KCTD7	TEX12	PRMT7	BDKRB1	STBD1	BLZF1	GATA3	B4GALNT1
GATA1	RCOR3	HSFY1	PDE12	FERMT1	GPM6A	CYSLTR1	AFAP1L1
TAAR8	OSBPL1A	METTL5	AEN	TSKU	FAM13B	TDRD3	USPL1
NAA50	CLDND2	HTN1	BC029877	NCR1	C2orf57	TST	KCNJ3
C4orf42	CBX1	TXNDC11	SLC12A1	TMPRSS2	TCTN1	RAD21	OR4F3
PHF20L1	STEAP3	RCOR2	CSF3R	MAT1A	DDX20	FUT9	ZBTB6

C20orf43	CRISPLD1	B3GNT2	MYD88	CDK5	SIKE1	MYRF	CHTOP
MS4A4A	BCL11A	SPAG8	CDC26	OGN	CA14	GABPB1	PFDN6
LSM12	P4HA1	NUP98	OR2A12	SLTM	NR1D1	KJ900968	CNR2
CTSC	ZNF8	UBE2H	GPC5	RHOBTB1	TFF2	TFAP4	KLF4
CCDC106	ADH5	EFNB1	PDK4	TFAP2B	SLC38A2	ECHDC2	TMEM255A
PARPBP	OR2W3	CYSRT1	NMI	C9orf85	NCOA4	PARD6B	STAT1
CTNNB1	GALNT6	DHX29	TSPAN7	RCSD1	CARTPT	PITPNC1	HAGH
JTB	AKR1A1	JCHAIN	RPS9	TMEM47	FNDC4	MEGF10	OR52B2
PROC	CYTH4	LRRN3	TNFRSF1B	SMAD4	NUDT13	TAAR2	DNAJC16
DNAJC2	FAM219A	KLF3	PPIL6	GRM3	BUB1	FLRT1	RNPS1
TACO1	RAB4A	PCNP	BRMS1	GJB5	TREM1	SYT9	P2RX4
C6orf1	ZNF18	GABRE	CECR1	DARS2	SULT1C4	CATIP	SNPH
MR1	CDHR1	FAM171A2	THEMIS2	NT5C3A	FGFR1OP	MPPED1	ALDH3A1
PVRL4	EIF3K	PPP1R35	EHD2	SLC25A40	RPL3	PLA1A	SLC36A4
STXBP4	PAQR5	CCNE1	KIF16B	OR1F1	SART3	RPS6KA1	KRT6A
CCIN	KIF23	MBOAT1	PLA2G7	DEPDC7	MGC40069	BSG	RDH13
TMEM243	PIP	KJ902863	CNIH3	CFHR3	RAP2A	B2R550	GGT1
MC2R	PLCD1	ADIPOR2	ENTHD1	ADAM2	XYLB	PSMB5	ADAMTS12
TCEAL8	RASL12	GLRX5	OLFML2B	PTPRS	TAF6	GLB1	MDH2
G3BP2	NMNAT1	HPS3	TOMM70A	PRMT6	ZNF385A	FBXO39	CCDC91
AP5Z1	NAT1	SEC31B	OR11L1	RPL28	ADPRM	UBL4A	PDGFRL
NFKB1	PNPT1	ZDHHC13	SMC3	ERMN	PRRC1	RPP21	TIMM10
LSM8	NEUROD1	MITF	PHYH	GPBP1L1	MED17	SOHLH2	SH2D2A
LSR	OR6P1	STRAP	ARFGAP1	ANKS1A	PKD2L2	MPHOSPH8	AMBP
POLR1E	SLCO2B1	OR5I1	PAK1	FERMT3	GTF2H2	SNX13	MBNL1
ZNF19	CHEK1	KLHL11	MTMR1	FGL1	RHOC	PDLIM7	DYNLRB1
MAPK13	HDGF	NUMB	NFYA	MKX	MTFP1	MED20	HID1
CD53	RETSAT	SEC14L2	CLIP3	TRAPPC8	VCY	KCNAB3	ZNF449
LIMCH1	NDOR1	BC039235	PCDHB16	CD46	CCDC53	UCHL1	HSPB11
FCGR3A	H2AFY2	TMEM109	GAGE1	ST3GAL6	USP2	DNMT3A	DGCR6
PGM1	PON2	PMM1	DCAF4	ATL2	DYNC1I1	C10orf82	GGA1
KJ904388	ACTRT2	ZDHHC11	MFSD9	GNL3	SMIM12	FKBP10	MAS1L
MRPL3	RBM41	XRN1	DNALI1	EIF2B2	OR5A1	BID	EPHA2
ACOX1	CCDC110	PCBP1	IKBIP	NR0B2	SPATA22	APLP2	CLN8
IL13RA2	GMPPA	RIMBP2	LUC7L	ERAP2	FAM90A1	ZNF480	FAM227B
PTPRG	OR9A4	KDELR3	COX4I2	ZCCHC12	TECPR1	SLC26A2	TMIGD2
ZNF596	SNAP47	TMC4	DNAL1	GIMAP4	CST7	SLC16A10	TMX3
TSEN2	RSPRY1	OR10S1	RASSF5	TTLL2	GSPT2	ARHGAP20	OTUB1
SLC47A1	MORN3	BCAR3	CDK3	C1orf105	VSTM4	PARG	GLRX2
PCCA	PDS5A	CARD14	NQO1	MAB21L2	FADS1	GPN2	PRSS8
GAK	SPOCK1	GLRB	MTIF3	DFFB	TMEM173	NPY4R	HSPE1
MMADHC	ZBTB8OS	GSTM5	PELI3	HABP2	RAPGEF1	CTDSP1	SUMO1
IKZF2	TRAF6	PNO1	KIAA0101	OR51E1	BTBD10	LINC00851	RNF219
OR10A3	GPR18	MDH1B	KIF2A	PLVAP	PIK3R1	APOBEC3B	NDUFA13

IYD	STPG2	ZNF718	PSMA5	UNG	CCBE1	CD80	SPECC1
NFIA	ALKBH7	EGLN2	FOXS1	SLC50A1	YK038	G3BP1	PPP2R1A
ZDHHC21	CREB3L4	SHC4	TLE2	TRAPPC10	EDN3	LMNTD1	EMG1
LRRC3B	FSCB	ARFRP1	HS3ST1	KJ902641	FAM49B	DHX36	PREP
C8orf59	P2RY12	RET	SSR1	TIMD4	BRF2	SPARCL1	TOB1
TMEM206	LNX1	SLC35F3	ALG1	CRKL	LCN12	TBL1XR1	OR2A2
ZDHHC4	SLC16A7	ZNF322	CTR9	INPP5B	RSBN1	POLR3E	TNFRSF10B
ATMIN	CHKA	CHDH	ART5	FCGR2A	CLEC3B	METTL13	RNF141
GTSE1	TMEM43	TRA2B	NUP62	EXTL2	FAM131B	KJ903373	SPCS3
CWC15	RPS24	BBS5	GLRA2	FRMD5	FAM92A1P2	ELK1	EIF2AK2
PSTPIP1	ESM1	GRAMD1C	CRABP1	LINC00305	IL12RB1	FAM151A	MGST1
C3AR1	TMEM234	PRL	MGAT2	RLIM	OR2T27	CD68	FAM71D
NR2C1	KPNA5	TREX1	CDK5RAP1	CAPS2	RAB3IP	FAM43A	ANKHD1
MCM5	FAM71F1	KIAA0712	IQCF1	CFLP1	ARMCX3	ZNF655	EDC3
SNRPG	RIT2	SLC25A41	STIP1	OR6N1	LRRC39	TMEM246	TSPYL1
RGS22	CEP104	FTSJ1	ANGPTL4	DUSP16	OR8B3	TCL1B	SARS2
SPG11	PDE4DIP	SECISBP2	LPGAT1	MFAP1	TBK1	NBPF15	PDE4A
PDCL	ZFAND5	REXO2	VAMP2	VAPB	PKD2L1	CIDEA	ZNF544
SCML4	RRAGB	CCDC89	FUNDC1	COMMD7	DDX28	BC047471.1	ZCWPW1
GPR1	CLK2	ZNF433	SPRR1B	HRH1	BEST1	GPLD1	FGFR2
KJ903117	SLC25A48	NPY	AGPAT2	TDP2	CKMT2	NCBP2	RPL39
ZKSCAN1	TBC1D2	WDR62	OR4K14	NDUFA10	SNX32	ERO1B	KIAA0226L
FBXO22	TRMT10B	SPOCK2	DGKA	SMNDC1	FAM27B	ENTPD4	EIF2S1
KRT81	NDC1	BC016981	KJ902878	GMPS	NEIL1	CELF2	GPR3
KCNJ13	ZSCAN1	PILRB	FANCA	ZNF620	TVP23C	GPATCH4	LOC105372277
CA10	MAPK10	BHLHB9	PPM1A	MARCH10	ZBTB9	SUN5	IL1B
IFI6	HLA-DMA	MAGED4B	AGR2	BC033172.1	SLC39A14	GPR82	GOLGA6L9
GPNMB	PPEF1	BOLA3	ARNT	PGAM1	PRR16	MYL5	SEPT12
RAB5C	IL1RL1	ABCA8	LINC00311	PGA5	DMTN	URM1	SLC35G2
ACTL6A	SLC22A24	MAP2K3	HMCES	GPSM1	SRFBP1	CAMK2A	CACNG3
WARS2	OR52D1	OR1L3	DDR1	C5orf24	SSX1	CHURC1	STK31
TF	MAEA	CXorf50	CEACAM5	KJ905804	ATAD2	THRA	PSENEN
ESAM	SLC30A4	DHRS3	LRG1	SMYD3	DBNL	TTBK2	FAM78A
CLEC10A	AMZ2	OR2J1	OR5C1	TMBIM6	MAPK11	ZNF740	TAF9
CHN1	CRTC2	ATP1A2	BPGM	CERK	CPQ	KLF7	CTRC
MRPS14	IL20RB	C7orf61	UQCRC1	NPY6R	OR13C3	AQP2	RNF168
KCND1	C12orf50	THAP5	PCBD2	Q5T6C4	MC5R	CPED1	TMEM184C
NEUROD6	YPEL4	ATRIP	IMP4	TPD52L2	KJ904364	PSME2	HCFC2
NUP54	C5orf58	HLF	TBC1D21	OPRL1	C17orf64	LILRA6	ELF2
PRKCZ	OR3A3	PROSER2	CHRM4	PODXL2	GTF2H5	GPR15	ZNF384
IDH3B	SLC40A1	SESTD1	PLA2G2A	ENHO	DPY19L3	STEAP4	USP18
RHCG	ST8SIA4	UGP2	GNG10	DEPP	ZNF692	ECH1	ACTG2
ERG	ACPP	ACSL6	OR1A1	OR5T3	NKD1	SMARCAD1	DNMBP
SLC17A5	ADGRE1	RCBTB1	OMD	PRCP	NGFRAP1	TMEM234	DLGAP5

MTHFD2	C3orf18	SLC30A6	SPATA2	OR5P3	OR2A1	RBM11	SYT3
KHNYN	SOCS4	SAMD4A	GALNT3	MORF4L2	CCL4L1	MOK	KIAA1217
PIH1D3	SH3GL3	GPR61	GAL	PFKFB4	SLC46A1	CH25H	SDCCAG3
INPP1	EEF1G	DNAJC28	SPON2	CTSD	SIGLEC6	C1QTNF9	GPATCH2
CCR8	FAM167A	KCMF1	LYSMD3	VIT	OR9I1	HTATIP2	IARS2
LZTS2	XPO1	CHODL	CHIC2	KJ904407	NMES1	TRAPPC6A	RPL38
TFEC	CSGALNACT2	DNM1L	PDLIM1	RTCA	CSNK1G3	BPI	SLC5A10
THG1L	KJ903742	CREG2	SDR16C5	NR2E3	GATA2	PCDH8	SOCS6
IQCA1	CCR1	LIMS2	ZNF41	ORAI3	STOML3	PSMB3	SLC22A9
MOG	HIBADH	ZNF385B	NDUFS6	ZNF548	CDC25A	SRSF11	ACTR2
FBXO21	RBM6	BC057848.1	CXCR2	TMPO	SENP3	TTLL6	SMDT1
SEPT11	DNAJC18	GABPB2	TTPAL	TPST1	RAD23B	PROCA1	TMOD4
ACADM	TDG	XK	ABHD17B	TMPRSS12	BLCAP	OR9Q2	RPS17
MFF	NDUFAB1	SIRT3	HTR1F	MAP2K7	PLG	MAG	AMBRA1
VWA5B2	ZNF136	HG505487.1	KLK7	SEPT8	SUPT4H1	ANXA10	PFDN2
NTPCR	CMTM8	CFAP69	OR6C4	FAM58A	SUCLG2	PDS5A	TSGA10
FAM204A	BAG4	REEP6	CPNE5	IL17A	ANKRD18DP	ZHX1	CLK1
RNGTT	PPT2	ALDOB	RPL10	HIST1H2BC	DECR2	RNF10	BCL2L1
VAMP1	SLAIN1	LYPD3	B3GNT5	EMP1	CCDC190	LRRC56	OR9G1
CARS	CCR9	TAF15	SOX5	SPANXC	SLC31A2	LILRB2	FAM160B1
C2orf40	EXOC3L1	KJ904289	ELOVL6	C16orf89	HEY1	ARF4	MGC9913
ZBED2	NAT14	IL21R	OR6K3	PLAUR	KLF12	BMPR1B	NIT1
GALNT7	OR9K2	P2RY13	ATP5A1	ANGPTL2	GTF2B	NLRP2	CABLES1
NTNG1	PLD4	S100A7	TBC1D19	SNF8	GABARAPL1	EIF3I	RPL23A
MRPL11	PRND	KJ901665	CES1	OR5A2	GPBAR1	CAMK1D	METTL25
CAPN11	ANGPTL3	PXN	TMED4	CEP41	AQP8	OVCA2	NXPH4
C10orf92	C16orf13	LIMK2	PRPF6	FAM46A	USP12	ARRB2	IDH2
CCDC68	BEND7	ZMYND10	TUBB	GDF5	STPG1	TOB2	DEGS1
SIRT1	SLC6A18	PIGX	NAA38	GNG7	CDK2AP1	NENF	STAT5B
RDH12	TRIP6	C9orf116	CD300C	PTGS1	OR4F15	ZNF3	TMEM135
PPHLN1	TCEA3	KIF3A	BC041787.1	PLK4	SLCO2A1	ASIC4	GABRA3
COPB1	HDAC8	BMP5	PI4KA	SWT1	PARM1	PTGDS	CHMP3
EFHD2	INSL4	RBM4B	TSPAN8	C4orf19	KLHDC8A	ANKRD36	CD3E
BRD3	KCNIP1	REG1A	FAM217B	RABEP2	PAGE2	RPUSD2	DNM2
PCK1	DUSP22	CTD-2643K12.2	SLC25A36	RNF185	POP7	PNPLA4	MAPK14
APOL1	GGH	CCR5	GOLGA4	ABCC5	LINC01547	FGD5	KJ900999
GPRC5B	TMEM117	WFS1	SLC16A4	JAKMIP1	PSD2	FGFR1OP2	ACBD7
TXLNGY	MNDA	ALLC	FUCA2	MRPL34	FKBP3	KIAA0513	SH2D3A
CHRM2	ANGPTL7	RBMS2	ENPP7	GJA9	PCGF5	CARKD	ZC3HAV1
OR1M1	EEF2K	ASMTL	CYTH3	ZC3H3	ST3GAL3	CFH	ZFP64
PRPF18	CFI	TEKT1	BC033035.2	IGFALS	PRR22	FMNL1	STK33
PLEKHA3	PNMA1	AHSP	OR52N5	UBE2L3	FBXW5	LEP	USP16
BRPF1	RFWD3	ACLY	GFPT2	MTERF3	DUX3	MFSD8	CEP112
OTUD6B	RPS4Y1	EIF4EBP2	GYPA	CCDC12	NCALD	BEX1	CHMP4A

SRBD1	C6orf165	BVES	FAM3B	REEP4	PCSK4	WDR63	TBRG1
TPD52L1	KCNK17	TIMM44	GALNT12	GPANK1	POU2F2	GIMAP5	LECT1
CSNK1D	KCNAB1	EVA1C	STX12	MTFR1	KJ900829	LINC00638	PGPEP1
H1FOO	OR1J1	CDK13	PCDHA7	KCNA4	ABI3BP	LDAH	IFIT1
CTTN	IMPDH1	TAF9B	CXCL13	ALKBH3	CCDC97	TSLP	Tex35
HARS	OR6B3	OR2F2	CLEC1A	CTPS2	ALOX15B	NRSN1	SIGLEC9
SNRNP35	PLA2G5	VDAC3	NIM1K	CCL3	PPP1R42	CXCR4	PTRH1
USP36	ANKRD53	NMT2	CBFA2T2	TRIM65	C3orf52	MCCC2	UFC1
C15orf41	CDKN2AIP	OR4D5	CNST	KLHL8	DTL	CAPNS1	RNF112
PRRG1	AC246818.2	LRRC57	LURAP1L	DNAJC5G	IL7	WDR47	FBXL16
CPNE4	OSGIN1	CCNI	PEX5	ASPHD2	ATXN3	AL080197.1	LRRC34
KJ901880	CTBP2	EHF	PRKACA	ABCC6	OR2AG1	AGR3	TMEM237
IRS1	PHEX	RNF26	TRAIP	ILVBL	RGS20	TTC12	TMEM143
FBXO2	OGFR	HHIP	ACTN1	ADAT3	NOC4L	FBXO36	SH2D3C
CSNK2A2	IGLV6-57	HAPLN3	BC025318	PLEKHF2	CD209	CSGALNACT1	YU004
WSCD2	TCEA1	SLC25A25	MANEA	MED22	FAM19A3	PFDN5	MTMR1
MGC13053	SOX8	FAM71C	PFKFB2	AHSG	NCBP1	METTL15	GCDH
RNF125	NOL12	TCTA	FBXL20	AMN1	TMEM154	DEGS2	ZGRF1
ASTE1	CAPN3	LINC00996	LINC00597	NDRG4	MGAT4B	IGLC2	XRCC6BP1
PLIN1	UNC119	TAS2R38	FSIP1	HAS3	SLC35C2	GYS1	TFAP2C
SERF2	PRAMEF1	MAGEF1	UCK2	FAM53A	IL1R1	NOL7	KCNJ1
OR51I2	ULBP1	AMZ2P1	CMTM1	CD8B	TSPY26P	PPP4R4	ARSF
ACTC1	RNF138	KJ900886	IGFBP3	CD99	NT5E	PROS1	USE1
SLC35C1	PTCHD1	CDKAL1	PPP2R3B	SOGA1	GMFB	P2RY10	GGA2
OGG1	FBXW5	EREG	ATP5G3	TET2	SLC48A1	FSCN3	XM_014344644.1
NHLRC2	CACNG4	ZKSCAN8	BACE1	AIFM3	SSR4	CDK20	UXS1
FAM168B	PGBD2	PCDHGA12	TRIM35	EGR2	BCORP1	MPZL1	GUCA2A
GCNT4	SLN	EPHB1	EFNA3	BCAN	UTP18	TPRG1L	MCF2L
CDCA4	PCMTD1	ZNF839	PXDC1	ERICH5	BPIFA3	ATG10	ANGPT1
SLC7A2	MBNL3	SLC25A2	TSNAXIP1	CNN2	C3orf70	LDLRAD4	IL12RB2
MS4A6E	MYO3A	TEX29	AK8	REG3A	OXSM	PNPLA8	C19orf43
NSMCE4A	CXorf21	S100A14	YIPF5	LENG1	ZNF483	C7orf13	EXOC3
PSPH	EPG5	SEP15	SPPL2B	PDDC1	ITGA6	RORA	TAC1
PDE7B	APOL3	GRAMD1A	GCC1	ADAP2	RASD1	CISD2	FCER1G
TTC16	NCKIPSD	EEF2	BC030956.1	TMEM11	BZW2	TRIM46	CSDC2
VEGFA	C1orf228	RNASE7	SLC4A1AP	MT1M	IFNK	OTX2	CCT2
CORT	PDE7B	DUSP21	RIMS4	DIABLO	SLC39A13	CCZ1B	SEMA3B
PNKP	CST3	LPIN1	DDRGK1	FLVCR2	SPTLC2	PLEKHA1	ETFDH
EIF5	SPSB3	PAFAH1B1	SCLT1	OBP2A	A2ML1	DSC2	TARDBP
LYG2	IKZF3	VPS16	APOOL	IKZF4	ZIC3	MED28P3	OR2S2
FBXL16	FCGR2B	ABCF1	CNPY3	NR2F1	CPM	UBXN6	FAM83A
CXCL2	FAM221A	SMIM8	ORC6	KHDRBS3	CCNC	JAG1	PAX5
ZNF192P1	SLC4A8	BTBD8	BTN2A2	RNASE2	KLHL20	GH1	JPH2
HLA-C	ZNF511	PTPN1	CCND2	CUL4A	MAPK7	CABP4	ACTR1A

RGP1	NDEL1	PTPRA	LAMTOR2	FAF2	PEX13	SRRM3	PNMA5
C1QL1	SCTR	CSK	ARHGAP17	BCL2L11	CDC42EP1	MAN1C1	LIMD1
KATNAL2	TAS2R9	MMP19	CCNJL	ATP5J2	FAM27E3	MICU2	VN1R5
SYCE1	TERF1	EXT2	SSTR1	DRAM1	CD55	GUCD1	ERVFRD-1
HMGCLL1	CDK18	ZNF558	TAPT1	OTUD4	PATL1	LOC105376261	HG508603.1
IL25	GJA10	BLOC1S2	AHNAK2	TRAF3IP2	GIF	MSH4	SPHK2
PGRMC2	GRB10	MAD2L1BP	SLC25A39	SLC2A4	ELN	PSKH1	HEATR1
YY1AP1	ZNF718	PIFO	SMIM24	CRAT	ZNHIT2	REG3G	TNFSF11
WHSC1L1	PCED1A	SLC25A16	LRWD1	TRAP1	F2RL1	WDTC1	SLC12A7
KLHL12	CTDSPL2	RNF182	KCNS2	DLX6	COQ9	WDR92	CD200R1
OR52A1	KRTAP13-3	BBOF1	CXorf51B	KIAA0430	PRAME	LOC440700	URB1-AS1
GALNT4	BOD1L2	LINC01126	MAP3K19	DHX58	CPE	DIS3L	RTKN2
CARF	TMEM70	DPY19L2P1	HESX1	CELA2B	TMEM97	PICALM	BMF
SLC5A11	WHSC1	GRIA3	C9orf152	PIN4	LOC102724398	AK123718.1	CDK10
BTD	DEPTOR	DMWD	NDUFAF1	SMO	APEH	AATF	NOL3
CCDC155	COPB2	TSPAN15	UBQLN1	NOL4L	KREMEN1	TBPL1	PARP15
ZNF808	DSN1	RNF19A	ZIC4	RUFY4	TM9SF2	DALRD3	TAS2R8
ZNF830	APOA2	CLEC1B	FHIT	STK36	TM7SF2	WDR1	SND1
MTF2	ICAM2	CIRBP-AS1	KLHL23	PPM1M	CER1	PRH1	PVRL3
AFG3L2	TMPRSS3	ITGAV	IFNA5	KLK9	LARP7	EXOG	CCNB3
ITGB6	HG504694.2	FUT1	CDK6	FGF1	EMP3	MGRN1	ATP12A
MARCKSL1	BACE2	LPAR5	KMO	FBXO7	POFUT1	FCRL2	RGS8
RPS6KA5	ASB5	DEFB103A	CDC14B	CSAG1	C8orf37	ZNF791	LRP5L
C17orf89	CCDC15	TCF7	TBX5	NUDT1	TMPRSS5	TMEM45A	FOXRED2
FCN1	IRF3	BC032125	HEPH	WDFY4	ATP6AP1	COPE	NIP7
GBA2	RAB2A	NG_004831.4	MAGEB6	POLR2J3	SLC7A1	UGT2A3	ITGB1BP2
TRAF3IP1	CXCL12	CERCAM	PPP2R1B	FAM19A2	TBC1D13	WWC3	COX8A
ATE1	ITPRIP	STK10	ANKRD39	KCTD17	LRRC42	TSPO	PDSS2
UBTD2	RGS5	AGBL5	TCERG1L	KLHL17	ACAT1	NDUFAF7	ABHD11
IL21	MYL10	KJ903469	ATP6V1B1	TRNP1	SNX19	ACRC	LINC00092
DCAF17	BAG2	CRBN	CCNG2	IFI44L	INO80E	SLCO1C1	SOX9
LILRB1	KREMEN2	TFPI2	RAB7B	UBQLN2	SLC10A1	SEC23IP	CHORDC1
PABPC5	KRTAP19-5	CDS1	MED26	ATG16L1	C1orf112	LY6K	CCS
ARGLU1	ARMCX4	PPP6R2	FAXC	MRPL1	CXCL11	ZBBX	CEP89
PPFIBP2	ST6GAL2	LRRN4	OTX1	MRPL50	PRR35	PTTG2	TIGD1
RD3	HIBCH	PLD1	CD1B	HIP1	CLEC5A	THEM5	GABARAP
PADI3	SLC51B	PPP1R13L	C11orf63	CLDN18	LLPH	SNU13	HLA-B
NPTX2	SLC2A6	YME1L1	MMP23A	ADRB3	C9orf43	PRPF4	PRADC1
PCDHA8	PNMAL1	FAAH2	KRIT1	XR_608919.1	VHL	ADPRHL1	VN1R1
PRKAG3	GPR176	ZNF683	DYRK1B	C19orf52	ACSBG1	LONP1	WNT7B
LGALS3BP	OR5AN1	ZSCAN20	HSF1	TGFB1	BC067080	CST4	CFAP161
CSRNP3	CCDC40	CFAP58	LPAL2	AK055254.1	RBBP7	WNT3	FUT3
CACNB1	IL16	ASPSCR1	RRP36	KYNU	PCDHA2	VPS13B	HNRNPAB
OR52W1	RNF126	GAS7	ANKRD16	SCGB1D1	ZNF239	SLC52A1	TYRP1

OR4C13	FBXO11	FBXW7	SHISA4	KCNK9	GNGT2	SCN2B	YP021
CSTF2	CMTM4	MMP9	GSDMB	C9orf45	ABCE1	WDR83	CCT8
EXD1	TARS	ZNHIT1	SERAC1	UBR3	SLC18A2	CXCL17	HHAT
HS3ST5	TYW5	ZC3HC1	C11orf52	GCSAM	ARRDC1	KJ903107	SMARCA5
POLR3D	SLC25A13	SPESP1	TGFA	ELOF1	DAB1	TP53TG3	ZC2HC1A
CXXC4	ODF4	RP11-47I22.3	LOC100131303	ZNF326	THAP8	CHTOP	UBXN7
GPATCH3	TULP3	RGS10	PLCL2	ADRA1A	ZNF414	CLDN8	NOG
CCDC115	RAD23A	TEAD4	HMBOX1	MT1B	DPY19L2	FZD7	NEK8
TINAG	WFDC9	BTBD3	ITPKC	LDHD	DCBLD2	RP11-421P23.1	RP11-129K20.2
AHSA2	XAF1	CCDC141	ZNF821	FAM57A	NR2E1	PHTF2	PACS1
MFSD3	USO1	TCOF1	HIF1AN	QRSL1	EPO	MEOX1	SLC9B2
NUP43	RMDN3	RPA4	TEX13B	DDX10	AMELX	IL22RA2	NDUFS8
AC018359.1	C2orf73	ADNP	MDM1	FOXJ2	ZSCAN21	ANKRD49	STARD8
GDI2	SLC35F2	ADAMTS1	RNF135	HOXD4	CXorf38	USB1	PPT1
EPB41L1	IL27	GDF2	POMT1	DUS1L	PDK2	NCBP2-AS2	ALOX12B
FTCDNL1	LINC01511	ITPA	SNRPB	PGLYRP1	NAB2	FAM189A2	CSE1L
KJ901215	MUC20	PRKCB	GABRB2	KRT19	C2orf47	OGFRL1	TSPAN18
IER2	LCK	RTP2	LCE3D	NKRF	PCBP3	MAPK15	CRB3
POGK	MPHOSPH9	C4orf26	DEFA4	PTPRCAP	MAATS1	KJ902886	DNPH1
CCT8	CLDN19	GPRASP2	NCDN	FAH	SPRTN	VAMP8	SRD5A1
RTN1	CLCNKA	KLK13	GPR45	HG507164.1	PTGER2	OCA2	IFNW1
ALS2CL	HG492274.1	CHM	FGF16	CCDC69	TCEAL5	IL23A	ANPEP
NRF1	ACADS	FAM46B	HGS	EEF2KMT	TES	THBD	GOLGA5
STXBP2	RAB43	LMX1A	HTR4	CHML	BCL7C	SYCP2L	DPY19L4
DPYSL4	DUS4L	TBC1D22B	SLC25A21	E2F7	OTULIN	CCL24	TC2N
GNG11	PCDHGC4	NOP58	RARRES1	RASL10A	TNFSF18	CDKN1B	MCCC1
SLC7A10	SCARA5	BC047522.1	RRAGC	BRICD5	MRPL28	RGCC	PCDHGC5
GGT6	PPP1R16B	PLEKHG6	PSD3	SPRR4	MAVS	LINC00518	NSUN7
DCAF8	GPR141	AZI2	QKI	TASP1	NTSR2	UBOX5	CHRNA6
REEP2	TIMM23	PNN	COPS5	DEFB119	PGM2L1	MASP2	FANCF
LINC00928	IFNE	NPFFR2	TMEM260	STK26	TRIM29	TMCO4	GAMT
PLK3	CDC42EP2	CCNF	E2F4	RCN2	MPDU1	PVR	DDX42
KIAA0141	XRCC1	ADGRG3	ADPRH	KRTAP26-1	MMP27	CBX7	PFKFB1
ADAR	RAB11B	SMYD2	HG502449.2	GLTSCR1L	AK312033.1	ATOH1	BC008618.1
LSM1	RASSF4	BRI3BP	LKAAEAR1	LINC01587	SECTM1	MOV10	KLHL25
ZNF177	GJB1	BOD1	RSRC2	UNC13D	TMTC3	SCOC	MYO9A
MKKS	RFPL1	SNX21	WFDC6	PDE7A	CAPN7	BOD1L1	MATR3
TRIM4	MTRF1L	HNRNPU	BAP1	PGM2	DNMT3L	FSD1L	RFX2
FST	OR10P1	C1orf131	GK3P	DMBX1	KRT76	PPP1R2P9	C22orf31
SEMA6A	TNFSF8	PHACTR1	TMEM107	GAD2	RSRP1	GIPC1	CCL27
OR2W1	ATPAF2	ADARB2	RSPH10B2	TMEM160	SLX1A	SF3B4	TUBA4A
STATH	SCNN1A	WDR73	SCHIP1	RIOK1	IGF1R	POMK	COLCA2
BC033201.1	CREB5	RHOJ	NOVA1	C17orf77	ZDHHC5	NKX2-5	SDCCAG8
TRIM17	MAFF	TMEM63A	CNKSR1	POLI	HENMT1	KLK10	NME7

OR5F1	PTCD3	CAPSL	CACNG2	DCP2	RAB40C	BRINP3	MRPL18
TPPP	MGAT5B	C11orf72	SETD6	DDAH1	BRINP2	CREBRF	MRPS10
ZNF205	TMEM219	NUP37	EEPD1	ZNRF4	AC025171.8	CPSF2	VRK2
CLEC4A	MUCL1	CTXN1	LINC01104	XRCC6P5	EPB41	TIRAP	KJ902417
DDHD1	PCGF1	SARG	GSTA3	IL17RC	FXYD6	CHCHD2	LCN1
ABCA6	KCTD18	VPS53	EOGT	GRM7	B3GLCT	Q5JUA8	ZNF396
CSF1	RBPJ	AKAP7	C22orf39	MMP8	IFNA17	FFAR2	MTA3
WDR22	EIF2B5	CPOX	EFCAB14	NADSYN1	H1FX	ATP1B1	SMCO4
HDHD3	P4HTM	AURKAIP1	LGALS7	PHF11	GNLY	TPH1	PCDHGB2
PRLR	ST6GALNAC3	MPP5	MOB3C	DTNA	BARHL1	R3HDM2	LIG1
MED11	TAS2R45	ATP5S	MT2A	BC030111.1	PUSL1	SYNCRIP	HOXA6
FAM109A	ATP1A3	KJ901018	SLC22A6	ARPP19	RAD51D	ZNF428	PAQR3
PDSS1	SPRY1	LYZL6	ICK	JHU10828	LINC00921	BEAN1	RNASE8
CYP4Z1	ACOT8	TCP11	PRRG4	RALGDS	RNF5	ILKAP	PCDHA6
TRIM8	SDF2L1	SLC35B4	RPS26	C8orf45	GABRG2	PNMA2	MTHFD1
VENTX	ALG2	SYCN	C2orf74	ARMC6	SPRED1	FAXDC2	UPRT
LAPTM4B	OR7A17	MLKL	LMAN1	NLGN4Y	XBP1	HMGCR	SETDB1
RPL18A	DKK3	PPM1B	SLC25A17	IL22	LRRC4C	LIPH	GAST
IL36G	COX5B	CLDN17	IGSF1	C1orf146	CARD18	AC137794.2	AK093936.1
CCDC88C	CTNNBIP1	STK4	ZNF550	TMEM151A	SPATA2L	ZSCAN4	RBM42
KLC2	PPP5C	NIPAL1	ZNF510	KRT79	EP400NL	EMB	ZNF471
TIAL1	LOC105369727	SYT6	IL36B	OLFM4	OR7A5	C1QB	ORC1
ENG	ANXA1	COL14A1	HK1	GTF2H1	POLR2M	RALYL	TMEM179B
IMPDH2	TRMT2A	HCFC1R1	OR10G9	PCDHGB1	ZNF530	AP1S1	PDGFC
CCDC113	HG507063.1	FBXO34	CMTM3	TAS2R1	SERTAD4	EAF2	PITX1
FBXO15	ZCCHC6	WDR75	GALE	DDX19B	CLPTM1	ANKRD10	KJ903065
FUNDC2	MLX	CRYAA	PNPLA3	USP28	ZCCHC8	FAM98B	IFNA2
DCANP1	MRM1	ZNF664	SPINK7	CASC2	HLA-DQA1	SCN5A	SERPINB3
GNMT	FAM29A	LYPD6	SLC27A3	ATAD3A	BIRC7	SAP30	FXYD3
MFI2	TRNAU1AP	IPMK	CALCA	C7	FAM173B	ULK3	SCGB2A1
CDK14	FBXW4	RILPL1	ZNF878	CACHD1	ARSK	C17orf105	CSN1S1
SLC27A1	SMARCD1	HSPA8	IGFLR1	SLC3A2	UBA2	DLX4	ZBED1
C9orf142	RAD9B	FCRL4	PPIL1	ZHX2	FLT3LG	UXT	GCNT1
KJ900917	SLC1A2	LHX6	DOLK	DSCR8	C1QTNF5	ZUFSP	TAS2R60
TMEM163	FBXO3	CRCP	SEPT9	RETN	EPM2A	SLC39A4	GPT2
SUSD1	AMOT	KLF8	GKN2	FLJ40194	KRTAP13-1	PIK3CA	WBP1L
CXCR3	B3GALT4	NIPSNAP3B	SLC46A2	POMT2	ZC3HAV1L	HOXD1	TWIST1
COMMD10	NUBP2	TRAM1	AADACL2	KLRAP1	UVRAG	RELL2	BRD2
CST5	KRTAP20-1	KANSL3	SH3BGRL2	RP11-45A12.1	PTH	GORASP1	C19orf84
CD27	PIKFYVE	DENND1C	TKFC	MFGE8	TAP1	NUMBL	SPIB
PDPK1	CD3D	DNTTIP1	IL3	TMEM71	MYB	EFCAB12	PTPDC1
B3GALT1	SLITRK3	IFNB1	TMCO2	CCDC191	IL17B	AKT3	ATXN7L3
CYP2R1	LSM5	PIGG	FKBP6	WFDC1	KCNN4	EPB41L3	MUC7
PRUNE	OR52N4	OIP5	CYC1	TSPAN14	KCTD9	UGT3A1	WBSCR17

DNAJC19	KCNA6	ADARB1	CENPU	ANKRD20A11P	ZNF781	MOGAT3	CYP17A1
CYP2A7	FMOD	ACSM3	PNPLA2	MYDGF	RNF34	UBA7	SLC25A27
FAP	SEH1L	C14orf132	TMED1	LPCAT2	AMY1A	CREB3L2	SLC24A4
OR51B4	RPL7L1	LOC401052	ZCCHC3	IQCG	FAM127A	NCCRP1	ASIC2
FAM46D	FBXL2	CHST8	ZNF771	PIGQ	FAM24B	OR10H5	GALNT15
ARMC1	ABLIM1	PLEKHO2	MRAP	POMC	AMMECR1	SLC5A7	MUC15
AL132660.14	HCP5	TIGIT	CD28	PKIG	PLP2	FAXDC2	FAM76B
CRIPT	TP53TG5	CLEC14A	TLL1	ITGB1	GPATCH1	CDS2	KCNK12
C14orf80	AUTS2	SP140	EIF1AX	NFAM1	MTURN	LDLRAD3	CCDC84
RAB33B	SYAP1	VIP	LSM3	BATF	VPS29	COL3A1	SLC9A3R1
VARS	ZNF350	TMCO6	LIMS1	UBE2N	GOLGA2	DDX6	CRYBB2
NOB1	GFOD1	LPAR3	ASCC3	MBD3L1	SNRPC	MAP1LC3A	GABRB3
SLC8B1	MAK16	HHIPL2	KAT7	MAMDC2	CIR1	PIGU	SOX10
IMPAD1	CARNMT1	MPV17L2	SERPINA5	HSPA2	ORAI2	RBP2	VPS39
PIK3R4	KRTAP21-1	CASP8	HRG	H0Y9J4	PRSS1	PTPRC	CTNNAL1
C9orf72	ZBTB7B	HYI	OR2G3	MFSD7	UGGT2	SNX16	SPINT2
TLR9	AFTPH	DISP1	SDC1	AOC3	ADAM15	DMD	GORAB
DEFA6	FAM106A	AGO4	PFKL	DKK4	PSMD10	DNAJB7	SPACA3
PUM2	S100A7A	HG512367.1	AK293512	ULBP2	TYRO3	CADM1	FGF13
MON1A	TNFSF12	ZC3H10	PMS1	GSTZ1	LRBA	CYP3A4	MYO1C
DBI	PRKACG	SOX12	FAM98A	RP11-128A17.2	KLK11	ZRSR2	CD79A
PCBP2	C8A	TMPRSS11B	HAO1	ZNF333	SYMPK	MRPL47	FBLN1
CABP7	CCM2L	MACROD1	GLIPR2	SHARPIN	KJ902277	KJ902572	UNC5CL
SWSAP1	DEFB4A	CHST4	SFT2D2	KRTAP19-4	SLC36A1	CCL26	HP1BP3
C15orf65	XR_922687.1	CARD16	SPC24	LARGE	SIAE	VTI1A	CCBL1
FAM212A	UBL5	RICTOR	TMEM65	BC003355	SLC4A2	NDUFS2	MSI2
NRBP1	PRDX1	PKN3	PPP2R2C	PAEP	ENDOV	RDH10	BLOC1S4
CNFN	PVRL1	RIPPLY3	ODF3	CNIH1	AC008733.9	NDUFB1	TCEAL2
CCL17	TRPV4	UNC50	ZNF408	DYNC1LI2	MAFG	SSRP1	KRT33B
P4HB	PYCARD	NGLY1	MYT1	DCAF11	ZNF365	SNX2	NPM3
LILRA5	CDCA2	LY6E	ADM5	CD37	ATG16L1	SRR	PF4V1
GPR37L1	RP11-45M22.5	CHCHD5	TRAPPC1	ANLN	FITM1	XCL1	HSPA9
DNAJC8	LRRFIP2	CAPN5	RLBP1	VASN	OSBPL10	PCDHGA10	PAGE5
HLCS	GALNT13	ZNF709	C17orf67	KRTAP19-7	GCNT2	ETFB	ZNF554
TIMM9	NMUR1	MSX2	LMX1A	APOBEC4	HOXA1	FZD5	ACBD4
MVB12A	NAT6	RHOB	B3GNT3	KRTAP3-3	THAP7	KMT2E	KJ900916
C16orf87	SLC5A2	RGS12	SFTA2	DCAF4L2	G6B	JADE3	HTR1E
FLOT1	EPS8L1	MARVELD3	USH1C	PHKA2	NAV2	MRPS34	LSP1
AKR7A3	ZBTB48	NT5C3B	GPS1	MAPKAP1	LACE1	KIR2DL4	BMPER
FAM171B	GPR132	CAMK2N2	RAB9B	RUNX3	PTCD1	RNF41	MRGPRD
TSTD2	BC069228.1	MGC10814	S1PR5	ZNF157	ELAC2	CENPL	CRHBP
CCDC74A	PEX10	NSFL1C	HMGN2	STARD10	THSD1	KCNB1	NAPRT
HG491580.1	LINC00313	HNF4A	VGLL2	GNG13	NTF4	KIAA1191	PLD2
UBALD1	NVL	UBA1	DUS3L	MS4A3	SLC31A1	PRKCSH	ACP2

ZNF79	SIN3A	C11orf87	F2RL2	SLC6A3	ELK4	MYLK	HOXA7
POLD3	KLK2	PRSS21	C1R	GPR171	HSD3B2	TGM1	IGFBP6
C7orf26	CHPT1	CKLF	CST2	TRDN	TLE4	KCTD18	TERT
CMKLR1	AKAP11	LOC100507507	CISD3	AC011504.3	RP11-13P5.1	C8G	TEX19
GRIK3	IL11	PES1	SLC16A5	ERCC5	ACAA1	EFHB	UBXN8
DTYMK	IGSF21	F11R	LBHD1	ZNF555	PRSS3	CTF1	HCFC1
MMP13	MYO1H	KIF3B	TIMM22	SRY	LYNX1	BLVRB	HG509373.1
CYP2C18	TICAM2	GNS	PLXNA1	ZADH2	EFHD2	CPT2	TROAP
MARCO	CTSS	NDUFA2	MALL	SLFNL1	PLA2G10	CHCHD10	PMAIP1
HIST2H2AB	CLCN4	BCL2L10	KLHDC1	LOC101059954	CCL7	TMEM134	NEDD8
MORC2	SRPK2	MX1	IZUMO1	ACTR6	ZPR1	FAM195A	SLC7A9
PPIF	RECQL	PRKG1	COL4A6	AGO1	MAN1A2	TCHP	CAMK2B
SFRP4	FBXL7	TRIM34	MFN1	GYPC	PIP4K2B	RNASEK	SLC26A8
GPR31	ADO	SEC23B	YBX3	SUSD2	DNAJB8	FJX1	RTBDN
LY9	GPR4	KCNJ2	WDFY3	USP33	RNF122	PDZRN4	KCNMB3
TAB1	SLC35F6	KJ900884	DHDH	HIPK4	HOXC5	SLC35A4	TTK
PPM1D	LRRC71	IDH3G	ALG3	M6PR	REEP3	TRH	COL4A5
MTERF4	NEDD9	FAM111A	LILRA4	KRTAP4-2	KCNA1	CCL15	TNFRSF19
MIS18BP1	NQO2	GPR155	CDC45	SLC2A8	SPR	ANXA8L1	SMARCE1
RAB29	XAGE3	KJ904314	HPS4	WDR83OS	BCL3	LYPD4	CALCRL
CYP26B1	SERTAD2	PHACTR2-AS1	TSTD3	AC255379.1	AKR1D1	SERPINB10	LEAP2
ALPPL2	SLC25A22	TSPAN13	LINC00337	LARP1B	GBGT1	DPP7	PUS7L
APAF1	KTI12	CHST12	SLC27A6	BUB3	COL23A1	TMCO5A	ATG9A
LETMD1	DPCR1	ASB4	IGFN1	SERINC2	GRHL3	MURC	FBXO40
KLRC3	IL17RD	SLC38A3	SPINK1	DAW1	FAM20B	TAS2R13	SLC19A3
APPL2	C1orf159	DDX43	RGMB	C3orf33	RP9	RNF144B	GRHL2
WISP3	SOX30	LYSMD4	EDDM3A	CDADC1	CACNG7	SPERT	IPO13
GTPBP3	FHOD1	RBM19	INA	GABRG1	CROCCP3	ETNK1	CALR3
CASP1	ZNF784	HSD17B3	RNF180	CLCN5	MAS1	METTL4	CAMK2N1
TRMT13	LRFN1	ZW10	PBX2	PRRX1	B3GALNT2	BEND2	IRF5
AP3D1	GLRA3	S100A12	SLC7A3	LAIR2	GUCA2B	OPA1	NMRK2
HFE	GNA12	HYPM	SERINC3	SMTNL2	MIR1-1HG	ZNF280B	IRX3
PKNOX1	KLHDC10	MCM4	PYURF	S1PR4	SLC52A3	UBE2B	RAMP1
SYCP3	GIP	LACRT	APOBEC1	HEPACAM2	ACBD3	LINC00116	LN607916.1
GUCA1C	RPL26	SLC35E2	SKP2	CRHR1	TYMSOS	MRGBP	SPATA32
FMNL3	C20orf24	SUGT1	APBB1	SDHB	LATS1	TMEM92	ICA1L
ID1	SLC30A7	RAB11FIP2	FBP1	AGTPBP1	FUT8	POLR1C	MARCH1
DOK6	SEMA3G	ODR4	CCNY	NETO1	FXYD7	KJ901136	CNEP1R1
FBXL14	CLUL1	SVBP	ZBTB5	CHRNA5	ARHGAP4	POLG2	C8orf76
CCDC70	RNF128	RSBN1L	TTC32	RNF152	KJ904149	GDNF	PCDHGA5
PRR34	ZNF224	CEACAM6	IFI27L2	ZNF607	AMPH	HES6	COPZ2
MLF1	PRDX4	TRMT112	HIST1H2AA	CLDN16	BOLA1	GRK5	IL17RE
HMHB1	PTK6	ST7L	PHACTR3	AC013402.2	ATP6AP1L	CT83	KIAA0087
PTGIR	LIN9	C15orf57	ZC3HC1	TIFA	OCIAD1	SDHD	DDB1

ZNF503	MAGEA9	PLCXD1	C12orf40	SLC2A12	GABRD	IL20	CYBRD1
AGO3	PVALB	BSND	HAUS6	ASCL3	ERAS	OAS1	SCP2D1
MAP2	YPEL1	MT1H	S100A1	NME8	TRAT1	CPB1	MZF1
ABHD4	KISS1	OR10Z1	GOPC	ARHGEF10	YIPF4	TTC38	TPK1
HIST1H2BM	XM_008958886.2	PLXDC2	SCRG1	KLRK1	STON1	HG495547.1	NHLRC4
DYNLL1	KCNK4	ZNF621	GPR55	WASF3	SERPINB4	CTSF	ELP5
ACSL5	MBD1	TUFM	SLC25A33	TRIM31	CASP2	TPO	MDM4
HCAR1	VDR	SLC12A9	TMIE	AK074283	TFAM	CAMLG	SNURF
ALDH7A1	PGAP3	GCGR	ADGRG5	PRR7	RCN1	PDXDC1	EDA2R
EXD2	FAM118A	DHRS4L2	BPIFA2	LEXM	SUSD6	SLC38A4	FEN1
CYB5D1	F9	SELE	LINC00431	INPP5F	SGPP2	PNP	LYL1
IL24	KCNMB1	APOC2	RASGEF1B	TNFAIP6	ZBTB32	GARS	CYP1A1
BUD31	TAAR6	RFT1	AKR7A2	LOXL4	PCM1	PLPP7	MZT2B
HIST1H2BA	ASXL1	CCNK	FAM178B	CRHR2	SMURF1	SYNGR4	C20orf166-AS1
ZNF654	MSL3	FAM133B	DIEXF	SENP6	ATAD3B	SLC3A1	NMNAT2
RRM2	MOB1A	ZNF84	ARL5A	CAV3	POFUT2	ZNF565	GHRH
WNT8A	FILIP1	IFNA1	KCNK7	VRK1	SOX13	MRPL21	VNN3
BLOC1S5	NDUFA4	GLT1D1	TCP11L2	KCNIP4	BTG3	TRIM45	LINC00301
WBSCR16	POPDC3	LRFN4	CPSF3L	POLK	MRPL52	KITLG	CLMN
PRPF4B	CTAGE1	IL5	ARCN1	RASSF3	SMPD3	NKAPP1	PAPD7
ST8SIA3	KCNG3	ADAMTSL1	CYP11A1	RPRD1B	CYP4F11	IPO11	IFNLR1
DCP1A	ECHS1	CHST14	ANXA7	CPNE3	SRRM2	LCORL	GLYR1
TMOD2	CALCR	SULF1	CCNB2	RND2	E2F8	HG511691.1	LINC00997
ZDHHC12	ALG14	DZIP1	SERPINB8	CMTR1	COQ2	XXYLT1	ZNF519
APOL4	SPTLC1	DDAH2	FAM13A	ADD2	NEK7	ARTN	KJ902483
LONRF2	AK125151	HDGFL1	SNRNP48	PIGP	PRRG2	OR1D5	VNN2
SYS1	DYRK4	ATCAY	PIPOX	KJ900789	UBASH3B	OTUD7B	HAGHL
SLC18A3	TMEM41A	GADD45GIP1	GNRH2	KIAA1841	CCDC126	NPM2	S100G
GRHL1	NG_011755.1	COL21A1	UBA3	RNPEPL1	DAXX	ZNF672	SLC24A5
TEFM	AIPL1	CALU	COL4A6	RNF170	GMEB1	CCT5	PIAS3
PLA2G1B	COG8	MEPCE	THOC3	WNT9B	GTPBP2	CDPF1	RINT1
IFNA6	KLK14	NCF4	PDE8A	MUSTN1	TXNIP	LOC105377135	NNAT
CASR	RASGRP4	ATP5B	SAMD11	PUM1	SART1	WBP1	OBFC1
DRICH1	FAM214B	PTGES3	RPTOR	ST3GAL5	CCDC121	ARSG	RSC1A1
MMP3	AKAP13	LARP6	IL1RN	C9orf163	LOC729296	NKX2-1	TARP
SAXO1	HMGA1	LOC339803	C10orf88	PCP4	C19orf45	MCM2	CACNG6
LOXL3	KCNQ5	TPP1	ZHX1-C8orf76	KCNG4	ARHGAP26	STK40	LOC102725009
NUP210	ORAI1	PCDHGA8	ZNF74	SLC37A1	HG497823.1	IRAK1BP1	LNP
CNDP1	KRTAP10-7	MGC27345	TMEM141	FMNL2	UBASH3A	TAS2R43	KJ902887
TEKT3	PRAC1	ASCC2	TBL1X	PCSK5	IL1R2	RAB26	GPA33
SULT2B1	TCP10	SCGB1C2	WDR3	ALOX12	PPP2R2B	METAP1D	BTBD10
LINC00346	SLC5A3	RGS6	NDUFA9	SAMD4A	SYTL1	STRA13	NXF2
COL9A3	IGHG3	POLDIP2	IL10RB	HMGB4	FXR2	SSU72	ZNF232
SETBP1	FAM96B	ZFP2	SPIN3	TUBA8	ZNF75D	AMIGO3	MPL

PRKAR1B	FAM231D	COLEC12	ISL1	TAS2R3	MYBPH	KJ900870	XM_014345078.1
ARL17A	TERF2	RILP	LMX1B	G6PD	PLCG2	OCEL1	FBXO33
SAV1	PLAC8	HIST1H3A	LRRC32	CC2D1A	GPR75	PCDHGA11	RABL6
NOD1	ATL3	ZNF710	HG495238.2	ZNF124	SLC10A6	RASSF1	UQCC2
BTK	ABCD1	SNX33	KIF20A	MAN2B1	SMAD6	ZNF200	UPK3B
NDUFS5	UBR2	THEM4	AFM	FPR1	FCN2	BBX	ATG4A
MARS2	BEX5	PRKAG2	PRKAG1	ANKRD26P1	GLB1L2	RARS2	RAB28
RPL37A	NLRP11	MFSD11	MRPS2	SLC22A8	BGN	BAG3	NDUFB10
DCD	ADAMTS18	SLC46A3	OR1A2	IQGAP3	STEAP2	NOS1AP	METTL2B
UBE3D	P2RX6	DMRTC1	RAB40A	ARR3	PERP	C12orf45	CCDC186
NKX2-3	PITPNM1	BEGAIN	MRPL46	CHAC1	ZMYM6	SLC25A26	MECOM
COX6B2	TMEM53	IL2	HIST1H2BB	LIMA1	PTDSS1	PTTG1	NINJ2
ASCL2	ZFP42	LYZL2	UFM1	SAR1A	ELF1	ASB2	PLAC1
PPOX	TMEM79	ZNF720	GPRC5D	TINAGL1	LZIC	HIST3H2BB	DHX32
CIB4	CTD-2213F21.2	KCNJ12	SETD3	LINC00494	CCT8L2	ZNF160	FSTL5
CCDC9	SPSB4	CLCNKB	IER3	MTTP	SH3TC1	SLURP1	SH3BGR
PRKX	OR4D1	LINC01554	PIGA	SPATA12	NG_030306.2	GEMIN2	HBM
ZNF839	TECPR2	SRPRB	FAM206A	CLTA	TRIM28	RGS14	MRPS23
ABHD16A	TM4SF1	ZNF597	ENTHD2	BTNL9	RHOXF2	RPS6KA6	OR5T1
IER5	RAPGEFL1	XM_009435869.1	HDAC10	ERCC2	FAM69A	TCTE1	LOC552889
ARHGAP5-AS1	SNRPB2	NTRK2	TAS2R16	MCHR1	BC034142.1	TBCCD1	PAQR4
TMUB1	CA11	SLC39A3	GLB1L3	LGALS13	LPCAT3	DOLPP1	LARP1
CYP19A1	MSL2	PGBD1	PLAC8L1	AL137795.10	BPESC1	ANKS4B	GPM6B
TM2D1	SPATA24	TM6SF1	ASPM	YBX1	USP49	GPR157	ASB1
GPR108	ABHD14A	VTCN1	ZNF28	ADCY6	CCL28	WDR76	GLP2R
UBE2L3	CCDC58	PYY	PTMS	PMF1	LINC00482	FGF5	MAPRE3
FCER2	SEC16B	JADE1	MCM6	JAKMIP2	MAP3K5	USP30	MAP2K4
KLRG1	CYP4F12	FOXI1	BRWD1	CTDSP2	FGF10	OMP	VAMP5
CRYBA1	MPC2	SNTB2	BCAR4	SESN1	C11orf94	GDF9	ILDR1
ARHGEF18	CELF6	CTNS	GABRQ	C11orf71	SEZ6L2	FOXJ1	CHCHD4
CSTB	SERTAD1	ADCK3	YRDC	MLLT6	CFC1	KAZN	RASA3
LINGO2	KLC4	HG501992.1	PATL2	SCT	SEMA6D	SATB2-AS1	KJ904347
GTF3C2	TTF2	ABTB1	ACVR1B	GNAL	TMEM115	UTP11L	C2CD2
MYO5C	RNF166	P2RX2	ADAM21	TSPY3	TICRR	PRPSAP2	DCK
WNT2B	ITIH1	WDR45	LIF	STX11	SVOP	PALM	PCDHB12
GSKIP	RBM12	ITFG2	PEX14	CORO1B	COMTD1	SPHAR	KLHL6
JMJD6	MAN1A1	OCM	BPIFA4P	LOC729956	CTBS	CDRT15L2	ALG13
ANKRD45	TSR1	SGSH	ERMP1	TRAPPC5	STX3	UBE2S	AGMAT
NACC2	TRAF7	VTA1	APITD1	KJ904142	PDE6H	VEZT	SCP2
FDCSP	SH2D4A	TAF1	PRDX5	FAM134B	RBPJL	OGFOD2	MS4A2
RGN	G6PC	CNP	IL13RA1	XKR8	MICA	FGFR4	EGFLAM
TRIM62	TEX28	GPAM	MXD3	TMED9	FNDC5	NDUFA11	KHDRBS1
PRF1	TRIM60	DTX2	SLC44A3	PTPN12	GIMAP8	KJ901268	CCR3
TNFRSF18	ZNF319	ACAD11	RNF216	TAS2R7	S100Z	ZCCHC14	LINC00471

INHBB	TAS2R40	MCFD2	C4orf22	JPX	NLGN4X	EXTL3	CLSTN3
MTMR8	UPK3A	DYX1C1	MAGEB3	LY6G6C	ANKRD37	SOX9-AS1	HRASLS2
ALDH8A1	C7orf50	PSMA8	MAP6D1	BAIAP2L2	NUS1	RGS2	BPIFB1
DARS	WFDC11	DEFA5	CHAMP1	CAMKK2	LANCL2	SIK2	GATSL2
HG499438.1	COX18	EMC7	USP39	TGFB2	AL133163	GNB2	DNAJC11
WSCD2	BTBD1	TFEB	CDH12	CLIC4	BOP1	HSPB7	MMACHC
CDCA7L	PPWD1	HBD	MRPL14	C1orf174	DSCR4	OR11A1	KJ903280
RTP3	NT5DC1	ERICH2	ARL9	AHR	HG502761.1	HIF1A	SRGN
PRM2	RIBC1	CT47A1	HOXB7	TAS2R14	DHX16	TMEM42	OR4K13
SENP2	RARRES3	BLOC1S1	ERVMER34-1	PMP22	PADI4	CDRT4	ZNF436-AS1
OR3A1	BBS7	TRIM59	HSD11B1L	NRIP2	UPK1A	HOXB5	FER1L6-AS2
RORB	TRMT1L	PAH	DRAP1	FAM120B	HBEGF	CHGA	VTI1B
SERPING1	MED24	APLN	HTR2B	FBXO24	ZNF354C	KCNK10	MSMO1
KJ901255	CNNM1	TNFRSF13C	ATP6V0E1	AC104057	KJ900954	RXRA	TSC22D4
SLC2A3	ZYX	TTYH1	SRCIN1	WNT5B	SLC43A3	STAR	STT3A
SCGB2A2	BC064144.1	FARP2	CGGBP1	KJ900906	MAP9	ATP6V1F	NACA
DGCR2	TCEAL7	SPEM1	TMEM178A	SLC7A6OS	TONSL	CLEC2D	C21orf2
MRGPRX3	C19orf68	UPP1	TRIM55	IL26	BC011742	FANCE	OR1N1
HG497681.1	LHX2	SEPT7	R3HDML	CMTM5	WDR31	INS	WDR88
DHRSX	ANKRD50	HDAC6	EMC8	PPM1G	AARSD1	CCNDBP1	HSPB1
RHOXF1	ANKS6	LAP3	WDR55	PURG	SLC12A6	IFNA16	FGF23
TBC1D10A	DLGAP4	ZNF792	MORN2	FAM86C1	KIAA1468	SLC45A3	KJ904380
FSCN1	PSMD5	FAM69B	GTF2H2C	LRRC46	GDAP1L1	WTAP	NANOGP8
GAB1	LAMTOR5	GPX2	COX8C	MKNK2	URI1	NLRP3	DSCR8
DNASE2B	RNF40	PEBP4	LRRC45	CCDC120	TMEM18	FYN	ACKR2
ATP6V0A1	FLII	WDR59	ZNF785	PACSIN1	KLF1	CENPT	MRPS27
MXD1	KRTAP4-4	PRICKLE2	EXOSC7	HIST1H1A	ERP29	HOXC6	ZDHHC15
MPZL3	ITM2A	GDPD2	MSRB3	RBBP8	TMEM259	CISD1	GPR34
FAM107B	STARD7	PSMB1	ADAM33	IARS	ARRDC2	VKORC1L1	SLC37A2
ASIC1	SLC41A1	GAB3	NSRP1	CRYM-AS1	ADCK5	TMEM60	PLAGL1
CMIP	GPT	C4orf33	DNAJC4	ZBP1	GPR84	MFSD12	C4BPB
CPNE1	APOBEC3H	ZNF415	SCAF8	EPHA3	TRIT1	ARID5A	SSH3
TAB2	GANAB	LINC01465	BTBD11	ZBTB39	SPOPL	FOXP3	ADSS
AKR1C3	TIMM8A	BC031228.1	SLC7A14	CLN6	EDEM2	DDX3X	COPS8
VASH1	SARAF	IFNA21	MED27	TTC27	IFNA8	TNFSF15	DHRS2
EPS8L2	IRX6	TTC8	CAT	ZIM2	ZNF528	RILPL2	HEXA
C3orf35	APRG1	DEFB121	SPRR2G	TTC17	IGLL5	ZNF354A	PCDHGB6
CASQ1	CHURC1-FNTB	THOC7	CRTAM	ARHGEF1	RIPPLY1	VMA21	GABRA6
FAM104B	UBL3	ATN1	C2orf88	ZNF626	C17orf58	NKX2-8	OR6C1
C4orf36	ZNF490	PPM1N	AK091027.1	NR2F2	PDCL2	TPRG1	LTB
CALD1	IGFBP1	ASXL2	CCDC14	CEBPB	IPO5	TRAPPC2	TMEM239
CBLN4	CDKN2C	MUM1	TSPAN4	IL4R	OR4K17	RND3	SET
PHACTR2	PLCXD3	TMEM64	SCAPER	TLR8	BC126936	FLRT3	KJ903673
PRDM1	JHU12445	BANF2	SPANXD	ATP13A2	IL9R	RAB1A	LPL

ALG10B	LUZP2	PLK2	AMOTL1	ALKBH4	SPANXN4	SPAG11A	C19orf60
ARMC2	DLX6-AS1	GPRC5C	CPNE2	HIST2H2AC	KIF4A	PAM16	SPINT1
QARS	NEUROD2	BCL2L2	KJ900973.1	CAAP1	GBAS	OR6C76	CCDC181
SYT12	INO80C	ZNF137P	C14orf28	ZNF514	TMEM2	SUFU	FBXL6
BAGE	NF2	SPRR2F	SPRR2B	FAM179B	FECH	SPRR1A	PGAP2
DBR1	USF2	KRTDAP	PSMD7	DPYSL3	AGL	LHX4	ZNF653
LMOD3	STAG3L2	TBX22	GPR12	MCAT	AHCYL2	SLC6A4	TAF1A
PAXIP1	ARPC3	PAQR7	NRBP2	STUB1	NSL1	SPAG11B	IL13
RASA4	TMEM185B	HYPK	MYL6	CES2	SCARB1	PGD	OTP
RPL17	CHRNB3	PMVK	NDUFA7	CHIA	PITPNA	MMP7	FCRLA
EPS8	TIMM21	SEMA6C	ABL2	GPI	RBP4	HOMER2	DPH5
PACRG	GLUD2	UBE2DNL	FAR1	APOE	CPPED1	ASUN	CFAP36
KIAA0319L	ELOVL4	TMOD3	ZHX3	EIF2AK1	ZNF599	LOC101928917	ZFP3
MLYCD	NG_013420.2	TBC1D9B	GPR143	FA2H	E2F3	OPTN	RGS16
SF1	ASF1B	PSMC6	SCN4B	PUDP	MBP	SPRY2	SELT
THBS1	CLIP4	ACSF2	Y15228.1	NM_001131261.1	CPA4	INF2	IVD
ATP6V1G2	SUOX	TPSD1	LN608954.1	PDS5B	WRAP73	STKLD1	C6orf99
SLC25A29	MCM8	MPP3	PELI1	MAPK8IP2	ZBED3	C12orf49	SUMO4
DIRC2	LRRC10	RTFDC1	ZNF213	KCNE3	OR6C75	POU3F2	UBB
POU1F1	C3orf56	SPRR3	HMGN2P46	AC090195.5	NUBP1	WWC2	NEFL
COPS6	PTPN7	PI15	GSG1L	PFN4	C8orf46	SHMT2	PPM1F
DDX54	RERG	AP2B1	FBXL12	SMR3A	C2orf76	RP5-842K16.1	MCM10
ZCCHC9	ASRGL1	KJ901271	LINC00858	LYRM7	C1orf115	ESD	SIGIRR
VAX2	CYHR1	KRT13	MS4A8	TRMU	PDZRN3	TP63	HOXD3
GMFG	EVI2A	ZNFX1	CRX	WWP1	ZNF496	POLD1	MBLAC1
YB003	SIN3B	CRYM	LSM10	BCCIP	ABHD16B	FIG4	PDHB
KJ901160	TRIM34	FAM180A	RAD17	C8orf34	GRAMD4	TMEM156	CRIM1
MT1DP	ICA1L	C1orf120	NO66	GRIA2	KJ903709	WDR91	UCKL1
APOA5	GLRX3	EDC4	ATP6V1G3	MECP2	RAB24	C5orf51	GDPD3
BEX4	OR4C15	DVL1	ADAD2	JPH4	LOXL1	BIK	SAC3D1
CRELD2	QDPR	AAMP	WDR37	PRKACB	TADA2A	FUK	CLEC3A
TTC37	APRT	TOR1AIP2	KANK4	NOXA1	ASPN	CLIC3	KJ905803
NLRP1	N6AMT2	NBPF1	CLHC1	NUB1	R3HDM4	ALDH3A2	RASGEF1C
TNFSF13	SPINK2	AC100793.2	FABP6	ZNF268	GREM1	CCDC137	RASA1
ARAP1	PRAP1	SIX5	DLG4	LST1	C12orf75	AZU1	CLSTN1
PSMD14	APOBEC3F	BAGE3	WARS	YIF1B	NUSAP1	SLC25A28	TEX261
LOC101929465	SCARA3	KJ904202	PNPLA6	ACAD9	TBX3	SAT2	THAP6
KRT16	C9orf114	MYBL2	IFNAR1	LINC00336	VSX2	FAM133A	BC132847.1
PRORSD1P	PITX2	NFKBIZ	CCDC103	NACA2	KCTD6	KJ901517	KIAA0556
KRTAP1-5	PACRGL	RUNDC1	YTHDF3	FOXA2	PTGER4	RHOG	TRAF4
SLC30A9	ATP6V0D1	XM_009447605.1	MUS81	ECI2	GLCE	OR6K6	HOXC4
SNX20	SEPP1	RIMS3	KAAG1	SLC51A	CARD19	EMC4	TCN1
FBXO9	HEMGN	KLC1	GNPAT	TTC7B	BPY2	RAB11FIP5	FRK
CRYBA4	RPRD2	TIAF1	TRIM24	RIIAD1	CPLX2	GPR78	PANK4

MGEA5	TBX2	AOC1	BST1	FAM110D	ERICH1	CXorf67	C1orf94
NLK	CBY1	TRPM3	MREG	NUP35	PDE1A	TNFSF4	MANF
ZNF540	CDH8	ZBTB26	LCN2	C5orf30	SPANXN1	C9JAW5	C2CD2L
PCGF3	PIGN	SH3GL1	PAMR1	TRMT44	HOXB9	PCDHB13	BAGE2
KJ904294	OR2T2	VAC14	RNF20	TMEM199	PHAX	SPANXN5	CSDE1
EPC1	OR2L13	HLA-DMB	CIB1	POLH	C19orf73	SCGN	HMGB4
CAST	CLDN7	ZNF547	POGZ	SYNJ2	EPAS1	FBXL8	CD8A
C5orf34	C1orf74	GPIHBP1	LRRC40	RAD52	EGFR	FLVCR1	IDS
PFN1	KCNN2	MYEF2	GALNT9	TMEM189	YO001	HSCB	AC240719.1
TTLL7	IRF2BP1	OLFML2A	SRC	NRN1	CHCHD7	KRT7	CYB561
TMED8	NBR1	MAP7	AF080246.1	TYMP	PKN1	PHB	CYGB
NAP1L5	LOC345576	EIF3C	SLC10A4	PSG3	ETV5	ZNF608	SLC25A43
C20orf144	PIK3AP1	ZNF658	YA043	BMP3	INHBA	GLIPR1L1	DPM3
CTD-2126E3.1	HERC3	SLC25A15	PAQR9	NIPA1	TBC1D26	GATAD1	MAPKAPK2
PGLS	GDI1	ZNF324	LRP3	NOLC1	ZNF468	MFAP4	ASAP3
BGLAP	DGKZ	RPS18	SRSF9	TNMD	SHISA5	FAM213A	PLAT
NET1	ZNF57	MGC26597	TULP1	PIEZO1	USP48	TPSB2	VPS37B
TTI2	LRSAM1	ARL17B	COA6	AK127903.1	ITCH	AQP1	FAM86B1
RPUSD3	LN608403.1	CDYL	IRF8	LAG3	KIF1BP	CRLF3	DDX21
TRAPPC3	YBX2	DHH	KJ904287	SRP68	F11	DENR	SIX1
ANKEF1	ZSCAN2	COLCA1	HGF	IL11RA	MSTN	IL17D	DNAJC5
ABHD12	C5orf45	ASNA1	TPST2	IFFO1	SLC25A14	CCBL2	OR8H1
SGCE	OLFML3	ATP9A	LCE1A	BMP2K	NR5A1	FOSL1	RAB11FIP1
HTR5A	TUBGCP5	COMMD3	ALDH3B1	XM_004049765.1	MIR99AHG	JHU12892	ZNF789
SV2B	HCG9	MTFR1L	CCDC92	RAB4B	HG493596.1	GATS	FCF1
TNFSF14	HTR3B	STX16	CFD	MST1	CYBA	HADHA	KEL
GZMB	YAP1	FARSB	SRM	TNFAIP8	OR6C3	NLGN1	C7orf76
YS025	LMO1	ABHD12B	TMIGD1	MID1	TP53I13	C3orf36	NAT8L
BRPF3	NOTUM	KJ903429	PEX1	TINF2	FERMT2	VPS35	GXYLT1
MIER2	SLC45A2	LYAR	DDT	KJ904403	SLC19A2	HPS1	FN3K
SPIN1	UCP3	FANCB	ACSL4	RNF121	IRAK1	ZFP90	AJ315540.1
MUC1	COX7B	PTGER1	MTHFD1L	AQP7	GOLPH3L	ZNF773	ABCG1
RNF144A	SLC26A7	COMMD4	DDX25	TFAP2D	SRSF8	IQCH	FEM1A
PRR18	CCR6	CENPP	SLC43A1	OR10K2	PRKCI	ADRBK2	ZNF436-AS1
C1orf220	PDGFA	PRSS37	TIMM8B	TMCO3	MGC39372	CST6	MT2A
SPRR2A	TAB3	DDA1	ARID3B	SLC30A6	GSAP	SPG7	BPIFB2
MORC2	PDLIM2	TCL6	PDPN	C9orf47	MMS19	NR4A1	MMAA
C16orf57	TPBG	ARSA	TRAPPC9	PCLO	DOCK7	CRYBA2	ZNF567
NEPRO	PSMC3	FOXN3	BOK	C22orf15	CXCL9	MAP1LC3C	LOXL3
KJ903167	ERBB2IP	MBOAT7	AAAS	WNT6	EFNB2	HLA-A	ATP5G1
GSTA4	CARNS1	ARHGDIG	KIF14	PALD1	SCNN1B	ZKSCAN4	IFNA4
NFAT5	BC046095	KJ903745	NDUFA1	ACTR1B	CDYL2	METTL22	TCF25
TCAF1	CBFA2T3	PI4KB	RANBP10	RNF4	ERMAP	FAHD2A	PPP1R16A
FAM63B	KJ903245	BRAT1	PAQR8	GRN	ACBD5	MPZ	RUBCN

EPHA10	CCNA2	TIMP3	GDF15	MGP	AK055694	AK127387.1	FDX1L
PABPC1L2A	MUM1	ECE1	STXBP5	SZRD1	KCNK2	AY312371	NTRK3
MATN2	HS3ST3A1	PLGLB1	OR2C3	RXRG	SCAF4	XKRY2	AF100747.1
MMP1	SPDYC	HGD	HTR7	CLEC12A	GPR156	CENPB	LRRC14
SMG5	ABHD8	MED16	LOXL2	INE1	TBX21	OR11H4	ADAM32
SLC22A2	CRYGS	HIST1H2BD	SNAPC2	ING1	ZBTB14	PRPF38A	SSH2
RIT1	C19orf44	GRAMD1C	UBQLNL	ANAPC2	CLDN6	ILK	GINS3
SCMH1	YR005	MCCD1	BC132980.1	SLITRK6	ZFYVE27	EIF4B	GSN
DNPEP	ZNF677	RPUSD1	PAF1	BC002963	ZCCHC2	EPHX2	CPSF6
MUC21	LGR6	FNDC9	KCTD16	SHPK	KCNQ2	ACOT11	SMYD5
RGS3	UTS2B	KSR2	KIF12	ABHD14B	IL17RA	RASAL1	PDF
WT1-AS	TFAP2E	PKDCC	OR13G1	ENPP5	TRGC2	NELL1	RPL22
CSNK1A1	TGIF2LX	SZT2	DBF4B	ALOX5	WFIKKN2	NAA25	ZNF630
PYGO2	GRIN2A	ZWINT	DUSP13	SEMA3F	RAB11A	SFMBT1	SLC38A10
POLR2J2	NFU1	TRIM38	CCDC190	CDH26	MBD3	FAM21A	BC009991.2
NCBP3	BC037295.2	KIAA0825	C1QBP	MAP2K5	TRADD	ANKRD32	LINC00525
SPG20	SECISBP2L	ATP2B4	DVL3	VSNL1	DIP2C	C2orf44	CYP26A1
KAT2B	CTSZ	DMPK	CELF4	RASGRP2	ROBO2	OSR2	FBXO28
CASP10	CYP7B1	MFSD5	MID1IP1	ABHD13	LTC4S	NDST4	ST6GAL1
FAM109B	TRIM65	ZNF512B	FAM160B2	CA5A	SIRPA	IL31RA	CMTM1
XM_009441031.1	FAM181A	RAPGEF3	NAA40	ZNF766	NPLOC4	SRSF2	YBEY
AK127732.1	SAMD12	OTUD5	CLASRP	SMC1A	ACOT7	PYDC1	SLC6A19
POMGNT2	DPM1	PLEKHH3	MPZL1	AK094777.1	CENPQ	ROMO1	DCT
GYPB	AK097058.1	TAF7	TUSC5	POLB	CGB2	PSG11	NELFA
CAPN10	UACA	KCTD12	ZMPSTE24	SELM	NFATC3	ARHGEF7	LINC00649
TPD52	KCTD21-AS1	SLC6A11	DLST	ZNF133	CWF19L1	INF2	KCNK6
PVRL2	CPB2	BC007394	MDM2	HK3	MFAP5	ANXA2R	MED23
P2RX5	CASP4	SLC17A4	ZFP1	CLTB	ARID3A	ACSL3	TLE3
COX14	HEATR3	NKIRAS1	DDX59	CHKB	GFM1	CBFB	IFT140
ZBTB12	PDIA5	RFK	METAP1	AK125266.1	AVPR1A	TMTC2	FOPNL
FAM101B	NFATC1	ERN1	AY312367	GNG12	CPT1C	FAM129A	NUFIP1
EARS2	MEF2B	GLTP	LEMD1	UGDH	TULP4	RNLS	CKAP2
AC074325.7	LIN7A	FAM210A	LUZP1	WFDC3	ZNF655	ALDH16A1	FAM160A2
PPP1R12B	SLC6A16	TUBGCP4	XPR1	TMEM39B	PRAF2	PSCA	HG501143.1
HECTD2	NOSTRIN	HYAL1	ATOX1	SPANXN3	HSDL1	ZNF549	SPATA5
VTN	LMF2	CREB3L1	BIRC2	MTDH	SLC15A3	TMC7	ZNF385C
BDKRB2	GTF2A1	IGHV7-81	NUP107	TSPAN16	AES	C11orf88	GAS2L1
DECR1	OR11-68	GPR85	L3MBTL4	PPP1R32	PLSCR3	ITGB8	ZNF614
TAPBP	ZNF446	OR4K1	BBS12	ADRBK1	PKLR	KLK8	HYKK
EFR3A	FBXO32	GRPEL2	RP6-109B7.2	LBP	DOK3	CAPRIN1	FCMR
SIPA1L2	ZBTB22	FAM89B	BAHD1	CDC73	MBTPS2	VDAC2	ZFYVE9
AIFM2	ATIC	METTL10	VASP	LINC00173	KRTAP5-3	TTC1	IGL
UBE2G2	ANAPC11	BTNL3	AL035684.25	ODF2L	IGSF10	UCP2	LAMTOR1
TRIM14	CWC25	NPDC1	ASAP2	ZFP36L2	ZNF182	SLAMF8	CTNNA2

GFER	USP47	CXCR5	MTMR3	LEFTY1	STK11IP	MARCH2	PPP2CA
HTR1A	DPP8	RAB5B	RABAC1	DYDC2	HEXB	AK127233	TMEM196
GRIPAP1	ATOH8	KJ903660	CCDC150	SDCBP	MFRP	DAAM1	HCST
EDEM1	ANXA9	ZNF34	TRIM25	LOC146880	NPHS2	WT1	ZNF777
CTSH	CARD9	YPEL2	AK128288.1	HIST1H2AJ	TNFSF10	WDR34	OR2D3
SH3GL2	KCNE5	MRPS28	CSAG1	H2AFX	RPS6KB2	NRGN	TBL2
TXNRD2	KCNJ10	ZNF274	NHSL2	TRAK2	PMS2P3	ADCY9	STAT5A
ZNF576	CHPF2	SRRT	TESK2	BAGE4	HHLA3	GNG8	C1orf137
EIF5B	FPGT	HABP4	PTPMT1	XRCC2	MAL	POU6F1	LY6G5B
TMEM89	CPT1A	RALGPS2	TCIRG1	KJ901204	ZNF561-AS1	HTRA4	PEX6
PAGE1	MYOM3	HDGFRP2	FMR1NB	CPEB4	SPIN2A	EIF4E3	ATP5D
IDNK	OR9A2	GALNTL5	AUP1	ZNF93	SHOX	WDR13	MRPL55
ZNF287	PXMP4	KIRREL3-AS3	MCOLN2	WNT4	NRIP1	WDR66	SH3BP2
MMD2	H3F3C	CACNB4	MTCH2	CCL23	SNAI1	NLGN3	CCL18
CSF3	HOXD10	FTSJ2	KIF3C	CWF19L2	WISP1	ITPKB	ZNF697
KJ903813.1	UQCR10	LINC00470	HMGA2	SLC12A8	SFI1	DTX1	NUDT4
PCDH1	ERLIN2	ZC3H11A	WDR82	TCL6	LBX2	NXN	WASH1
DCTN1	LINC01315	HMSD	VPS26B	MROH8	ELAVL2	AKAP14	KCNJ14
LRRC47	TRGV3	OR8B12	TSEN15	TLR7	SPATA5L1	LINC00885	TMEM211
PROP1	QRICH1	AC048382.7	RASSF2	NEIL3	DNAJC22	AY312370	MT1G
LINC00544	MAGED1	MSTO1	LACC1	MEI1	C8orf58	PRKRA	CTIF
KLK12	MARK2	TOMM22	FEV	UGT1A9	ARHGAP9	STAG2	SUN3
SPA17	ZNF764	HEXDC	HOMEZ	BMP10	ZNF189	RNF8	MRPL12
MOB4	ZNF702P	SF3B1	SLC43A2	NHLRC3	KIFC1	NUAK1	ADSSL1
DEXI	ASCL1	PRKAR2A	KLK4	TSPAN6	ANXA13	GSR	ZSCAN25
DNAI1	ACAP1	TRIM22	FLT1	C1orf186	SNX14	KJ900882	FAM117A
GLTSCR2	HIGD1A	KJ903453	PSMD14	DOHH	TOX4	SLC29A2	GCH1
YAF2	CPNE6	TXNDC16	MTMR4	TRPV2	CFAP126	MOCS2	FXYD4
POM121	GNAO1	PART1	PRRT1	MRPS30	TRAF5	DCST1	PGM5
ACVR2A	ZNF574	RBM17	DCN	C16orf47	C11orf31	OSBPL8	RNASE10
CUL1	LOC101928327	TNNI1	C10orf99	MIR22HG	RPS27L	SYDE1	ZNF513
KLHL10	RPA3	XKR9	PLA2G12A	TXNDC8	PATE2	NAE1	RER1
ZNF280D	PLEKHG4	TMEM207	JHU11996	KIAA1429	ADGRE3	EPM2AIP1	FAM57B
TMEM108	MALT1	GCLC	CS	FASN	CCT6B	C12orf60	UBE2D2
DYNAP	CD40LG	NXPH2	TMEM35	ZNF75A	FAM92B	DHX8	HOXD8
TNIP2	KJ900826	MSRB1	IGSF3	COX5A	MEF2C	BST2	SWAP70
CDC14A	CYP3A7	UBTD1	COG1	TEX43	C1orf189	KJ901963	SLC29A4
KIAA2013	ANKRD44	FSD1	SRSF4	KJ902438	HGH1	DDX26B	CTSL
SNX9	MMD	PSD	RELB	RXRB	LOC440337	TCEAL4	GPATCH11
DPPA4	TMEM133	GATA5	ADGRG7	LRRN4CL	TMCC2	RRP12	ZNF585A
DEFB132	SMPDL3A	TRIM61	ZNRF1	RBM46	BC035666	UCN3	SAA1
CRISP1	ABCC13	MARCH6	GIGYF2	RAB42	ATP6V0E2	MRPL52	AP3S1
TEX10	MAGEA6	INPP5E	SLC2A9	KBTBD6	BC132930.1	EDARADD	ATP1B2
BC084559	GET4	TECRL	TRPC4AP	ALG10	PLXDC1	HTR1D	CITED2

LIMD2	ALDH1A1	TIAM2	GAA	UBE2J2	PPP3CB	NDUFB8	ZNF518A
ZNF48	SERP1	DIS3	CCSAP	STX1B	VIMP	IP6K2	PPP1R10
CNTNAP2	HBE1	CCDC88B	CIPC	SLC41A2	RAC2	FAM193B	CDKN2AIPNL
FARP1	C4orf32	VPS41	ATP2C1	HMOX1	PCSK7	PCDH10	TRPC5
NPSR1-AS1	ICAp69	TNFRSF21	D2HGDH	ZNF69	GSTK1	PREPL	FBXO27
FGD2	SNRPD3	GGTLC1	GAB2	SLC8A3	GRINA	AKAP5	COPS7B
ZNF623	DUSP15	ARHGEF19	CUL4B	SMPX	WDR24	OLIG1	GAN
CNTF	LAX1	CENPA	PDCD2	RPP14	NGFR	POLR2L	TPTE2
C1QTNF3	KJ904225	AK056246.1	LAPTM5	C1S	FOXL2	GPX1	C5AR2
IBTK	SNTA1	GSTCD	STK32B	COMMD9	CAMK2D	CDH1	PTEN
BLK	DPPA2	LOC400997	PMS2	DEDD2	LAT2	LSMEM1	TOMM40
SLAIN2	PRSS12	SLC38A7	MOSPD3	DHCR24	SLC35D3	NSUN5	FAM234B
CCAR2	MIR99AHG	THPO	NFE2L3	GRIN3A	RP11-20A20.2	TEX38	UBE2E1
LIPG	GAS6	GABBR1	ZNF689	OXCT1	DCP1B	YTHDF1	CDKN3
RUFY1	HOXA11	TXNRD1	ZNF138	RNF207	ART4	LRRIQ3	LY6G5C
TRIM36	IQSEC1	AGAP2	ZNF85	PPIL4	SLC25A4	MAOB	SCD
HOOK1	FUT6	AGPAT1	LINC00523	KBTBD12	FAM153A	PCDHAC2	ASB7
TRIM72	BCL6	U633A	NELL2	GTF2IRD1	STMN4	DIO3	ZNF416
VANGL2	PITHD1	ACADVL	NANOS1	FAM177B	NIPSNAP1	YP007	ADAT2
ARL4C	BAGE5	TRIM40	ANKRD36BP1	ESRP2	WWTR1	PIP5K1C	LRP11
EEF1E1	RIMKLB	CFAP46	MLANA	IL19	UBXN2A	GALNS	CMTM7
FAM110A	ITGA5	COX10	NDUFS3	ALDH9A1	RMND5B	EXOC5	TDRD1
ZNF256	PSMB6	NUDT10	STXBP1	C17orf51	PGP	HDAC7	ZBTB46
SH2D1A	C18orf8	EVA1B	EPHA7	ZNF295-AS1	SPATC1	ARMC5	CNR1
NRIP3	SMG9	CARD17	DDX58	ZCRB1	TERF2IP	NUDT9	HAUS7
SLC2A13	GGN	ARRDC4	PSEN1	LRRC1	RPS25	BC006271	RHOD
MARVELD1	SLC6A17	RND1	HIP1R	GSK3A	ZNF263	AAK1	MSI1
ECEL1	RAB7A	LAMC1	CAMK2G	ANKS1B	CPA6	UQCRFS1	SELL
MAT2A	CDV3	FLJ33360	DNAJC15	GDF5OS	AC096559.1	HNMT	LEG1
KJ901523	PER1	ICMT	C21orf66	GHDC	MSANTD2	TLX3	CHRND
CLIC1	ADPGK	UTS2	S100A2	ZNF582	ARRDC1-AS1	TMEM192	FAM126B
CYP27A1	RNASEH2A	BRCA1	TCEB3	EPN3	ADHFE1	BLID	BMPR2
WAC	CDC40	AC017104.8	TMIGD3	MON1B	MGC45922	LINC01549	YAF2
ST14	FADS3	FAM134B	LINC00612	SLC12A4	PSMG2	PPP1R27	C14orf37
TMEM87A	E4F1	SLC35E1	AC093668.4	TRIM61	COX5BP4	SLC33A1	ARL10
ANKRD17	NDN	EPHB4	ZNF473	NEUROG2	TMEM8A	ARPC4	ZNF81
TNFRSF10C	ATOH7	STK17A	HIST1H2BH	RPS28	VAX1	CLEC9A	HOXA9
TSPAN9	LRRC55	SLU7	RASGRP3	DCUN1D5	RNF40	CERS1	REEP1
BCL7A	PPP2R3C	SMARCC2	GDAP2	RAC1	TBX20	LOC401281	CRYGD
MYO1E	DNASE1	GATAD2B	LST1	COMMD6	TEX30	SRF	KRT34
OLIG3	SLC10A5	PC	LCNL1	SREK1IP1	TAPBP	ZBTB7A	MYOD1
RHBDF2	ELMO2	AK023277	KCNJ6	TAF10	PODNL1	LCA5	TARBP2
VRTN	RHBDF1	TNS4	ADAL	CCRL2	TMEM82	PARK2	DUT
SRXN1	AK125177	AP2S1	IFNA10	SYP	STS	BCAS3	AL139378.15

JMJD7	LY6D	EBF4	DEFB108B	KU-MEL-3	C8orf16	SCFD2	SLC25A1
CYP4X1	BCL2	ISM2	SATB2	PLSCR4	ZDHHC1	SYPL2	ACACA
SPRYD7	TP73-AS1	PANK1	EED	MFNG	PXMP2	DEAF1	TBCK
SSNA1	SERP2	RPAP1	UBL7	AKR1C2	NCL	NRG1	ANKRD36B
AF332235.1	RSAD1	EPN1	PRRX2	ID4	SMARCD3	SBSN	OR51M1
MSX1	IL5RA	ZNF606	TIMELESS	PKNOX2	PINK1	LAGE3	OXA1L
ZFYVE1	POPDC2	GPR65	RPL39L	NRP1	MYCN	PPP1R2P3	PREB
P3H4	HSPB6	MRGPRF	HAPLN1	CAND1	LZTS3	STK24	STAT4
CALB2	HAUS3	GTF2E1	SAMD13	RDH14	RHPN1	SIGLEC8	SELK
SEPHS2	MAST2	FSBP	URB2	SLC39A5	MOSPD1	C16orf73	CCDC80
ANTXR2	DHTKD1	TM2D3	CCPG1	PRKCQ	LRP10	ANKRD54	ADIG
LIME1	GALC	ANKRD6	LMAN2L	TRAF1	MAP2K6	LINC00242	LYRM5
IFIT2	GCSAM	XM_004037862.1	MPV17L	MYBBP1A	RAB6B	SHC3	TRIM15
PLAC9	MIEN1	OR51G2	NMRK1	BC063803.1	IL17F	LHFPL4	PCGF3
PRPF31	RBM39	SREBF1	STK25	FUT10	PIM3	MAFB	NDC80
NTF3	TRIM16	GPR39	GPN1	NUDT3	AC092535.3	TCHHL1	PLOD2
NTM	YY1	NUDT8	POLA2	NDRG1	LRCH3	IRX2	OTULIN
ZSCAN31	ELF4	LRRC74B	RBPMS2	POLD4	LOC105372840	ASPHD1	NRARP
AGAP1	MAN2C1	JUN	RPP25	SPATA20	SPATA41	ASPDH	TCEAL6
OR52E8	DENND1A	MTX2	CXCL8	AMTN	RFXANK	ZNF783	KLHL9
L1TD1	SLC39A10	POLR2G	FAM222B	TMSB4X	C21orf62-AS1	MAST4	PRSS54
ATM	GNAI1	FAM102B	CTCF	MDH1	FAM182A	HIF3A	TOM1L2
DENND6B	TSKS	PRKCDBP	FAM220A	GRWD1	MED4	TOP3B	DDR2
IGF1	CT45A1	KDM4B	DEF6	AK057677.1	MTA2	UMOD	SEPW1
HTR3A	IL4I1	ZNF592	ISL2	C17orf47	RANGRF	CBL	TXNDC12
HCLS1	HIST1H2BJ	HIST1H2BO	RUSC1-AS1	PCDHGC4	TLR1	C12orf43	ESR1
SSC5D	CENPH	PTOV1	PPP2R2A	CHTF8	PIGS	OCLN	AC209618.3
MIER3	UCMA	BX255925.17	PTCHD4	SYT1	WDR89	ACSS1	NAGLU
ERCC3	PLA2G15	SPDYA	NFIL3	PYCRL	POU4F3	EMC1	KJ903919
STAG3L1	JHU14528	HG492309.1	HHLA2	ALKBH5	USP19	COPRS	TTC9B
CNTN4	NEDD4L	IL6R	DNAJC6	ATP6V0C	LOC100287792	YPEL4	OSBPL3
PRDM15	CHTF18	PTGES2	TMEM62	KJ901395	RMND1	LRFN3	PCSK1N
NUPR1	PCOLCE2	SAMHD1	ZNF140	GM2A	COBL	PDE8A	SLC7A8
BATF3	MCOLN3	FCHO1	RAG1	PWP1	TRAPPC12	RANGAP1	JUNB
KAT8	NUDT18	RNASET2	FAM63A	TAF5L	FAM170A	STARD4	SIRT4
WDR11	TLR4	COLEC10	SPATA6	SCGB1D4	FAM150B	RXRG	KRAS
MLH1	ZNF639	NT5C	ERVK13-1	ZDHHC20	KLF11	ESRP1	GNPTG
T	PCDHGA2	LRRTM3	ZNF684	FAM45A	NTN5	PLA2R1	KJ900909
AP3S1	TAF1C	LIN37	FAM11A	ZSWIM1	LIMS3	TNNI3	LOC401040
SLC25A47	CKAP4	ZPBP2	ENPP1	TRMT10C	MANEAL	PASD1	ECI1
MAP2K2	TREM2	RNF14	TMEM123	ZWILCH	ZBTB18	MTCP1	ARGFX
FAM122B	CFHR5	BT006925	OR10X1	PI4K2A	WDR70	PTGES	PAX3
PPM1L	AP3B1	SRSF5	PRKCD	LITAF	KIAA1958	BTG2	SPI1
HIST1H2AC	METTL14	SORBS2	FEZF2	ETNK2	NR5A2	IL18R1	ARHGEF3

RTN4IP1	C6orf226	NTM-AS1	C10orf55	KANSL1L	TSPAN10	PPP2R4	AK290580
C6orf223	FAM207A	MRI1	COBLL1	PPIC	ZDHHC23	POTEE	KJ904266
PLOD1	BCOR	STK38L	NR0B1	LOC399900	A1CF	C9orf91	CREBL2
MGAT4A	GOLT1A	BORA	GRM2	ADRA2A	ANKRD9	PDCD6IP	ARHGEF39
ALKBH2	ESRRG	TRAF3	CHRM1	PTP4A3	GATC	TUBGCP2	CTDSPL
GNA11	EFHD1	KJ902139	MAPRE1	FAM9B	KIAA0586	MFSD10	ZNF573
VWA9	IDH1	YTHDC1	HES2	LYN	CLPB	ATP11AUN	AK131224.1
ATP2A1	NG_033837.1	OR1S2	TEN1	GPX4	KLHDC7B	SPINK14	ADA
COQ7	ETF1	CD69	CSTF2T	LEFTY2	VEGFB	FRMPD1	LARP4B
BRD4	LOR	SRA1	LILRB2	GAGE4	NG_006966.3	PIBF1	TSPYL6
Q6p3f6	PML	ZNF746	XPA	GKN1	DNAJB12	FRAT2	RSPO4
DEFB110	SPP2	OFCC1	OR51B2	PSMF1	LOC105378283	POTEB	KJ902480.1
RASAL3	GPRC5C	C20orf195	Zmynd8	Ift172	IGKV1OR2-108	F13B	COL6A1
WFDC13	UGT8	MASP1	KJ904328.1	IL7R	EEF1A1	EEF1A1	CWC22
CTRL	WNT8B	BPIFB6	CTDP1	AATK	TTC13	HIST1H2AD	NRDE2
CCDC102B	SPCS2	C11orf57	AC124312.1	KLHL41	PRICKLE3	GLIS1	C6orf201
KJ901253	PSMD11	NPIPA8	ITIH2	DGAT2L7P	FGF6	MIP	PDCD1LG2
CMA1	ZNF560	OR3A2	RIMBP3	SLC6A15	ANGPT4	IFNL3	CHRD
FAHD2B	KDM7A	CRB2	SNCAIP	PM20D1	MMEL1	P2RY4	NPFF
CIB3	LZTS1	MYNN	ZNF304	SLC35E3	CCDC144NL	PWWP2B	SRGAP1
FSTL3	DEFB136	CPZ	RASL11A	DKK2	TMPRSS15	KRTAP9-8	TMEM128
SLAMF6	APCS	Gsc2	CPSF1	NUAK2	LGI4	OTOL1	DUSP8
PKP2	GRM4	RPL3	SCAI	SLC25A35	TMEM91	RABEP2	AKR1C8P
DTNB	GSX2	RASSF7	POLDIP3	ALDH1B1	MED18	CACNG1	ODF3L1
IL15RA	PXDN	KRT72	PPFIA4	BAIAP2L1	PTK7	BPHL	PLD5
TTLL9	GPX3	RIN1	ZNF362	GFI1	HKR1	RAB17	FGF19
PRG3	UGT1A1	FAM132A	HIPK3	GRK1	SGPP1	ADAMTS13	C21orf62
H6PD	TLE1	HR	ZNF687	SEC14L3	KJ900930	CRYL1	SRRD
SUMF2	KCND3	SLC28A1	FAM111B	DNAI2	MGAT4EP	CRYGN	AMICA1
SGK494	C6orf182	BC047084.1	ZNF264	ECD	PHYHIPL	FAM9C	PIGL
NRTN	DEFB107A	PRAMEF22	CD274	LHPP	C16orf72	BC092500	BC104209
ITK	FERD3L	LRIG1	MAPKBP1	KIFC3	ACVR1	Kdm5d	ZBED9
Crocc	AVP	DAND5	TTBK1	PTPN23	PRKCE	TKT	COL5A2
DYRK3	SOSTDC1	KCNK16	ZNF521	NCOA2	MS4A1	C1QTNF9B	ACE
CARM1	FSD2	USP29	STON2	FOSB	USP17L22	NRN1L	ACVR2B
TESPA1	HNF1A	ZNF451	TRIM26	PURA	Cebpa	HOXA10	ERI3
RCAN2	FGB	RNASE3	ACPT	MPRIP	FAM149B1	ODF3L2	IL1F10
PPP4R3A	DUSP5	MUM1L1	FMO3	DAPP1	PRSS42	INS-IGF2	PRSS27
LYPD8	INPP5D	MUC4	FBXL13	DUPD1	PRR19	OR5D16	ZNF300
KLHL26	REL	LOC101929479	GSPT1	SHH	NGRN	SRD5A2	TNNT3
RFPL4B	ARNTL	SASS6	HS6ST1	ARHGAP28	CCDC37	CYP2B6	GATA4
ZNF114	RAB20	LOX	STX1A	IFNA7	PPTC7	EPHB6	PKN2
GPR37	SYN3	AK054893.1	AC008382.6	KJ901803	SNX11	DCUN1D3	PRDM10
Tref1	IGLON5	VWA1	COL10A1	GAGE12B	MPO	FAM188B	NAPB

CCNL2	BRD7	LLGL2	SLC16A14	ZDHHC17	TP73	Foxn1	CCDC115
PROL1	MMP11	ATPAF1	TAS2R31	TATDN3	PLPP4	SH3D19	JAM3
C16orf54	ACKR4	XG	LASS1	UTY	TRIM10	ARFGAP3	Snpc4
KDM5C	Sebox	ZNF488	SEMA3E	MTRR	OTOS	CILP2	RHOT1
ADCYAP1	CCDC38	VAPA	ZNF532	LRPPRC	Smarca2	LIPC	NTN1
NPTX1	DNTTIP2	KCTD19	PTPN3	UFL1	NAALAD2	OFD1	PCMTD2
PTGFR	UGT3A2	SELP	ALDH1L1	FOXL1	EGR3	BC108926	TXNL4A
ST6GALNAC4	ITPRIPL2	ADAMTS15	ADAMDEC1	MAP3K2	CERKL	TRIB1	TAS2R20
GAGE2A	LIN52	LAYN	PON1	CTSO	MIA2	PRLH	MAP4K3
C4orf32	BMP8A	CD248	SLC4A1	BC067760.1	SARNP	KRT71	TDRKH
ZNF221	DAP	CYP8B1	PGAM5	PPFIA3	TGFB3	NGF	C8orf74
RAP1GAP	CLSTN2	HOXB1	NUDCD1	BC063682.1	ZNF761	CA13	Q6pcx8
ZNF432	ASB11	SMS	FGG	WNT10A	DMP1	ANKRD19P	MRGPRE
OR52E2	ADAMTSL1	RHBDL3	LRRK1	BC073767	GRM8	SELPLG	NSF
Smarca4	ADIPOQ	UGT1A7	GPHA2	WFDC10B	KHSRP	DAPK3	ZPLD1
OLIG2	SLC15A1	ZNF230	PABPC4	C16orf92	SLC35E4	MICU3	KRTAP10-5
PSPN	AMELY	RIPK1	PTPRU	PIK3R2	NCAM1	LINC01600	MAN2B2
SPCS2	Prdm16	NEUROG3	LPAR4	CASZ1	PRSS38	NYX	DNAJC14
IMPG1	AMPD3	ADAM5	SLCO1A2	ZNF543	PPP4R1L	SLC6A8	LOC641367
LPCAT4	HNRNPDL	Q14591-2	Q8r0e0	DEFB125	PRSS57	JMJD8	TGFBR1
MST1R	MUSK	AIFM1	OPRM1	VIPR1	FANCC	Znf516	ZNF423
ZBTB40	CCDC184	CSN2	CAPN13	MTMR7	PDZD4	USP20	CYP4F22
SLC26A5	BC089412.1	BC089413	MDFI	NCOA3	GLI3	IFT27	KIAA1324L
AMER3	FBXL17	CHRNA4	RAET1G	SNRK	FCRL5	MPP2	TEX33
Hoxa2	RBMXL2	LHX5	CRMP1	PROM2	KDELC2	SLIT3	DAPK1
BRDT	PGK2	PCDH17	SCGB2B2	KLRF1	BC073937.1	ZNF583	Ebf2
MCRS1	EIF3F	XM_009242791.1	SETD8	BOLA3	B3GNT8	SIGLECL1	CDHR4
BC071784	KJ906384	KIF7	CSF2	PNPLA1	ZNF385C	C19orf54	ZBTB16
ZNF132	ANGEL1	ADM2	SHBG	ERAP1	TK2	PAPD4	HEATR5A
GPR50	SCNN1D	BCL2L12	SLC11A2	RHBDD2	AACS	NUP85	ACO1
CNOT4	ZBTB11	POLE3	CRISP2	OR6C68	FMO5	PROK2	PHOSPHO1
COL15A1	LOC441178	SRCRM	SPATS1	PARP8	IGHA2	BC045761.1	HSF2
Q9erl0	CPEB2	NKAIN4	EIF4EBP1	ERV3-1	MUC3B	UGGT1	PRY
BRWD1-AS2	LNP1	NUMA1	MORF4	USP21	SCYL1	ARMC12	ANXA6
MCOLN1	MED15	ZBTB4	Tcerg1	KRTAP10-1	VWCE	PRSS48	PCSK1
AY358192.1	PPP1R3F	BCAS4	LINC00310	KIR3DS1	RALY	C8orf82	SMG1L
IL9	TBC1D3	OR52B6	C2orf66	NACC1	Mbd4	YWHAQ	TIMP2
AREL1	COLGALT2	CENPW	UGT1A8	CCL25	IFNL1	SLC13A2	RPL13AP3
FOLH1	RHOT2	RBP1	ZNF445	SMARCA1	SUPT5H	BTN3A3	AKAP10
CDCP2	HGFAC	SEL1L2	ZNF285	IGFL3	AMHR2	VPS53	CD300E
FBXW12	EPB42	TRGC1	KIAA0895	CHST3	LCE3A	EIF2B1	LASS6
ZNF562	PCGF2	FOXC2	EVI2B	EGLN1	KJ902354	TMEM8C	TGFBR3
RSPO1	PHKG1	NEK1	G6PC2	PALM2	ZNF180	HAL	SLC11A1
BC093717	AK126356.1	KLK15	FOXH1	AICDA	JAK2	HEPACAM	SLC22A31

AKT2	MAST3	MYLK3	SS18	BC089418	MLEC	ZNF112	Bicc1
NR3C2	GCNT7	C1QL2	TRAF3IP3	RNF19B	ANKRD20A5P	CASC1	STAG3
C11orf85	IL36A	B4GALT6	Q3zb72	ZNF703	CSHL1	NDUFAF5	SNW1
ROPN1	CHIT1	THRB	RGL4	D2HGDH	MSS51	ALDH1A3	ZNF202
Q6nv61	Eomes	PLK1	LPO	HAPLN4	SIK3	P4HA3	LTBP4
NME4	HCRTR1	NOS3	RPS6KA3	ENAH	SLC39A9	LETM1	ST18
KDM4A	SMARCC1	C9orf170	MCTP2	ENTPD8	PSG7	SARM1	SLC6A12
B4GALNT2	RBM47	ZNF254	SYTL3	VNN1	C12orf42	NR2C2	IQUB
SRPK3	FRG1	HOXB2	ZBTB47	UBE2D1	EID1	POP4	GLUD1
SFRP1	TLK2	GRK4	INSRR	GGTA1P	NOX1	AHI1	BC067366.1
SACM1L	GPD1	ZNF184	CLOCK	BPIFB4	CTSW	AMH	MGAT4D
ASZ1	ANKRD30B	MAU2	LOC387693	LINC00610	Repin1	DAZ2	OVCH1
DEFB105A	EFNA4	CSTL1	PPIAL4C	ACOT6	ACSF3	ZNF852	SLC39A12
DMRT1	CSF2RB	Zar1	BNC1	RGS4	FIS1	EIF1AD	DEFB104A
EMILIN3	EPHA6	PPFIA2	ROR1	SSX2IP	PSD3	GLRA1	VGLL3
SUDS3	RBMY2FP	NCAPH	FAM96A	RBM10	TLR5	SALL4	EPS15
MATN4	VWA2	LRRTM2	OASL	CCDC96	GAS8-AS1	LGALSL	B3GNT6
PCDHB2	SLC25A30	KLC3	ZNF524	IL17C	COX19	MNT	NPAS1
HRASLS5	USP4	KJ900991	CLDN25	SHISA3	AC213203.1	CRYGB	RAB41
KJ902330	LRIT3	ETNK1	CYP1A2	GGT5	CDNF	TMEM130	SAMD4B
Q6pfe1	TRIM32	CHAD	UGT1A5	DGKK	AEBP1	COL18A1	PBXIP1
ZNF155	SPATA7	DACT2	KJ906334	ZNF217	ZBTB21	GLI1	PCMT1
GLRX	LCP2	PRB4	FAM20C	CHAT	BCAS1	FASTKD3	BC044592.1
ZBED5	TTC6	LINC00303	INPP5A	SLITRK1	DSTYK	SDE2	PAG1
GRPR	SCN3B	SPATA19	BRCC3	CETN3	COL1A2	CTGF	PPP1R13B
ALPI	GRK7	C3orf38	CCR7	SLC36A3	ATP5L2	XM_003954378.2	RHBDL2
SAFB	BC128071	CP	LIPI	REN	ITPKA	JAK3	FAM188B
WHAMMP3	PPP2R5E	SLC2A11	LGALS9C	SGMS2	RAI2	CCT4	PRM1
WDR60	QSOX1	FGF8	GALNT2	HSPB3	RNASE12	GALNTL6	LOC151121
NOMO2	TSFM	GPR83	RECQL4	A0a0r4j020	EYA1	IL3RA	SSX4
SLC25A24	ELOVL5	CDK10	TOR2A	ITIH3	PTCRA	CHMP1A	ARL16
SMCO3	TRAPPC11	TAAR5	CCR4	KJ904402	NUP58	KJ900895	ZBTB20
NUFIP2	MLXIPL	ENPP3	UGT2B15	SOST	PPP1R12C	FBLN2	ERBB2
AGBL2	CYP2A6	CCR10	ZNF876P	ZNF462	Nfkbie	SOX21	BAD
DNAL4	TPM4	RAB15	WNT1	CDHR3	STK35	DGKQ	PYGL
KRT8P41	ANO6	KJ904353.1	VWA3B	PIWIL2	Wiz	HLTF	XKR6
PRR4	ARSE	FPGS	CDY2A	MB21D1	ZNF619	MIPOL1	KDM1A
ACVR2B	RYDEN	FOXD4	EFNB3	POT1	CFLAR	ECM2	ADAMTS14
FGF22	NEK5	PDP1	PTPN14	NG_023218.1	C14orf177	NPR2	CEACAM19
CPXM1	ZC3H14	J3js23	BC070062.1	UQCRB	LGI3	CFC1B	SLC22A1
BCO1	LRRC31	SCYL2	NAT8B	ADH7	TMEM222	TLR3	HIC2
KJ901014	ZNF509	GNPDA1	RBL2	PXK	HEPHL1	PGLYRP2	LGALS9
GCKR	KLKB1	FAM214A	ZIK1	ZP2	VCX3A	RNF150	MAGEB10
PARD3	TMEM88	FBP2	SIRT2	CALR	WNT9A	TEPP	SLK

LOC442028	SLC35D2	GRIK2	ACHE	ABCA2	PIAS4	IRX4	NEUROD4
ANGPT2	JHU19214	ARSI	C10orf25	GML	ODC1	TMEM8B	CHST13
MIDN	ZNF317	PHGDH	Zic1	Hes5	MEX3C	Fiz1	LIPN
BPIFA1	MXRA8	VOPP1	CRISP3	TNN	REEP5	UIMC1	KJ902965
FOXA1	LOC541472	AP5M1	DLX2	PRMT3	TRIP4	U2SURP	MRTO4
HRC	YS002	MPEG1	CNTNAP3	LTBP3	HORMAD1	TMEM41B	ALG12
METTL21C	HSD11B2	SMAD9	NDRG3	SRPX2	Nr1h4	ZNF345	ZNF497
TMEM104	CABS1	MAP3K13	VWA8	BOLA2	DMRTA2	HIST1H4G	LELP1
PQLC2L	BC036580.1	PRKY	SPDYE2	WDR90	MAP1S	DNAJB9	Lhx9
Q6axh7	Nc2b	ZFP30	ZFP91	ZNF440	LCN8	IBSP	CD163
NRG3	SFRP5	WNT3A	DDX24	ZRSR1	Q6p7w9	CSAD	TMCO1
ARMCX6	AK126796.1	RBP3	PRSS22	LCE3C	DEFB134	H2AFB2	APOA4
PRB3	LINGO1	MYLPF	Bcl11b	OR11H6	PAPPA2	GABRR1	TBL1Y
BC101016	CNGA3	KDM2A	BBS1	NEK9	HOPX	TMEM52B	CEBPD
Hoxc12	ZMAT3	RGS7	G3XAM8	TAT	SUV39H1	TAF13	ECSCR
ROBO4	MAP4K4	PPP1R14D	DNAJA4	KIAA1841	LHFPL1	GSG2	KCNE2
WDFY1	A2aik5	NKX3-2	GSTM1	LAMTOR3	APOM	TBX10	MEGF6
PRPS1L1	PSKH2	KRT83	ST8SIA2	BC094747.1	UBE2K	SPATS2L	ZNF267
RBM28	ZNF107	KRTAP19-2	BC041851.1	CTRB2	ODF1	COLGALT1	TMPRSS11E
KRT40	MEIS1	PLEKHN1	DIO1	BSPRY	NTHL1	FBXO16	Cbx2
EIF2B5	DEFB123	MMP21	C1orf53	RAET1E	NBPF5P	UNC45B	PRKAR2B
SLC51A	CASP6	ISG20L2	NOP56	Rax	Irx5	NFIX	KLHL42
LCN9	WNT11	COL8A1	APOO	PRDM12	MELK	AURKC	PDCD1
KLRC2	CYP11B1	TMEM164	TP53RK	CNOT3	STAT2	TUT1	USMG5
ADGRF1	PTX3	SPON1	RFTN2	NEU4	SLC17A8	BC037837.1	SV2C
TSGA10IP	ARHGAP36	ATF7IP2	PRTN3	DNASE1L1	NKX2-2	Cdx4	EN2
ZMYND12	EIF2S3	RNF113B	PPEF2	PTPRD	ZNF571	SLC36A2	NDUFAF5
ZCWPW2	KJ906410	DLGAP1	HORMAD2	PHOSPHO2	Sim1	Glis2	TMEM126A
WDR45B	LY6G6D	FGF17	BMP2	SGK223	CDC42BPG	ZNF334	MTL5
FAM167A	C7orf34	CC2D2B	EPHA4	FICD	RCOR1	NAB1	DUS2
TCL1A	MAGT1	CDK19	UGT1A4	DEFB130	SPINK5	LAMTOR4	KJ901180
TRAPPC6B	FOXD4L6	SLC25A52	SLC6A5	ARFIP1	FAM71F2	SASH3	CDX2
Dmrt2	Tlx1	NELFCD	MYOZ2	CIART	NODAL	CPO	CD300LD
CDKL4	PPP1R11	CDC42BPA	KIAA1161	CYP2D6	ERGIC2	ZNF711	SF3A1
ZNF383	ZNF585B	B4GALT1	IHH	CNBD1	GLDN	SLC28A2	MOGAT2
GALNT16	LRRC25	ANKH	GTPBP6	GCM2	RNPC3	OR5AK3P	CYSLTR2
C1QTNF8	DEFB124	LOC105372715	TMEM218	LALBA	TXNRD3NB	PLEKHM2	CNGA4
DENND5A	RNF212	CEP250	Lhx5	Sox11	Lhx1	TCFL5	FOXO4
FBLN7	MMP16	LIFR	TNS2	MAP4K1	C1QTNF4	PKP1	OPCML
MGAT3	ARHGAP12	THOP1	GK2	HLA-J	ZP1	LRMP	KJ900647
SLC2A10	BC032842.1	TMED5	VSTM1	KCNT2	COL8A2	LRP8	SUCNR1
OXGR1	TFB2M	NKX6-2	Lbx1	DNAJC17	GTF2A1L	ACRV1	SERPINA7
CD24	PTH2	LCN10	LCN6	DGKD	PDPR	INPP4B	TYW3
CRY2	OSCP1	IL1RAP	CDK5R2	ELSPBP1	Fezf1	Zfp811	ZNF143

IQCF6	ELANE	DEFB135	TIE1	SUGCT	SOAT2	DDX53	SMR3B
GOLGA7	PTPRN	TRIM68	Q922y8	PLAG1	VSIG1	KDM6A	RINL
ARHGAP6	LCE4A	RETNLB	NSUN5P1	FLYWCH1	FAM71E1	ZNF584	ZNF419
PAX7	ZNF454	RERGL	DEFB131	FAM127C	SCIMP	FAM216B	SEMG2
TAS2R10	GPR160	LDB1	Ebf3	Vsx1	Pdc	MIEF1	ZSCAN22
RBM24	PRAMEF3	LY6G6F	RNASE9	NCAN	CDKL2	MEX3B	ATP8B5P
ZNF624	CXorf57	CCDC153	PPP1R36	Tal1	E2f5	CEACAM1	SEPT5
TMEM255B	OBP2B	SERPINA11	JAK1	SBK1	MARK1	AOC2	KIF19
OCRL	ACER2	TFDP1	MTF1	TSC22D2	PRDM4	SDCBP2	SLC13A1
CCNT1	GUF1	HAND2	DRGX	EHMT2	DEFB106A	TTLL10	SAMD7
PNLIPRP3	ZNF2	AGGF1	PGK1	UBAP2	AMPD2	PABPN1	MSRB2
ADAM23	DGKH	LIMK1	AC113415.3	FETUB	FOXP1	MRPL2	DPH7
ZNF366	DMTF1	Sp3	TLX2	C4orf46	KJ905672	FAM20A	ISM1
ENTPD5	CCDC114	TOX3	KJ904164	DLG3	POLRMT	ZFP28	LZTR1
PIWIL1	SRRM1	PRSS36	CBLL1	ZNF517	CCDC173	BC031277.1	ALDH1L2
CCDC66	PIPSL	HMGB3	BARX2	PDX1	HOXC11	SRSF12	PABPC3
PIGR	TRIM33	AKAP8L	EIF2AK3	SPNS2	ALOXE3	TTC31	INCENP
SHTN1	LYPD6B	CREB3L3	DMRT3	ZSCAN18	GRHPR	ZFYVE16	MCU
LAMC2	ZP4	NTN4	NUDT14	PTPRK	NCF1	RMI2	THAP3
CFB	SYNJ2BP	Dbx1	Foxb1	Esrrb	Pdcd11	Q5xjv5	SPACA7
RNASE13	CUTA	PZP	MAP3K10	COL28A1	PON3	PDXDC2P	CRYBB3
TTN	LRRC15	KCNJ11	BC037829	DHRS11	DR1	BCAS3	JHU19600
Pou4f2	Bhlhe22	Prdm9	FOXF1	RGS17	RNF43	PDE11A	KRBA2
CST11	PIK3C2B	SCUBE1	AB937783.1	TAS2R5	HG492055.1	PPM1J	SLC31A1
E2f1	ZNF491	U2AF2	XRCC4	TMEM170A	ITIH4	ALG1L	SLC24A4
CCL1	SLC25A11	Foxg1	Six3	ZNF248	EMX1	Q4vad4	RS1
VSIG8	HIST1H2AH	THBS3	PPP1R9B	PEAR1	MRPS6	H1FX-AS1	RBM15
SMAD7	ZNF101	TBX19	UGT2B4	XAGE2	ARHGEF28	THBS2	SRMS
HPSE	LRRTM1	FMO1	USP38	NR1I3	Crnl1	SFPQ	SUPT20H
IAPP	ASTL	GGT2	TRHR	NPSR1	C9orf173	BTN1A1	KJ902055
DAPL1	DIS3L2	CCL4	TSPAN31	RSPO2	Tal2	Gtf3a	Tcf15
C10orf35	PSIP1	PLA2G2C	TMEM190	GBX2	RNASEL	PPP1R1A	TEC
KLHDC9	EFCAB6	KNCN	RPS27A	C20orf152	OR9G4	ELOVL1	LMCD1
HAND1	NKX3-1	Hoxc9	GDE1	DNASE1L3	TMBIM4	IKBKE	CDC42SE2
CSF1R	TNNI3K	RRM1	NAT8	TCF3	SIM2	KJ898030	PYY2
SERPINE3	MDFIC	TNR	SLC10A2	ITLN2	ST8SIA1	BC036697.1	LACTB
MOK	SHCBP1L	SLC23A3	RARA	NR1H2	ZIM3	CNNM3	FAM124B
CLCN7	MMRN1	GDF6	DST	MT4	GFI1B	FMN1	FAM19A5
GSTA2	BC060208	Bsh	JHU19590	PPRC1	CHD4	Tf3c1	CKM
CPXM2	OXT	CLC	PRB1	CECR5	LAMB3	NG_023220.1	MYRIP
MMP14	GMPPB	BACH1	SAMD8	KDM4D	GP2	ANOS1	B4GALT5
BMP15	KLHL35	BTBD9	SERINC5	SLC35F1	NG_015859.1	NPHP1	KIRREL2
NHLH2	PRDM7	TOMM34	STX8	FKBP9	C8B	F10	RYK
DGKB	ESYT1	GPC1	LYPD2	ALPP	TMEM38A	Pou3f1	Pou4f1

Q6ir13	SNAI3	Nr2f6	SFTPA1	CYP11B2	SUMF1	PRRT4	VSIG10L
MEIOC	GSDMC	CLK1	C21orf67	GPX5	PHC1	OR5R1	GOLPH3
SMAD9	Hnrnph1	FEM1B	RLN3	IZUMO4	CCDC85A	CXorf40A	BC060851
B3GALNT1	IDE	KIT	MORC3	BC012881.1	Prdm9	GTF2F2	MESP1
TRIM47	POLE2	PRSS16	TNFRSF6B	PIK3CG	DUSP2	CDK11B	SLC6A7
IFNA14	MLN	ARMC4	STK38	STRBP	ZNF615	GNE	YQ050
TNFRSF25	OIT3	OPN5	PRSS45	KLHL32	DYNC1H1	VMO1	FRS2
CDR2L	ITGB7	IRGC	FGF20	DEFB114	SCUBE3	CTAGE5	COG5
OAZ1	BC070352.1	MBNL2	TMEM23	CYP3A43	BC067086.1	BAK1	Foxc1
Supt6h	ZNF638	Snrnp200	C2CD4B	CPN2	MINPP1	GLIS3	PAPLN
GBP5	KCNS1	CDON	SCML2	TBR1	HSPB9	CSNK2B	ZNF507
ECHDC3	CASD1	PXYLP1	LRRC19	HHAT	GGA3	HSP90AB1	PAFAH1B3
ERGIC3	LMO3	XM_006509802.2	ACTA2	VWC2L	INPP5J	ROCK2	BRAF
EHHADH	KIF19	GPR22	HMGA1	ISCA1	Prox1	Rb1	A0jns7
Q504n8	MATN3	PGA4	PIK3C2G	EFCAB13	TUBAL3	PCDHB7	CELA1
PPP2R2D	GBP4	GCM1	TRIM49	SEC61G	COL26A1	KRTAP16-1	NDUFAF4
HMGN4	LOC338797	RPL27A	TRBC1	CORO1A	Onecut1	ETV1	ABRACL
SLTM	WFIKKN1	UBE3B	NKAPL	SUDS3	ZNF343	Insm1	Q6nsr1
ZNF646	Capn15	FSTL1	C1RL	DNASE1L2	ERBB4	MEPE	CNTNAP3B
BC031259.1	NANOS2	PHACTR4	SOX15	POU2AF1	Cdx1	GINS1	APOF
OVGP1	WNT10B	GCSH	PPP2CB	GUCY2F	ERN2	AP1G1	CCDC178
MMRN2	RMI1	ZNF800	Raver2	ZNF420	RHOQ	JHU19309	SH3RF3
CD1C	VN1R2	CCDC28A	YK006	RBM48	ZNF253	TIAL1	CREG1
C14orf166	ZNF146	HEYL	ATP1B3	FGFBP3	ADAMTS16	UBE2NL	PPP1R9A
PI4K2B	PRPH2	BC096236	DGKG	DNTT	RHAG	ZNF443	ZNF45
PTGIS	LAMB1	AMBN	PAX4	IDUA	TECTB	CCR2	PCDHA4
C18orf23	NRCAM	TATDN1	ABHD5	NAA60	ZNF225	ZNF219	NFE2L1
FAM117B	BC132880	NUTM2G	TOP1MT	NLRP5	NEMP1	FAM187B	SYT10
SUV420H1	BC045753.1	PACS2	CENPC	KJ903857	FABP3	KIAA0040	KJ901766.1
ZFAND6	EZH2	NOP14	FBXL5	LRPAP1	HSD17B1	ZNF843	CASK
CHUK	ZNF660	ZNF214	GZF1	PAK7	PTPN9	GPR135	PLA2G2E
ASIC3	COL17A1	GPC4	FABP5	MYH13	RAF1	SYTL4	CAV1
PLN	MAL2	GTF2H4	ATP11C	TEKT5	YARS	APLP1	KJ904099
DZIP3	POLR3H	ZSCAN23	NLRP13	OTOP2	SMG6	MS4A14	NKX6-1
IRF2BP2	HLA-F	DEFB129	SURF6	SCYL3	STARD5	PRDM5	CNGA1
SRP14	CYTIP	PTH1R	CYLC1	THEMIS	KRT6B	PHKB	TCEB3CL
TRIM56	HIST1H1B	PLPPR4	SYNGAP1	CRLF2	NUP188	ARMS2	CTC-329H14.4
CCL16	SIAH2	PJA2	UTP6	PIAS1	CCDC25	RBP7	CCDC50
UPK1B	INSM2	WDHD1	API5	KERA	SLCO6A1	FSHB	HCN2
RAB36	FOXE1	FAM65A	TSC1	PTPN21	ST6GALNAC2	GPR158	ALPL
IFI27L1	AIDA	IPO8	OR1D4	SLC22A14	SYT14	ZBTB24	TMEM5
SIX4	TUB	KRTAP5-4	SLCO4C1	RBMY1E	NAT8B	ZNF154	DEFB116
CLCN3	IL36RN	GPR137	TMEM184A	SPOCK3	STK19	MRPS35	USP53
FAM84B	NADK2	FOXE3	NOC3L	FMO4	SLC13A4	TLK1	KCNQ1

GRM1	GLP1R	NOD2	CEP164	ZXDB	EME2	ITGA11	KCNJ9
ASB15	SORCS2	UBXN1	RNF139	SSPN	TXN2	CABP1	ADAMTSL4
MAP1A	C7orf43	C1orf168	KRT222	ZNF276	GALP	ESCO2	MLLT10
MTSS1L	SLC45A1	GJD4	TLR2	CEBPZ	DDX47	WFDC5	CIB2
PLEKHG4B	LAMA3	LRRC16A	CFAP97	PPAN	HOXC13	TADA2B	GYLTL1B
KJ901286	PTPN20	PPM1E	GIT1	OR6C2	GDF11	RAET1L	ANKRD28
KRI1	KIR2DS1	COQ10A	GLIPR1	TJP2	MPP6	KRTAP2-3	BLMH
ARSB	WNT16	PCDHA10	ALX4	CPNE7	FOXF2	ZNF705D	FBXW2
SGSM3	CUL9	C12orf63	FNIP2	FGF2	UNC5B	SPINK4	RFPL2
ADORA1	SIGLEC10	TTLL11	PPP4R1	TSHZ1	RNF123	ALYREF	THUMPD2
GTPBP1	B3GNT7	OR52A5	KRTAP6-2	RAPH1	FIGNL2	HCN3	INSC
SNX29	LIX1	FZD3	FAM160B2	C11orf42	ARPP21	CLDND1	PLXNB2
C1orf229	SDK2	POU5F1B	HOXB4	CYP2J2	HOXA3	CYP2S1	ADRA1D
SPIDR	CDC42BPB	SKOR1	CEP120	CCDC51	CLUH	HOXB13	GSTT1
PEAK1	ATR	ZFYVE26	DMRTA1	TBX18	HACE1	IL27RA	B3GNT4
ARHGAP11A	GMEB2	EPHB2	KIR2DS5	SLC16A8	GPX6	IDI1	RNASE4
MB21D2	LYZL1	CAPN12	SNX29P2	CLRN3	METTL18	YIPF6	YIPF2
PALB2	CPLX4	ANKRD65	SLC5A1	STRA8	ADGRF4	FLNB	PRR25
PLXNA3	SLC18A1	CCNJ	CHERP	CX3CL1	DDX3Y	HS3ST6	HUNK
PTCHD2	LCA5L	FAM81B	GATA6	ZNF438	SLC38A5	SLC26A3	DNAJC25
PHKA1	ANKK1	PLXNC1	KRTAP5-2	MARK4	FGFR3	PIK3R6	INSL5
ZP3	EPHX3	SATB1	DDX5	ASAH2B	DHRS7	NT5DC3	A1BG
TBC1D3B	KJ903784	TMEM132B	DHX35	PAOX	CD1A	CYP51A1	ZNF561
ZXDA	HIST1H1T	ATAD5	HMX1	HELT	RLN2	UMPS	ABCD3
COQ3	MOS	USP50	ESYT2	CCDC85B	MTFR2	ZNF337	EVX2
HSF5	RAD54L2	NGB	CERS3	SLC12A3	OPRD1	ADAMTS17	FANCD2
LMF1	FLT4	PNPLA7	SPDYE1	KIAA1522	STRIP2	DGAT2	GRB7
MMP10	FMNL2	FRMD4B	ZNF311	PROX2	CAMTA1	VILL	CDH15
ATXN10	RAB6C	GRXCR1	GLT6D1	STK39	HERC2P3	WDR17	IGSF11
CLEC11A	BMP1	CHST9	ORMDL3	INSIG2	UGT2B10	MEGF11	AGO2
ALK	SV2A	IMMT	DEFB112	BCL9L	FOLH1B	NOS1	CHRNE
COL4A3	DNAJB11	TNRC6C	MYL2	ARL13B	ANKRD12	DACT1	ZNF648
CLU	KJ903720	FZD9	LRIT1	POLR3G	WFDC12	OPN1LW	TRO
MAGEL2	ZNF185	RALGAPA1	PRMT1	SEPN1	AASDHPPT	MFAP2	TM9SF1
OR51E2	SVIL	SCARF2	ARHGEF9	NLRP12	VEGFC	SDHAF4	CHST7
HIST1H1D	CLN5	GDPGP1	TAAR9	SLF2	DMRTB1	IGFL2	NAF1
KIR2DL1	KHDRBS2	WNT5A	VPS9D1	AQP4	FAM50B	WDR74	MIB2
ADGRF5	RRM2B	CLEC18A	CLCA1	SPANXA1	HIC1	RTTN	E2F3
DOPEY2	FRRS1	RPF2	OR6V1	TMPRSS11D	MANBA	TSACC	VPS36
GTPBP4	NRK	NBEA	ZMIZ2	KBTBD4	HOXD12	MSGN1	ZNF667
TGFBRAP1	SULT2A1	GUCY2C	NPTXR	PADI6	GALNT10	GHRHR	BACH2
RNF17	ENDOU	NPIPL1	RCN3	RBL1	SEC24D	OLFML1	SFTPA2
KLHL33	NXPE4	KJ904213	HDC	ZNF418	FBXO17	PDK1	DSC1
CD109	RNF215	KIR2DL5A	LCE1D	SLC22A10	SPACA5	CPEB3	MRPL9

KLHL4	MSH2	MYLK2	GBP6	HAT1	JUND	ZNF708	OXR1
CD34	THEG	CYP2C19	EN1	FZD8	KRT12	ANKRD20A3	LEMD3
HIVEP2	CIRBP	MAGOH	MAGEH1	IFT88	DNPEP	BRCA2	MBD3L2
ZNF80	SP8	TBX1	FLAD1	KIFAP3	UNC119B	CLLU1	LINC01565
KIF20B	KRTAP5-7	GJD3	PPAN-P2RY11	HIPK2	SCRT1	COA5	STYK1
SGF29	TMEM19	SDF4	C7orf55	CIITA	MYO7A	IL20RA	BCHE
LRCH4	BANK1	C6orf142	ZNF627	KCNK18	FAM38B	DCDC1	C1QL3
PRKD1	AGRP	ZNF668	OSGEP	METTL8	JOSD2	CORO6	TRMT10C
MGME1	SLC9A1	OGDHL	ZNF652	DDX23	LSM2	EEFSEC	PGAM4
ITGA2	FOXD4L1	CES4A	FAM208B	PLEKHA2	LGALS9B	CCDC6	INHBC
MC3R	PRSS53	ADAMTS19	NRXN3	AHRR	MASTL	KDR	IQCE
HMOX2	LIN54	DEFB109P1	DEFB113	ITIH6	PDE8B	SH3PXD2B	SKIDA1
HSPA12A	O75505	CLDN20	TRA2A	ENO1	HLA-DPA1	CACNA2D4	LRRC37A2
PKD1L2	ZNF181	ZNF837	FOXN4	SLPI	SNRPD2	CRTAP	PABPN1L
OR5AC2	POTED	NPNT	NRG2	TMEM213	DEFA1	PDZD8	ZNF595
CLPTM1L	TRAM1L1	KIAA1644	SFRP2	TSPEAR	PPP1R12A	PASK	ZNF572
NCOA7	AGAP3	ATP10A	FOLR3	OR13C9	DNAJB13	UGT2A1	HS3ST4
KRT3	MIIP	TUBB4A	RUNX2	CIAO1	SPO11	FAM192A	MYBPC1
HCN1	TMCC3	SLC24A3	BAI1	IGSF5	ANO10	TRIM71	PIGH
IRAK3	EVC	METTL20	TMED7	GNL3L	COL6A2	C17orf53	LAMA2
ABCA4	NAV1	PEX11G	DCAF12L1	WDR49	SOHLH1	TCF23	ZNF117
OSCAR	NEUROG1	EBPL	HELB	NMS	DCST2	PRODH2	FKBP15
SPINT4	LIPF	ADAMTSL2	CHRDL2	A2M	SPDL1	LGI2	ADAM28
IL6ST	NANOS1	FOXD2	NKX2-4	SCX	APOC3	MX2	SLC1A5
PGGT1B	MMP20	NKTR	VWA7	ITIH5	ADAM7	ZNF772	PRTFDC1
HDAC3	DPP9	NUDT7	HIGD1C	LY75	MTNR1A	SLC25A37	ATP1B4
DLL3	DPH1	GRP	AFMID	TXNDC15	C6orf15	ABCC12	POLN
KRT39	LNPEP	HPR	KHDC1L	HBS1L	EFCAB1	CANX	SPRED2
DBX2	TCF20	ACER3	TMEM198	BFSP2	FSCN2	GABRR2	NDUFAF6
ADORA2B	PTAFR	EXOC6	EFNA2	NBPF9	PRDM11	FCAMR	MCF2
FAM65C	PSORS1C2	FSTL4	VSIG2	NARS2	SPAG1	MYSM1	BRD1
NPBWR2	TRIM2	TCF24	ZNF675	ZFX	KAZALD1	MCM3	HPN
CTNND2	TMC2	ANO2	RANBP1	FAM129C	ZC3H7B	ZCCHC24	SLC37A4
RAP1A	CD200R1L	C4orf45	BROX	ASB10	PRKD3	AP1S2	KRT23
PDE1B	QRFP	FAM184A	LMLN	KRTAP17-1	KLF14	RHD	NPVF
PPP1R1B	KCNA3	AKAP8	PGAP1	KIAA1211	C22orf42	PTRHD1	LDOC1L
DMRT2	TPRX1	ZNF704	LY6H	ACOT2	TACSTD2	PPP1R3A	ADAMTS2
TRIM73	FABP9	CCDC18	ABCC8	REXO1L1P	SOCS7	HQ258637	SETD9
TMPRSS9	TRIB2	GNA15	NDE1	NRAP	CACNG8	CDRT1	SYCE2
MAFA	ZBTB41	ZIC2	KIAA1328	RHO	ARMC9	TDRD7	CCDC22
OR14A16	PRTG	MAP1LC3B2	TAS2R30	ADCY5	HES3	LDLR	SDHC
ALDH1A2	NCOR1	WWC1	LMAN1L	SLC9B1	C9orf106	HMX3	SLCO3A1
NPEPL1	XKR4	CDK12	KRTAP6-1	SOCS1	KIAA1024	FAM120AOS	ATP13A3
CALCB	DEFB128	ZSCAN26	OR4A4P	UTP23	PSAPL1	GPCPD1	DDX17

KCTD8	MAPK4	MLH3	SOX3	SCRT2	MRPL57	DGAT1	RENBP
TAOK1	KRTAP19-3	SSX6	EPHA8	ATXN7	PCSK9	ABCA10	ASIP
PLA2G2F	ATP6V1C2	PILRA	MRFAP1	FANK1	SCPEP1	SEPT3	DCUN1D4
SYNPO2	SORT1	MTERF1	OR51L1	KRT25	SLC6A2	GOLGA6A	KDM5B
RPL36A	PIH1D2	RETSAT	PTPRZ1	MLXIP	UGT2B7	SLC30A3	HCRT
HRK	DNMT3B	CHRNA10	GRIK1	GPR142	NBPF20	LLGL1	VAV3
TM4SF4	SPOP	YWHAE	ANP32C	TPSG1	POLQ	CXorf58	OR7C1
TBPL2	PPA1	PPP1R15A	TNFRSF12A	TXNDC2	PPP1R14B	GSTA5	SH3PXD2A
FIGLA	RDH16	LPIN2	GBX1	RFXAP	TSNARE1	CNTNAP5	PHF2
SERPINB11	CT45A10	ZNF367	ZNF404	SGOL2	TTL	TNPO1	JSRP1
MYEOV2	LCE1C	EHD3	ARID2	GBP7	SSMEM1	BOC	RREB1
HHIPL1	EXOC3L4	FAM98C	MBD2	ZNF770	CEACAM8	PARP1	CNPY4
HEPN1	LOC400555	GAGE10	NUGGC	SEC14L5	ITGBL1	SIK1	MRPS15
BCR/ABL	UBXN7	FBXW9	IQCF2	ZNF645	ARHGAP8	SLC16A3	NEU1
RASGRP1	TTC14	NEDD4	SEZ6	NEMF	TSHZ2	GDPD4	PIM1
ARMC8	AK5	FGF18	APOL5	KIF4B	EMID1	TEX9	DMRTB1
ZNF714	ZNF768	CDC27	RAB31	CD5	ADCY8	SPAG4	PER2
JHU17612	HOXD13	DUX1	ZSCAN10	SLC8A2	PLEKHB1	RNF31	LYPLA1
TCEB3C	PRM3	SLC16A9	ZNF33A	KTN1	CASP8AP2	ASCL4	SOX1
KARS	OR51B6	OR51A2	IL23R	IRX1	PTPRT	OSTN	FTMT
FLCN	GPR56	KRT18P55	FGF9	ABO	PNLDC1	ZBTB34	SEPT8
ZRANB3	HBB	TXLNA	INPP4A	EPGN	GPR149	FCGR1B	C10orf90
RPL21P44	SIGLEC12	RPL9	DNAJC27	COL22A1	PRDM2	NKX2-6	ONECUT3
ZNF121	NFX1	TCP1	BTBD2	DOC2B	BAZ2A	PARP4	ZBTB7C
DENND4B	C3P1	LHX3	AJAP1	GPR88	C2orf61	ALPK2	TWISTNB
UBXN2B	NOA1	BSDC1	MARCH4	LMNB2	OR5K1	TBC1D4	RXFP2
TM6SF2	B3GALT6	N4BP1	MZT1	PUS1	ATP6V1E1	DENND6A	TCP11L1
XPO7	FYB	RGS11	RDH5	ZNF682	CLEC4M	PSD4	KIR2DL5B
NKX6-3	NPHS1	MED12	OTOF	AGXT2	CD300LG	FGF3	LCE2B
FKBP4	PAM	NLRC5	ANKRD30A	EEA1	SLC6A20	LRCH1	ZNF774
RBSN	GPSM2	RPL32	NEDD1	OR4Q3	KRT82	SYT8	KRT75
KRT27	IGFL1	PRSS55	QTRTD1	SNAI2	AMIGO2	EYA4	SLC45A4
HECTD3	KJ901314	FNDC7	BPIFB3	SLC7A6	CHST10	RP11-451G4.2	ITGA4
RSF1	DPPA3	CALML6	NPIPB15	DEFB127	CYAT1	FAM163A	CENPK
MIOS	MOB2	SLC4A7	ESPN	PDE6C	BHLHA9	ZNF30	MESP2
TRIM7	MT1A	SYNGR3	CSPG5	CEMP1	KCNQ3	WHAMM	COL16A1
KCNH2	SLC22A16	PYGO1	DUSP12	TRIM21	PSME3	CCNB1	UBE2Q1
ZBTB38	NPHP3	FOXI3	UNCX	ZKSCAN2	LSAMP	AZIN2	INHA
ADAM11	VEZF1	ARID5B	CABP2	ATP10B	MAGI1	MMP25	GDF7
GSTT2B	NARS	IGF2R	RLTPR	SALL1	GNRHR	ZNF696	CD81
MSH6	CDH18	MED25	IFT122	NPAS3	CCDC125	KRT74	WTIP
STK32C	A0A087WT11	CNTROB	ALDH9A1	FBXO44	HLA-DRB3	LY96	NPTN
SLC30A10	IGF2BP1	LYSMD1	AGPAT3	KBTBD8	ALAS1	GLI2	HOXA13
ORC5	ISYNA1	OR52I1	TGM7	GAS2L2	STXBP5L	PCNX	MBL2

OAF	TTC5	ZNF701	WFDC8	ACE2	PSG8	TGS1	CD93
C1orf95	KLHL18	SSB	OR4N2	PAIP2B	XYLT1	GPR150	AKAP13
CBWD3	PLXNB3	RTP5	ARHGEF10L	MYF5	NR4A3	BHLHE41	DBH
IGSF9	POTEA	S100A7L2	USP7	KRT85	RNF157	TMEM155	NPS
DHRS7C	SPINK9	IZUMO1R	UGT2B11	FMNL3	SYT13	TXLNB	NUDT11
SF3A2	VCL	ARHGAP1	LCE5A	SIGLEC11	FBXO43	MLLT4	PAPD5
DCDC5	TFCP2L1	KDM3B	SCGB3A1	MEAF6	LRRC14B	DHX34	AQP12A
GRAMD2	FAM19A1	ZNF43	LANCL1	MOSPD2	SBF1	MOCS1	IRS4
SLC16A2	TRPM6	MRPL37	CYFIP2	NUCKS1	ALAS2	STX19	TBP
SPTBN2	TNPO3	NKPD1	PAGE2B	TRIM49C	HMX2	BARHL2	ENPP2
CDSN	ABHD2	ZNF70	POTEH	SPRR2E	SHISA6	HOXD11	TTLL13P
HIST1H2AB	IL12B	KJ902403	CCDC74B	PPM1H	HNRNPH2	PCDH20	TMEM67
GON4L	FOXQ1	SKIV2L	DCTN4	GJA1	KIAA1648	EPX	TLL2
ARHGEF12	TNFRSF11A	ATP4A	LMTK2	PIWIL3	SCRIB	NECAB3	C5orf22
CASP16P	PDE5A	FBN2	IDO2	KJ904326	TCTE3	OPN1SW	RNF149
EXOC4	EDAR	CHST2	QRICH2	CRIP3	FAM169B	CSMD1	OR10T2
RER1	PHF8	MED19	ZNF625	NSUN6	NSA2	SBDS	C14orf105
ELOVL7	ROS1	CASP9	GBP3	SLC27A5	ADGRA1	SLC22A25	EXOC6B
ZNF492	ST3GAL4	HEBP2	SIX6	PGLYRP3	CIT	KDM5A	SP5
NGEF	AP2A1	SLBP	HMGXB4	RABL2A	SYNGR1	IL2RA	EMR4P
PGR	MFHAS1	SRRM4	RSPH1	ZNF341	ZNF14	BCKDHA	STAT6
NELFE	PLXNA1	RBM5	LIM2	HSD17B13	TAS2R4	KALRN	HSD17B4
PKP3	ATP1A1	ISX	GRB14	EU832464	SULT1C3	CYP4F8	ETV2
NAA11	THAP9	ATP2A2	RASA2	FOXR1	FOXB2	PRG2	SLC22A4
APLNR	MKL1	SEZ6L	EFTUD1	CHP2	KIR2DL2	ATP11A	UNC5A
SSR3	ZNF529	TEX14	SCAMP5	KIF18A	PDE6G	HSPA1A	KJ900918
OTOP3	CNTNAP4	DDX31	HS6ST3	SHROOM3	TCF7L2	UQCRQ	CDKN2A
ZBTB3	WDR12	KRT10	ZNF106	LYST	TECTA	SEMA4B	WDR78
PHF20	ZNF580	ZNF546	MAGEA1	FCGRT	DEK	RFC1	CYP2A13
KLF16	SRP72	LCE3B	SPINK13	PATE1	POU2F3	AVEN	KIAA2022
PAXBP1	CASP14	KRT80	SCRN1	STIL	GYS2	LHX8	BNC2
SLC7A4	GNG3	MIPEP	RANBP17	SRL	SLC9A4	DEFB126	LCN15
FAM110B	NMD3	PLD6	HDX	BCAR1	AKAP12	RUFY2	OLAH
TESK1	RIPK4	TKTL1	POM121L9P	SEMA4F	PDE6B	ATP2A3	LCE1F
OR4D2	DUOX2	PTX4	GRIN3B	KCNMB4	NME6	SHOX2	EIF4G3
DNAJC5B	CELF5	ZMIZ1	NCKAP5	RIMS1	CYP1B1	TBC1D15	BSX
ZNF195	ZNF382	LILRB4	SOD3	RB1CC1	APC	ACR	CHRNA9
LIPK	KCNH8	CCDC73	HNRNPUL2	ZNRF3	TACC1	PRMT5	VWF
ARFGEF1	VHLL	YT011	LOC401296	LRRN1	GANC	FIGN	DKFZp434K191
NKAIN3	LHCGR	TRIM67	KCNC3	OTOR	NCK1	SGPL1	HERPUD2
KCNJ5	MSR1	ABI3	FAM49A	SLC30A8	COMP	HLA-G	DAZL
LPCAT1	SCAF11	KRTAP19-6	SLC8A1	HMGN5	HAUS4	ISOC1	PRIMA1
LRRC37B	MYBPC2	SOX18	MAF	ZNF804A	MINK1	PPY	CCDC102A
SP7	ZNF699	DPF3	GUCY2D	LMO7	CNTNAP1	NOX3	PTPRJ

EDDM3B	UPK3BL	SOX2	EPDR1	AZGP1	NOXRED1	TSPYL5	TLDC2
MRPL45	AGBL4	KSR1	ABCB6	PEA15	PTDSS2	EGR1	MARCKS
GEMIN4	CHD5	TMEM179	CTAG1A	TAS2R39	CCNO	TCTN3	SEMA3C
SLC22A7	PHF5A	BHLHE40	KCTD1	GPR68	LTB4R2	FOXK2	DUXA
NFATC2	GEM	DOK2	RTP1	YV008	MYH9	SSTR3	MN1
CDH10	PRAMEF12	ZNF479	ZNF197	PRR3	XCL2	MRVI1	OCLM
ARSD	POU3F3	SERPINA13P	SOS2	FAM9A	NFKB2	PTF1A	RRAD
STX6	FBXO5	OR8U8	ZSWIM7	ERCC4	KCTD2	NIPAL4	MMP26
SNRPA1	SERPINC1	GLMN	IFNL2	ZRANB1	SRA1	CCDC172	AMMECR1L
HSD17B14	UQCC1	DNM3	CYFIP1	TNFRSF1A	ADAM20	OR8G1	KRT78
IQGAP2	SAMD1	MAMDC4	GPR42	RIMS2	XIAP	SLFN11	CNTFR
SRPX	C4A	RP1	BOD1L1	FLJ21901	C17orf70	PBX3	KLF13
ZNF417	KCNK1	ME3	PPFIA1	F5	ULK1	RNF39	PANX3
PTCH2	BRS3	SFMBT2	KRT9	SKA1	KRT32	TNF	ATP2B3
FAM58BP	GADD45B	KJ903835	TRIOBP	SUN2	ANK3	AWAT1	SEPT14
TAF2	PPARGC1A	ITGA10	TAC4	ACTN4	NEFM	ACADSB	FAM45BP
C2orf83	CTAG2	LENG8	G0S2	GJA8	EVI5	SLCO1B3	AIM1
IQSEC2	MYO5A	DOCK3	CUL2	APPL1	OLFM3	CLK4	HUS1
HSPD1	PELO	KRTAP10-10	DCLK2	CLCA4	PLCB1	ALX3	SP1
SP6	MCL1	SH3BP1	ILDR2	CEP85L	ADGRF3	GSX1	OC90
NPW	INSL3	OPTC	EDA	SFTPD	WDR41	CCDC158	AANAT
COX7B2	FAM217A	KHDC3L	FAM166A	MYBPHL	PCDHB5	OGDH	TXNL1
CDH2	HNF4G	ANKRD34B	EIF2AK4	MATN1	LEPR	AS3MT	EXOC1
TTC29	MET	USP6NL	DESI2	ZNF395	RBM45	ESPL1	CACTIN
PSTK	ZNF575	PRDM13	TCF7L1	POF1B	PDE1C	KCNJ4	RFPL1S
AP1B1	FAM163B	C11orf40	DERA	PDIA2	BTBD7	CEP126	P3H1
NPAS2	AFAP1	PMPCA	SKI	AFF3	ZFP41	TNFRSF10A	CLCN1
PRKG2	PKD2	TAS1R3	ZNF17	PCDHGA3	ABCA9	NOM1	ZNF649
NES	CEACAM4	GALR3	YK033	C9orf139	FAM135A	SMC6	FNBP4
SEC62	KRTAP10-2	ANO5	KCTD10	ALS2CR12	KRT86	ARHGAP31	HQ258564
PRDM8	MYT1L	WRNIP1	DRD5	DRD2	LCE1E	HAUS5	CD1E
TOMM40L	PARP3	CRLF1	TXNDC11	CBLN1	ZNF365	COA1	SFXN4
SAP130	RSPH6A	BTG1	SAMM50	ESF1	HOXB8	ANGPTL8	SYN1
ATG2B	CEACAM16	UNC5D	TRPM1	EPB41L4B	ADGRL4	CHAF1B	LYZL4
CCDC8	MYH3	FRMPD4	MIXL1	PHOX2B	AKNA	FOXO1	FKRP
KJ902006	BAAT	TNKS2	DEC1	ARHGEF4	MNX1	FOXJ3	METTL12
NSUN4	RPS6KA4	ICT1	TSPAN33	CEP19	PISD	TTC25	LBH
NAP1L4	TEX11	B3GAT1	CLIP2	POU3F4	SLC6A9	OR51V1	LRRC4B
GRIN1	SHQ1	DNAI2	ATF1	CXorf56	ADRM1	UGT1A6	FBL
ADAMTS13	ABCA7	TSPYL2	RACGAP1	ARHGEF2	PUS3	PLAA	OPLAH
FER	B4GALNT3	ATP2C2	HES7	APBA1	IFITM5	MYH4	BCAT2
PMM2	SGTB	ME1	AKR1B10	POLE	USP9X	ZNF780A	VEPH1
AP4E1	BRIP1	KLF2	BHLHA15	YY2	ZPBP	GPR146	ADNP2
SOX4	YJEFN3	PCDHB6	TSPY2	KRTAP4-5	CTSL3P	OVCH2	SIRPB2

REG1B	OR4C5	SOAT1	SNIP1	CRNKL1	TRERF1	TM4SF5	MEDAG
AQP11	ATP5SL	EGR4	ZNF425	ZFP92	CCP110	COL1A1	MIOX
SEC14L4	LINC00896	SLC2A7	MDGA1	NTSR1	XDH	UGT1A3	PHF3
GNRH1	DHX38	UBE2J1	CORO7	ADCY3	PIK3C2A	COA4	L3MBTL2
C16orf71	EU831996	WDR72	NALP5	IGFBPL1	USP32	DUB3	SLC44A5
MVB12B	CEP290	POTEG	NAV3	DEFB118	RIPPLY2	C5orf56	CPNE8
FBXL22	DENND2C	MICALL2	CAMTA2	KRT28	ZNF713	PARD3B	P3H3
ABCA12	SERGEF	SCGB1D2	NAAA	RPP38	C3orf58	FASTKD2	ZNF823
REST	TGIF2LY	TNK1	LSS	PAPOLB	LINC00634	LL22NC03-5H6.6	MOV10L1
GJC3	DEFB115	DCC	UTF1	TGM6	ATP8B4	ZGLP1	TELO2
IL10	C5orf60	STH	KIAA1524	BAIAP3	MSL1	RAB3A	DPEP1
PREX2	SLC5A4	NPIPA1	TREX2	SOGA3	MGAT5	VPS33A	TMTC1
GABBR2	ELFN2	ZBED6CL	DOCK10	NOTO	ZNF320	YEATS2	PIWIL4
CCDC117	CYS1	RPL2B	IGDCC4	NCR2	CEP162	GUCA1B	H2AFJ
COL19A1	RUNX1	RBAK	NANOG	BAFF	TIPARP	TRPV1	PIDD1
KCNF1	UBQLN3	AMPD1	ADAM19	CADM4	PDCD7	OCM2	OR7E24
AQP6	ADGRG4	VPS37D	FKBP11	KRTAP4-12	ALOX15	PCGF6	NT5C2
SCN4A	CLPSL2	LAMB2	CFAP57	C1orf127	CCDC142	HOXB3	KLF17
HOOK2	FCRL3	CAGE1	CD3EAP	ZNF354B	UHRF1	OR13C4	LCTL
PGA3	GPR27	TRPV6	NPPC	VPREB1	ICOSLG	ZGPAT	GPRIN3
DDX27	PCDHGB4	SOX14	ZNF234	ENTPD2	DND1	RGS21	SPAG17
DUX4L2	LHB	CR1L	INSL6	KCT2	ITGA1	GCNT3	TNFRSF10D
REXO4	ANKRD20A1	ADAM30	EGFL6	MOGAT1	CNGB1	KRTAP5-1	ZNF284
STOX1	BTBD17	GRIK5	POLR3B	MAEL	SNX4	CCDC47	HPS3
NPY1R	TET1	TAF3	REV3L	CD20	ETV3L	PRDM6	GNA13
JPH3	PACSIN3	C22orf23	TMEM191A	GP5	TPGS1	DACH1	GLS2
PCDHA1	MYZAP	CINP	FBLIM1	ORC2	ARHGEF28	PYGM	XPO4
SPHKAP	PRR27	F8A1	ABRA	TMEM59L	RBM15B	KBTBD3	TAS1R2
SPANXN2	ZNF674	MEGF9	RGPD5	CFHR4	C6orf10	PA2G4	MRPL54
PRR11	SLF1	CDH24	GFRA4	LCHN	TMEM50A	FUT11	HYAL2
BASP1	NAALADL1	TMEM74B	TSHR	AHCTF1	TPR	CAMP	SEC23A
SLC34A3	RGPD2	ZNRF2	FLRT2	RXFP1	ZNF431	DBP	ZBTB17
KIF9	OR2H2	SLC26A4	ARAP2	OR1J4	FAM150A	MSMP	AP2A2
PGBD4	LRRC8E	RANBP6	POU6F2	COQ10B	ABHD3	KJ904354	TMEM257
LRRC58	C1orf54	FN1	NLRC4	SMC4	AWAT2	OR52M1	CNTLN
GLTSCR1	TUBB8	RNF130	JHU18852	GSG1	RAI14	ACACB	CCDC34
LRRC75A-AS1	ZNF846	LEUTX	FOXD4L2	FFAR3	C6	ZBED4	TMEM184B
dJ402G11.C22.5	WDR64	NOBOX	PCDHGA4	ZNF335	MTMR11	CECR2	KCP
ZFAT	ZFY	ABCC10	ALDH18A1	IFT80	PBX1	FOXD3	GPHN
SMTN	SLC1A4	HIST1H2BL	ZNF212	ARSH	GAGE6	KRTAP5-8	SPATA33
SERPINA9	HAPLN2	DKK1	IGFBP2	FRA10AC1	CUL3	ZNF283	ZNF654
FABP2	CST8	TEK	LSG1	RIC8A	SNTG1	PCDHB15	KJ902540
OR13C8	KCNH4	LOC283951	SAG	TMEM52	PTGFRN	ATRNL1	FGF4
VPS54	RAB38	USP13	CASC5	FBXO45	ODF3B	ITGA4B7	CENPBD1

RFX8	OVOL3	CD38	PCYOX1	DMC1	RP3-400N23.9	MICAL3	C1orf61
DLL1	OR4C45	ZNF589	PLA2G12B	PTH2R	VAT1	UTP14C	PIP5K1C
ADGRL1	GEN1	NOS2	Q7tsi6	UBXN4	AMIGO1	SMYD1	TCAF2
PAX2	LAMC3	UCN	PSG5	AC145212.2	KCTD14	OTUD7A	HTRA1
LATS2	ZNF786	THY1	MTBP	RDM1	SOX17	TUBB4Q	RDH8
ADGRA3	C17orf50	HAP1	AVPR1B	FHOD3	PREX1	OR10G8	CRY1
CAPG	RPS5	DGAT2L6	ZNF292	TNRC6A	PADI1	ABCG8	KCTD3
NRL	NCOA1	MYL12A	HSPA6	FGFRL1	LCE2C	TTC9	BMP6
SPINK8	EXOC7	KRTAP19-1	DSG1	ABCC11	EVX1	C3orf62	PRDX6
TRMT61B	C9orf57	A0A096LNS0	KRTAP11-1	PAK3	RAB21	TSHZ3	TJP1
ARPC1A	MICALL1	OR4E2	OR1L1	HCN4	DMGDH	LECT2	MAP3K14
LRRFIP1	MRPS18C	WSB2	POP1	ZNF460	ZNF775	HAS1	CILP
HTRA3	CRYBB1	PANX2	KPRP	ADAM29	TXK	PNMAL2	ENOX2
IKBKAP	DPRX	KATNB1	MTMR10	MYPN	VEGF	COG4	FOXI2
TBX15	SLC6A1	CMC2	STC1	PIK3IP1	ZDHHC8	UPB1	EMX2
CNOT6L	PCDH11Y	BTN2A3P	CLTCL1	HDLBP	SPATA8	CABP5	CRTC1
MMP2	PTPRN2	PLEKHA6	RIMBP3	CDH17	ITGAE	KRTAP8-1	DRC7
CCNA1	TARBP1	NLRP14	PCP4L1	FCGR3B	HLA-DRB4	GEMIN7	LILRB5
MTHFR	GAL3ST1	CSPG4	PCDHA12	PCDHB11	PCDHGA9	IRF7	ZNF679
ZFPM2	LIG4	ST5	LGR4	KLF9	CLLU1OS	MTX3	KRTAP12-3
LIPM	JHU18929	PRSS33	SSTR5	SHC1	AQP10	KRTAP5-9	TFIP11
TEX40	GPC2	SMC5	PENK	UBALD2	OR7C2	KRT35	CLCA3P
RAB12	KIR2DS3	SHANK2	NOVA2	ABCC1	EIF1B	VCAM1	SRGAP3
ABCB5	ARHGAP44	ATXN1	PBX4	ZNF598	TBX4	ITGAX	RCAN3
RAB27B	KRTAP5-11	ARID3C	CRYBG3	GP1BB	KRTAP10-6	XRN2	SST
KIAA0368	KCTD5	CYLC2	DFNA5	FANCM	PLLP	TP53AIP1	C17orf80
ISLR	SOS1	ASIC5	BCORL1	ZNF391	PLEKHM3	OR51T1	GOLGA1
OGFOD1	SLC20A1	PHF1	ITPR3	XIRP1	RGS9BP	SENP7	TMEM161B
MYLIP	LTB4R	STARD6	KRT37	OPN1MW	ATP13A5	ITGAD	USP31
ZC3H13	OR6F1	ICAM3	OR2T1	ZFAND2B	LRRN2	DLEC1	IL31
KJ903674	CTCFL	BCLAF1	TFRC	KCNJ16			

Supplementary Table 10. Proteins that may bind to QPCT in the HuProt microarray

Name	Name	Name	Name	Name	Name
MAN1B1	NOSIP	NAP1L3	SPRR4	GPR85	PIN4
RUVBL2	SULT1C2	MED1	TSC22D3	ATIC	BC044592.1
TBXAS1	ILF2	ERF	SSBP3	RAB5B	RUVBL1
NME2	CDCA3	ADAMTSL4	SF3B4	ANXA9	C2orf27A
C11orf1	GAPDH	UBE2O	GSTA3	NLN	ZNF876P
MLF2	HNRNPA0	CCT7	TPH1	YPEL2	KDM1A
NAT9	ARF6	STK16	EAF2	SRRT	PXK
CCT3	CASS4	CSNK1G1	GNMT	CPT1A	UBE2D3
PSAT1	DCTN3	CTBP1	C1QTNF5	HTRA4	SPP1
NPM1	TK1	CXCL16	GPT2	ITPKB	IGKC
PRRC2B	AHSA1	SHMT1	KRTAP13-1	NCKIPSD	PRB3
PDCD2L	CKMT1A	NFYC	PIK3CA	BCAT1	DNAJA4
PRDX3	RUNDC3A	SDSL	TKFC	ADSSL1	Lin28a
THAP4	SULT1B1	RPL30	ANXA2	TNIP2	KIAA0895
NABP2	MPST	RPS6KA1	RBMS1	KBTBD6	DCAF8
ANXA3	NFKBIB	PLCD1	VGLL2	HBE1	KIZ
PSMA3	HEMK1	TAF6	GBGT1	ZNF69	KBTBD7
PRMT2	GFM2	GTF2H2	MAGEA9	GAMT	DLG3
SSBP2	DCTPP1	LIMCH1	PNP	NDUFS3	ALOXE3
COL4A3BP	BDH2	SOHLH2	CPNE3	PGP	SYNJ2BP
CAP2	CENPM	DNALI1	BEGAIN	HDAC6	CRYBB3
LSM6	FTL	ACOX1	ABHD16A	UBL7	EZR
FAM53C	PRKAR1A	HSPE1	JMJD6	HRAS	KLHDC9
ZCCHC7	PAPSS1	GSTM5	ALG13	LOC105372481	RRM1
ASS1	PYCR2	FAM49B	GAB1	MED4	PRB1
TRMT12	ASNS	FAM131B	KRTAP4-4	CBL	C5AR2
CKB	FAM131C	PSTPIP1	ADSS	PYCRL	GTF2I
RPL10A	HP	STIP1	TTC27	ZNF207	PPP6R2
NSDHL	RAB34	SPRR1B	CBLN4	SAMHD1	SUCLG2
DOK1	EWSR1	MAPK10	SPRR1A	NANS	IRF2BP2
SULT1A1	ISG20	ABCA8	NRBP2	LOR	ZNF154
ABI1	SULT1A3	YEATS4	DTNBP1	KJ902480.1	KRTAP2-3
METTL1	ANXA11	CLEC10A	F2	DUSP26	KRTAP6-2
ZCCHC10	NDUFS1	ADGRE1	DLG4	TLE4	SH3GLB2
NUP62CL	PDE9A	GALNT3	DCDC2	DNAI2	NAF1
MIF	PRAM1	EEF1G	ATP6V0D1	MGAT4EP	ENO1
HNRNPD	EAF1	HTATIP2	CBY1	MUM1L1	WIPF1
HNRNPC	MAPK8	VWA5B2	NUPL2	RAP1GDS1	METTL16
OLA1	GAD1	SSBP1	RTCA	NAPB	PCCA
ECHDC1	SSBP4	ZNF3	C1orf74	PABPC4	AKAP8

MORN1	TSSK3	RPUSD2	HSCB	BC073937.1	ACSL6
ALDOC	WDR54	PGPEP1	IRF2BP1	RALY	PILRA
DHODH	PTK2	IRS1	KCNAB1	HSP90B1	SMARCE1
ARG1	DNAJB6	FBXO2	ADD1	APIP	UBE2Q1
DDC	EPB41L2	ADAT3	ACSL4	ALKBH3	MBNL3
WBP2NL	IGHG1	KHDRBS3	NAMPT	VWA5A	AVEN
SARS	ALDH4A1	MCCC2	TFAP2D	NME4	EIF4G3
ADSL	PAPSS2	CDK6	SUV420H1	SMARCC1	ACO1
CDC37L1	RUNDC3B	INO80E	BRAT1	DAZ2	ME3
GLUL	MT1X	PRR35	TFAP2A	VGLL3	SHQ1
C11orf16	GSTA1	FAAH2	TFAP2E	DDX6	GMPR
AAR2	GCLM	HNRNPAB	FAM109B	GAS8-AS1	KJ902540
C11orf45	ROBO3	DAB1	ACOT7	SLC25A30	PAK4
CA3	SHCBP1	KJ901215	DTX2	KLC3	
IST1	KCNAB2	BC047522.1	ALDH16A1	PKM	
